# Global gene expression changes of *in vitro* stimulated human transformed germinal centre B cells as surrogate for oncogenic pathway activation in individual aggressive B cell lymphomas

**DOI:** 10.1186/1478-811X-10-43

**Published:** 2012-12-20

**Authors:** Alexandra Schrader, Katharina Meyer, Frederike von Bonin, Martina Vockerodt, Neele Walther, Elisabeth Hand, Antje Ulrich, Kamila Matulewicz, Dido Lenze, Michael Hummel, Arnd Kieser, Michael Engelke, Lorenz Trümper, Dieter Kube

**Affiliations:** 1 Department of Haematology and Oncology, University Medical Centre Göttingen, Göttingen, Germany; 2 Computational Diagnostics Group, Institute for Functional Genomics, University of Regensburg, Regensburg, Germany; 3 School of Cancer Sciences, University of Birmingham, Birmingham, UK; 4 Institute for Pathology, Campus Benjamin Franklin Charité Berlin, Berlin, Germany; 5 Research Unit Gene Vectors, Helmholtz Zentrum München - German Research Center for Environmental Health, München, Germany; 6 Department of Cellular and Molecular Immunology, University Medical Centre Göttingen, Göttingen, Germany; 7 Network “Molecular Mechanism of Malignant Lymphoma” (MMML) of the Deutsche Krebshilfe, Göttingen, Germany; 8 FOR942 of the Deutsche Forschungsgemeinschaft at the University Medical Centre Göttingen, Göttingen, Germany; 9 GRK 1034 of the Deutsche Forschungsgemeinschaft, Göttingen, Germany; 10 Network HaematoSys, Leipzig, Germany; 11 Department of Pediatrics I, University Medical Centre Göttingen, Göttingen, Germany; 12 Zentrum für Innere Medizin, Abteilung Hämatologie und Onkologie, Universitätsmedizin der Georg-August-Universität Göttingen, 37099, Göttingen, Germany

**Keywords:** Gene expression pattern, Lymphoma, Pathway activation

## Abstract

**Background:**

Aggressive Non-Hodgkin lymphomas (NHL) are a group of lymphomas derived from germinal centre B cells which display a heterogeneous pattern of oncogenic pathway activation. We postulate that specific immune response associated signalling, affecting gene transcription networks, may be associated with the activation of different oncogenic pathways in aggressive Non-Hodgkin lymphomas (NHL).

**Methodology:**

The B cell receptor (BCR), CD40, B-cell activating factor (BAFF)-receptors and Interleukin (IL) 21 receptor and Toll like receptor 4 (TLR4) were stimulated in human transformed germinal centre B cells by treatment with anti IgM F(ab)_2_-fragments, CD40L, BAFF, IL21 and LPS respectively. The changes in gene expression following the activation of Jak/STAT, NF-кB, MAPK, Ca^2+^ and PI3K signalling triggered by these stimuli was assessed using microarray analysis. The expression of top 100 genes which had a change in gene expression following stimulation was investigated in gene expression profiles of patients with Aggressive non-Hodgkin Lymphoma (NHL).

**Results:**

αIgM stimulation led to the largest number of changes in gene expression, affecting overall 6596 genes. While CD40L stimulation changed the expression of 1194 genes and IL21 stimulation affected 902 genes, only 283 and 129 genes were modulated by lipopolysaccharide or BAFF receptor stimulation, respectively. Interestingly, genes associated with a Burkitt-like phenotype, such as *MYC, BCL6 or LEF1, were affected by* αIgM*.* Unique and shared gene expression was delineated. NHL-patients were sorted according to their similarity in the expression of TOP100 affected genes to stimulated transformed germinal centre B cells The αIgM gene module discriminated individual DLBCL in a similar manner to CD40L or IL21 gene modules. DLBCLs with low module activation often carry chromosomal *MYC* aberrations. DLBCLs with high module activation show strong expression of genes involved in cell-cell communication, immune responses or negative feedback loops. Using chemical inhibitors for selected kinases we show that mitogen activated protein kinase- and phosphoinositide 3 kinase-signalling are dominantly involved in regulating genes included in the αIgM gene module.

**Conclusion:**

We provide an *in vitro* model system to investigate pathway activation in lymphomas. We defined the extent to which different immune response associated pathways are responsible for differences in gene expression which distinguish individual DLBCL cases. Our results support the view that tonic or constitutively active MAPK/ERK pathways are an important part of oncogenic signalling in NHL. The experimental model can now be applied to study the therapeutic potential of deregulated oncogenic pathways and to develop individual treatment strategies for lymphoma patients.

## Lay abstract

Aggressive Non-Hodgkin lymphomas (NHL) are a heterogeneous group of lymphomas derived from germinal centre B cells. 30% of NHL patients do not respond to treatment. Current criteria to distinguish individual NHL subtypes such as morphology, immunophenotype, and genetic abnormalities do not allow reliable subtype categorization and prediction of treatment response for NHL cases. The pathological mechanisms behind this heterogeneity are poorly understood. Thus there is a need of new and additional methods for stratifying NHL.

The purpose of our studies is to estimate the extent to which distinct signal transduction pathways could be responsible for the differences in gene expression that distinguish individual lymphomas. We postulate that signals associated with the immune response can resemble pathways activated in distinct NHL subtypes.

To gain closer insight into the relevance of distinct cell signaling networks to NHL subtypes, we stimulated human transformed germinal centre B cells with factors known to modify B cell signalling, or which are involved in B cell microenvironment or lymphoma pathogenesis. We discovered that coherent gene expression patterns, related to distinct in vitro stimuli, characterize individual NHLs. Exemplified by an αIgM stimulation we identified signalling pathways dominantly involved in regulating this consistent global gene expression pattern.

We provide an *in vitro* model system of pathways activated in transformed B cells which allows a better understanding of the global expression changes observed in particular lymphoma subgroups. This model can be used in the future to study the therapeutic potential of oncogenic pathway activation and to develop individual treatment strategies for patients.

## Background

Mature aggressive Non-Hodgkin lymphomas (NHL) are a heterogeneous group of lymphomas most often derived from B cells during the germinal centre B cell reaction [1-3]. Approximately 30 percent of patients with NHL classified as diffuse large B cell lymphoma (DLBCL) do not respond to treatment [4,5]. The criteria currently used to distinguish between Burkitt lymphoma (BL) and DLBCL, is based on differences in morphology, immunophenotype, and genetic abnormalities. These are not reliably reproducible and most importantly the pathological mechanisms behind these criteria are poorly understood [3]. NHL cells proliferate actively and retain many of the immunophenotypic characteristics of germinal centre B lymphocytes. However, they are monoclonal tumour B cells, and display characteristic nonrandom chromosomal abnormalities. Cellular genes thus can be placed under the control of heterologous promoter or enhancer elements and may switch off cellular growth regulation. In contrast, specific combinations of signals for short or long term stimulation are provided to germinal centre B (GC B) cells through externally derived signals obtained from cells in the microenvironment [1,6].

In peripheral secondary lymphoid organs B cells encounter foreign antigens. Antigen-stimulated B cells can in turn form germinal centres. In the microenvironment of germinal centres B cells need to interact with other cells, such as T cells, tingible body macrophages, follicular dendritic and reticular cells [1]. Signal transduction pathways initiated through the BCR determine the fate of B cells in dependence of BCR affinity to antigen, concomitant engagement of coreceptors and the differentiation stage of B cells [7]. GC B cells undergo apoptosis if not rescued through GC survival signals. However, unresolved chromosomal translocations and/or permanently deregulated autocrine or paracrine stimulations counteracting these processes can lead to transformation of GC B cells [1]. Within the GC B cell reaction or maintenance of mature B cells additional factors are involved including IL21, CD40L (TNFSF5 / CD154) or tumour necrosis factor superfamily member 13b (BAFF / TNFSF13b / CD257) [2,4-6,8]. In addition, there is evidence for an involvement of pattern recognition receptors in these processes [8]. It is well know from different cell systems that after treating cells with the mentioned stimuli a number of pathways are activated. This includes IL21-mediated modulation of janus-kinase (Jak) and signal transducer and activator of transcription (STAT) or mitogen activated kinases (MAPK)1/2 (Erk1/2) [8]. Furthermore, canonical and non-canonical nuclear factor-кB (NF-кB), MAPK8/9 (JNK1/2), MAPK14 (p38a) signalling is affected through CD40L, non-canonical NF-кB by BAFF, canonical NF-кB by LPS [8-12]. In addition Ca^2+^, phosphoinositide 3 kinase (PI3K), Erk1/2, canonical NF-кB, JNK1/2, p38a signalling can be initiated by B cell receptor activation [2,13-16]. In addition, aberrant signalling caused by a defined set of mutations or autocrine and paracrine loops for these pathways have been reported to be important for B cell lymphoma initiation or maintenance [2,11,17-19].

Recent large-scale gene expression profiling of NHL tumour samples revealed a molecular definition for BL, by describing a specific signature. This signature was used to model an index of ‘Burkitt-likeness’ (mBL-index) and to distinguish BLs from DLBCLs [20,21]. A fundamental question from these studies is the extent to which different pathways could be responsible for the differences in gene expression that distinguish individual DLBCL. We hypothesized that gene transcription networks affected by immune response associated signals resemble oncogenic pathway activity in DLBCL.

So far two major molecular patterns for DLBCLs are described: so called activated B cell (ABC) like lymphoma and germinal centre B cell (GC B) like lymphoma. They can be complemented by for example host response, stromal or even NF-кB specific gene expression signatures [22-25]. Recent combinations of *in vitro* cell interventions with systems biology allowed the prediction of potential oncogenic pathways involved in B cell transformation [26-28]. Furthermore, *in vitro* studies showed that combined STAT3 and NF-кB pathway activities are central to ABC-like lymphoma cells [22,29,30]. In addition, there is evidence that aberrant Toll like receptor (TLR) and BCR signalling may be involved affecting PI3K and/or MAPK/Erk signalling in addition to NF-кB [13,18,31,32]. These data are based mainly on interventions of constitutively activated pathways by knockdown experiments and mutational analysis [2,13,18].

To get more insight into cell signalling networks and their presence in individual human NHL, we utilized human transformed GC B cells. We demonstrate that B cell specific stimuli can be used to identify gene expression changes. This allows a “switch“ in gene expression from a steady state level characteristic of BL towards that of DLBCLs. Representative sets of genes (gene modules) are used to describe individual lymphomas. DLBCLs are heterogeneous in the appearance of the magnitude of their gene module activation ranging between “off” and “on”. Our data support the view that, for example, tonic and/or activated mitogen activated protein kinase- and phosphoinositide 3 kinase pathway components are part of a signalling network that distinguishes individual DLBCL. Furthermore, a useful *in vitro* model system to test for individual treatment strategies is offered.

## Results and discussion

### Global gene expression changes in human transformed germinal centre B cells stimulated with B cell specific paracrine stimuli

In order to achieve global gene expression changes to describe major pattern of gene expression and to identify pathway activity in aggressive NHL we used as our model system, the BL2 cell line, which is derived from germinal centre B cells [33-35]. BL2 cells were stimulated using CD40L, BAFF, IL21, αIgM F(ab)_2_ fragments or lipopolysaccharide (LPS) as described in Material and Methods section (Additional file 1: Supplementary Materials and Methods). These stimuli were chosen, because they are well known mediators of signalling in B cells, involved in GC B cell microenvironment and involved in B cell lymphoma initiation or maintenance [2,11,17-19]. Following stimulation, we wanted to identify gene expression changes which reflect pathways involved in ligand specific signal transduction and pathways potentially active in aggressive NHL. Time points of stimulations were chosen to achieve a signal strong enough to be detected as gene expression change at the whole genome level. Probes of three independent biological experiments were hybridized to U133 plus 2.0 microarrays. Differentially expressed genes were identified using linear models as implemented in the Bioconductor package LIMMA [36]. False discovery rates of differentially expressed genes were calculated according to the Benjamini and Hochberg in a paired-test as described in the Material and Methods section.

**Figure 1:**
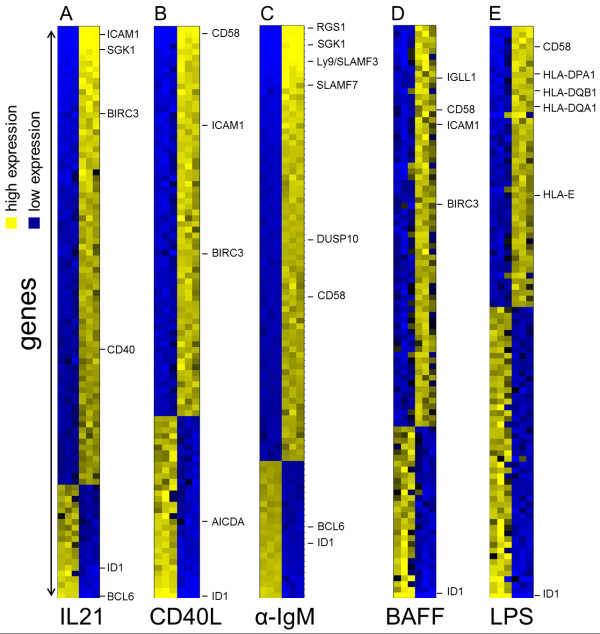
**Identification of IL21, CD40L, αIgM, BAFF and LPS regulated genes in transformed human germinal centre B cells using microarrays.** BL2 cell were stimulated with αIgM F(ab)_2_ fragments (3 hrs) (**A**), IL21 (2 hrs) (**B**), CD40L (6 hrs) (**C**), LPS (6 hrs) (**D**) and BAFF (9 hrs) (**E**). RNAs from these cells were used for gene expression profiling on Affymetrix HGU-133 plus 2.0 microarray chips. The heatmaps show the most highly changed gene expression (TOP100) (adj. p-value ≤ 0.05) in response to each stimulus. Each row in the heatmaps represents a gene and each column represents a microarray sample. Yellow and blue indicate high and low expression, respectively. Control probes (left column) are shown in comparison to stimulated probes (right column). Additional details are summarized within Table 3 and suppl. Tables 1–5.

Genes with the greatest change in expression and with an adjusted p value ≤ 0.05 in response to each stimulus were chosen for further analysis (Table 1 and Additional file 2: Table S1, Additional file 3: Table S2, Additional file 4: Table S3, Additional file 5: Table S4, Additional file 6: Table S5). The top 100 differentially expressed genes are depicted as heatmaps in Figure 1**.** To our knowledge the only comparable data set available is from human transformed germinal centre B cells (Ramos) which were cultivated on a CD40L expressing feeder cell line for 24 hours [37]. Despite the different experimental conditions, BL2 cells showed similar gene expression changes after exposure to recombinant CD40L for 6 hours (Additional file 7: Figure S1). In contrast, global gene expression changes after B cell receptor activation, for BAFF, LPS or IL21 stimulation have been described using different microarray-platforms. Therefore, a quantitative comparison is difficult. Furthermore, different cell lines or leukocyte cell subsets from a different origin, for example splenic murine B cells or bursal chicken B cells were analysed. A selection of available data is summarized in Additional file 8: Supplemental 1.

**Table 1:** Differential expression in human transformed germinal centre B cells in response to B cell-specific stimulations

	**α-IgM**	**CD40L**	**IL21**	**LPS**	**BAFF**
Upregulated genes	3039	689	463	114	69
Downregulated genes	3557	496	439	169	39
Total number of genes affected	6596	1194	902	283	129

BL2 cells stimulated through αIgM treatment, by CD40L, IL21, BAFF or LPS. RNA was hybridized onto U133A 2.0 plus Arrays. Differentially expressed genes between stimulated and control cells were identified using linear models as implemented in the bioconductor package LIMMA [36]. False discovery rates for lists of differentially expressed genes were calculated according to Benjamini and Hochberg [38]. Genes were ranked according to their p-value for differential expression from the microarray experiments (adj. P value ≤ 0.05).

### Gene set enrichment analyses of global gene expression changes in transformed germinal centre B cells

Molecular functions, biological processes, cellular components and pathways affected by distinct stimuli were characterized by gene ontology (GO) based gene set enrichment analyses (Additional file 9: Supplemental 2).

αIgM activated genes are linked to MAP kinase activity, phosphatase activity and transmembrane transporter activity. The biological processes affected can be summarized as regulation of immune responses, MAP kinase activity, and programmed cell death, regulation of metabolic processes (glucose / carboxylic acid / organic acid/amino acid transport) or cell cycle and stress responses.

IL21 activated genes are enriched for gene sets associated with responses to virus and other organisms and cytokine production including type I interferon biosynthetic processes. Furthermore, as for αIgM activated genes, IL21 affected gene sets are involved in regulation of programmed cell death. The involvement of IL21 activated genes on cytokine signalling could also explain its relation to IкB kinase/NF-кB cascade and NF-кB import into nucleus, gene sets characteristic for Toll like receptor pathways, Jak/STAT and chemokine signalling pathway, but also pathways in cancer are enriched. IL21 suppressed genes are characteristic for nucleotidyltransferase activity, cytoskeletal protein or phospholipid binding thus affecting cell shape, morphogenesis or chemotaxis.

BAFF activated genes are involved in metabolic processes of amino acids and chromatin remodelling, whereas downregulated genes are part of lipoprotein metabolic process, protein amino acid acylation.

The CD40L mediated gene expression changes positively affect MHC class I receptor activity and thus antigen processing and presentation of peptide antigen, the regulation of membrane potential, small GTPase mediated signal transduction as well as metabolic processes. In contrast, CD40L suppressed genes are involved in phospholipase activity or negative regulation of transcription.

### Gene expression changes in transformed germinal centre B cells of selected microarray results and validation by quantitative real-time PCR

Stimulation of BL2 cells led to changes in the expression of genes involved in cell-cell communications, including changes in *HLA, PECAM, CD1, CD86* or members of the signalling lymphocyte activation molecule family (SLAMF). Interestingly, expression of the *HLA* group of genes was positively regulated as a result of all stimulations. IL21 affects, for example *HLA-B, -C* and *-E* expression. The greatest upregulation was observed for *HLA-DPA1, -DQA1* and -*DQB1* following BAFF, CD40L and αIgM treatment. Furthermore, *CIITA* was activated by CD40L and αIgM. Expression of the *ICAM1* gene, which encodes a protein involved in cellular adhesion and costimulatory signalling and leukocyte trans-endothelial migration, is activated by all the stimuli used (Table 2 and Additional file 2: Table S1, Additional file 3: Table S2, Additional file 4: Table S3, Additional file 5: Table S4, Additional file 6: Table S5). IL21- treatment has the highest impact on *ICAM1* activation [39]. CD58, a ligand of CD2, is activated by CD40L and αIgM treatment [40].

**Table 2:** Gene expression changes in human transformed germinal centre B cells of selected microarray results

***Gene symbol***	***log***_***2***_***FC***					***Suggested function or pathway***
	**a-IgM**	**CD40L**	**IL21**	**BAFF**	**LPS**	
***CIITA***	0,79	0,81	-	-	-	Antigen presentation
***HLA-B***	0,32	0,83	0,26	0,57	0,51	Antigen presentation
***HLA-C***	0,34	0,71	0,28	0,52	0,5	Antigen presentation
***HLA-DPA1***	0,81	0,62	-	-	0,84	Antigen presentation
***HLA-DQA1***	1,54	1,13	-	0,75	0,74	Antigen presentation
***HLA-DQB1***	2,2	0,99	-	0,77	0,72	Antigen presentation
***HLA-E***	-	-	0,64	-	0,54	Antigen presentation
***BCL11A***	−0,82	-	−0,34	-	-	apoptosis
***BCL2***	1,63	-	-	-	-	apoptosis
***BCL2A1***	4,3	-	1,93	-	-	apoptosis
***BCL2L1***	0,41	-	0,89	-	-	apoptosis
***BCL2L11***	0,43	-	0,44	-	-	apoptosis
***BCL2L12,***	0,34	-	-	-	-	apoptosis
***CFLAR***	−0,43	0,74	0,54	0,45	-	apoptosis
***FAS***	0,91	0,83	0,89	-	-	apoptosis
***MCL1***	0,57	-	1,01	-	-	apoptosis
***CD1C***	2,67	-	-	-	-	Cell-cell communication, immune response
***CD58***	1,94	1,52	-	-	0,84	Cell-cell communication, immune response
***CD86***	1,16	−0,54	-	-	−0,34	Cell-cell communication, immune response
***ICAM1***	0,75	1,01	2,91	0,65	0,48	Cell adhesion
***PECAM***	−0,31	−0,56	-	-	−4,9	Cell adhesion
***SLAMF1***	0,47	-	-	-	-	Cell-cell communication, immune response
***SLAMF3***	4,14	−0,36	-	-	−0,42	Cell-cell communication, immune response
***SLAMF6***	−1,1	−0,48	−0,65	-	-	Cell-cell communication, immune response
***SLAMF7***	2,07	-	-	-	-	Cell-cell communication, immune response
***DUSP10***	2,17	-	0,89	-	-	Phosphatase activity, Negative regulation of MAPK
***DUSP2***	2,99	-	1,09	-	-	Phosphatase activity, Negative regulation of MAPK
***DUSP22***	0,74	0,98	-	-	0,53	Phosphatase activity, Negative regulation of MAPK
***RGS1***	5,5	-	-	-	-	Negative regulation of G-protein couppled receptors
***CXCL10***	-	0,4	1,63	-	-	chemokine
***CCR7***	3,6	-	0,57	-	-	Chemokine receptor
***CXCR5***	0,38	-	1,14	-	-	Chemokine receptor
***BCL6***	−2,03	−0,48	−1,94	-	-	Gene regulation, B cell differentiation
***BCOR***	−0,69	-	-	-	-	Gene regulation
***GCET2***	−0,95	−0,45	-	-	-	Gene regulation, B cell differentiation
***ID1***	−2,12	−2,04	−1,53	−2,1	−1,9	Gene regulation
***ID2***	0,74	-	-	-	-	Gene regulation
***ID3***	0,72	−1	−1,58	−0,56	−0,82	Gene regulation
***ID4***	−2,71	−0,83	−0,78	0,91	−0,79	Gene regulation
***IRF4***	1,53	0,46	2,14	-	0,58	Gene regulation, B cell differentiation
***LMO2***	1,45	-	0,53	-	-	Gene regulation, B cell differentiation
***PRDM1***	-	-	0,47	-	-	Gene regulation, B cell differentiation
***AICD***	−0,76	−0,34	0,82			Somatic hypermutation and class switch recombination
***AXIN1***	0,4	-	-	-	-	Wnt pathway
***BCL9***	−0,44	-	-	-	-	Wnt pathway
***DVL1***	−0,3	-	-	-	-	Wnt pathway
***FLI1***	−0,6	-	-	-	-	Wnt pathway
***FRAT1***	−0,37	-	-	-	-	Wnt pathway
***FRAT2***	−0,96	-	-	-	-	Wnt pathway
***FRZB***	−0,72	−0,39	-	-	-	Wnt pathway
***FZD2***	−0,56	−0,29	-	-	−0,28	Wnt pathway
***FZD3***	−0,81	-	-	-	-	Wnt pathway
***FZD6***	0,32	-	−0,3	-	-	Wnt pathway
***Kremen2***	-	0,35	-	-	-	Wnt pathway
***LEF1***	−0,85	-	-	-	−0,37	Gene regulation, Wnt pathway
***Myc***	−0,72	-	-	-	-	Gene regulation, Wnt pathway
***PYGO1***	1,13	-	-	-	-	Wnt pathway
***RAG2***	−0,55					VDJ-recombination
***TCF7***	-	0,44	-	-	-	Wnt pathway
***TLE3***	−0,66	−0,48	-	-	−0,35	Wnt pathway
***WNT10***	−1,37	-	-	-	-	Wnt pathway
***WNT3***	0,4	-	-	-	-	Wnt pathway
***WNT5a***	−0,72	0,93	-	-	-	Wnt pathway
***WNT5B***	-	0,5	-	-	-	Wnt pathway

A specific regulation of genes encoding for proteins involved in cellular adhesion and costimulatory signalling, cell-cell communications or antigen presentation and immunomodulation, cytotoxicity, humoral immunity, autoimmunity, cell survival, lymphocyte development, and cell adhesion, chemokine pathways, apoptosis or Wnt-signaling and GC B cell reaction regulation is revealed. Data are presented as log_2_FC from one probe set. Additional details are found in Additional file 2: Table S1, Additional file 3: Table S2, Additional file 4: Table S3, Additional file 5: Table S4, Additional file 6: Table S5.

SLAMF-associated proteins are important immunomodulatory receptors with roles in cytotoxicity, humoral immunity, autoimmunity, cell survival, lymphocyte development, and cell adhesion [41]. Whereas *SLAMF1*, *3* and *7* are strongly upregulated by BCR crosslinking, *SLAMF6* is inhibited. This inhibition is most prominent in response to αIgM. In contrast, CD40L treatment is associated with a decreased *SLAMF3* expression.

Defined elements of the chemokine system are specifically affected: IL21 upregulates *CCR7, CXCR5* and *CXCL10,* CD40L modulates the expression of *CCL5* (increased), *CCL17* (increased), *CXCR7* (decreased) and *CXCL10* (increased), whereas αIgM treatment affects *CCR7* (increased), *CXCR7* (decreased) and *CXCL10* (increased). The chemokine receptor CCR7, involved in germinal centre B cell homing is affected by CD40L but much stronger through αIgM [42]. CCR7 plays a pivotal role in homing of tumour cells into lymphoma-supporting niches in secondary lymphoid organs [43]. The chemokine *CXCL10* is involved in chemotaxis for monocytes and T lymphocytes and has been reported to play an important role in the pathogenesis of tissue necrosis and vascular damage [44].

The expression of the inhibitor of DNA binding 1 (*ID1*) is inhibited in response to IL21, CD40L, αIgM, BAFF or LPS treatment. The Id proteins are inhibitors of the basic-helix-loop-helix (bHLH) transcription factors [45]. In the B cell lineage, the *ID1* gene is usually expressed in pro-B cells and down regulated during differentiation [46]. Interestingly, inhibitors of DNA binding 1, 3 or 4 are inhibited by several stimulations. *ID3* expression is activated by αIgM, whereas the other stimuli are leading to an inhibition of *ID3*. *ID4* expression is not affected by IL21, whereas in all other cases it is inhibited.

The expression of BCL6, which is a central GC B cell reaction regulator, is inhibited in response to all stimuli [47]. However, the greatest effect was seen following treatment of cells with IL21 and αIgM. Furthermore, BCL6 interacting proteins, BCOR or BCL11A are also affected, by αIgM or CD40L treatment. Interestingly, this *BCL6* downregulation is accompanied by increased expression of *CXCL10* comparable to that described by Shaffer and colleagues [48]. In addition, *IRF4* is upregulated in response to all stimuli although for BAFF this was not significant. Termination of the GC reaction requires IRF4 as well as the transcriptional repressor Blimp1. IRF4 acts as a crucial transcriptional ‘switch’ in the generation of functionally competent plasma cells [49]. However, BLIMP1 (*PRDM1*) is only affected by IL21 (Additional file 3: Table S2) [50]. In addition, *LMO2* (LIM domain only 2) is activated by αIgM and IL21, a factor which also plays a central and crucial role in hematopoietic development and is highly conserved [51]. HGAL (*GCET2*) acting in concert with for example LMO2 or Bcl6 is suppressed by αIgM and CD40L treatment [52]. Interestingly, the expression of both AICD and RAG2 is inhibited by αIgM treatment.

Regarding the GO-analysis, genes involved in programmed cell death mainly affected by CD40L, αIgM and to some extend also by IL21. Thus, we observed changes in gene expression for example for *BCL2, BCL2A1* (BFL)*, BCL2L1* (BclXl), *BCL2L11* (BIM), *BCL2L12, CFLAR, FAS* or *MCL1)*.

**Figure 2:**
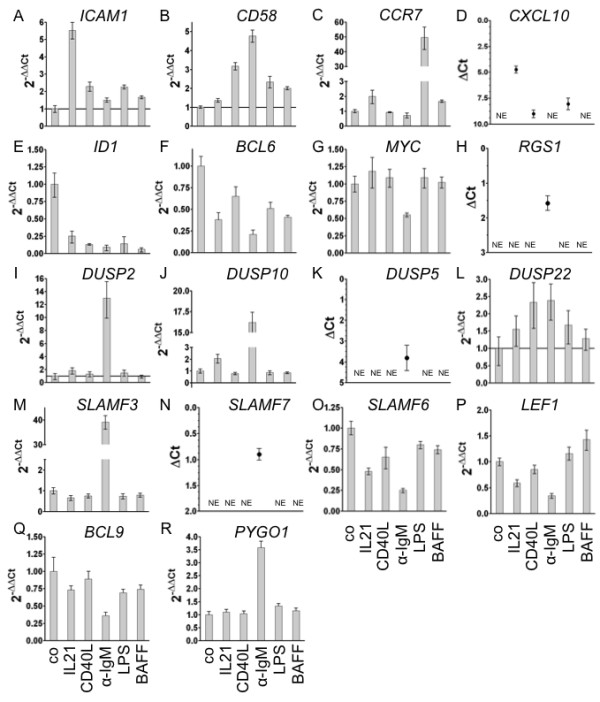
**Expression of a selection of genes after stimulation in transformed human germinal centre B cells as detected by qRT-PCR.** BL2 cells were stimulated as described in Figure 2. One representative experiment out of two is shown. All samples were analysed in triplicates. Expression of *ICAM1*, *CD58*, *CCR7*, *CXCL10*, *ID1, BCL6*, *MYC*, *RGS1*, *DUSP2, -10, -5, -22, SLAMF −3, -7, -6, LEF1*, *BCL9*, *PYGO1* is shown as 2^-ΔΔCT^ or ΔCT values, relative to abl housekeeper expression and compared to unstimulated control. *CXCL10* (D), *RGS1* (H), *DUSP5* (K) and *SLAMF7* (N) show no detectable expression in unstimulated cells (basal ΔCt > 10). Therefore, expression is depicted as ΔCt values. N.E. = Not Expressed.

Gene expression changes in response to IL21, CD40L, αIgM, BAFF and LPS were also measured by quantitative real time PCR (Figure 2). As exemplified for *ICAM1, CD58, CCR7, CXCL10, ID1, BCL6, MYC, RGS1, DUSPs* and *SLAMF* members (Figure 2A-O) an overall good agreement of qRT-PCR data with the microarray data is observed.

### Elements of the Wnt pathway are affected by in vitro interventions

*LEF1* was recently defined as a signature gene in defining the index of Burkitt-likeness [20]. Thus, we investigated changes in the expression of Wnt-pathway components. Interestingly, αIgM stimulation led to reduced *LEF1* expression (Figure 2P). The same was observed for *BCL9. PYGO1* expression was elevated in response to BCR activation. This was verified by qRT-PCR analysis**.** Comparable to the stimulation effect on *LEF1* expression, we verified the dominant effect of αIgM treatment on *BCL9 (down) and PYGO1 (up)* (Figure 2Q, R). Furthermore, *AXIN1, FZD2, 3, 6, FRAT1, 2* or *DVL1, FLI1, TLE3, FRZB, WNT3, 5A, 10* were changed to a lesser extent by αIgM. This is an important observation because Wnt5a produced by follicular dendritic cells affects the B cell differentiation program of germinal centre B cells [53]. The expression of *FZD6* and *WNT5a* are modulated by IL21 and *TLE3* by LPS. In addition, CD40L modulates the expression of *FRZB, KREMEN2, TCF7, TLE3* and *WNT5A***(**Table 2**)**. Therefore, we conclude that αIgM stimulation affects major signature genes such as MYC and LEF1 defining the index of Burkitt-likeness [20].

### IL21, CD40L, αIgM, BAFF and LPS affected gene expression changes: similarity and uniqueness

**Figure 3:**
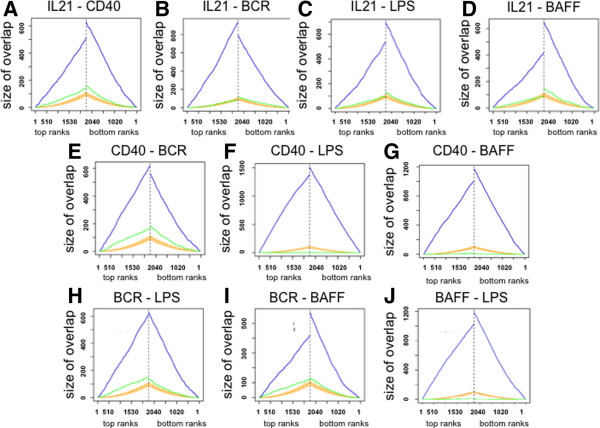
**Overlap in global gene expression of IL-21, CD40L, αIgM, BAFF and LPS affected genes.** BL2 cells were stimulated and data were processed as described in the text. The overlaps of differentially expressed gene lists at both ends were compared using the “Ordered List package” [55]. Genes affected by the stimuli assigned on top of each plot are compared. The gene lists are compared in the same (blue line) or reversed order (green line). The y-axis gives the size of the overlap in the number of genes of the top n-genes (x-axis) in both gene lists. The orange line gives the expected size of a random overlap, and the vertical orange lines indicate 95% probability intervals of random overlaps. All overlaps show a high statistical significance (α < 0.005). Additional details are summarized within Additional file 10: Table S6, Additional file 11: Table S7, Additional file 12: Table S8, Additional file 13: Table S9, Additional file 14: Table S10, Additional file 15: Table S11, Additional file 16: Table S12, Additional file 17: Table S13, Additional file 18: Table S14, Additional file 19: Table S15.

In order to describe similarities in gene expression the global responses to the stimuli were analysed by the *Ordered List approach* (Figure 3) [54]. In this approach, genes were ranked according to their fold change in response to respective stimulation. Pairwise comparisons of top and bottom ranks of lists representing IL21, CD40L, αIgM, BAFF and LPS responses were plotted. We observed a high overlap of genes responding in the same manner for each pairwise comparison (p < 0.005). This can be seen in Figure 3 by the difference between the blue line, representing the number of overlapping genes at the corresponding position of the gene lists given and the orange area giving the expected size of a random overlap. The gene lists are also compared in reversed order represented by the green line. The genes are summarized within the supplementary information (Additional file 10: Table S6, Additional file 11: Table S7, Additional file 12: Table S8, Additional file 13: Table S9, Additional file 14: Table S10, Additional file 15: Table S11, Additional file 16: Table S12, Additional file 17: Table S13, Additional file 18: Table S14, Additional file 19: Table S15). The strongest overlap was observed for IL21 and αIgM. This is somehow surprising since it was suggested that the shared NFкB driven gene expression changes mediated by LPS, CD40L, αIgM or BAFF would be dominant in defining the major pattern of gene expression changes. However, the strong overlap of IL21 with αIgM is also reflected in the GO analysis, showing that IL21 and αIgM gene expression changes are enriched for positive regulation of the IкB kinase/NF-кB cascade, RNA metabolic processes or immune system processes but also DNA-repair (Additional file 20: Supplemental 3). The shared functions of CD40L and αIgM affected genes are for example characterized by immune response, antigen processing and presentation or positive regulation of B cell activation, BMP signalling pathway and phosphate metabolic processes.

In addition, we describe genes that are specifically affected only by one of the utilized stimuli (uniqueness) (Additional file 21: Table S16). Interestingly, those genes which are dominantly affected by αIgM treatment are part of biological processes such as nucleic acid binding, PI3K regulator activity, regulation of cell cycle or metabolic processes, Wnt receptor signalling pathways and response to hypoxia (Additional file 22: Supplemental 4).

Therefore, our data now provide a comprehensive collection of gene expression changes induced by different physiological stimuli. These data sets can be used for a better understanding of gene expression changes in B cell signalling and lymphoma as we will show below. An *in vitro* model system will be tested to investigate pathway activations in individual DLBCL.

### Coherent gene expression of αIgM affected genes characterizes individual NHL

To further underpin the functional relevance of the gene expression changes observed following treatment with the stimuli, we investigated whether the change in expression of these genes is comparable to primary NHL. Two independent patient cohorts were included. The gene expression profile from 219 primary tumour samples described by Hummel et al. (MMML1 cohort) and 99 published by Dave *et al.* (LLMPP cohort) were compared to the gene expression changes described above [20,21]. The genes were summarized in Table 3. In some cases less genes were used because they were missing on the microarrays used for lymphoma gene expression analysis.

**Table 3:** **Most prominent altered genes after*in vitro*interventions of human transformed germinal centre B cells investigated in gene expression profiles of primary samples of aggressive Non-Hodgkin Lymphoma**

	**BAFF**		**CD40L**		**anti-IgM**		**IL21**		**LPS**	
	**Gene symbols**	**log2FC**	**Gene symbols**	**log2FC**	**Gene symbols**	**log2FC**	**Gene symbols**	**log2FC**	**Gene symbols**	**log2FC**
**1**	ID1	−2,07	ID1	−2,007	CYP26A1	−2,752	BCL6	−1,904	ID1	−1,9
**2**	ID4	−0,92	C3orf37	−1,432	ID4	−2,687	SOX2*	−1,805	RGS18*	−0,853
**3**	BNIP3	−0,79	SMAD1*	−1,182	BEST3*	−2,41	DNAJB4	−1,589	C3orf37	−0,85
**4**	TXNIP	−0,74	DEPTOR	−1,159	IL7R*	−2,399	ID3	−1,534	DDIT4	−0,83
**5**	BNIP3L	−0,72	CAB39L	−1,086	BMP7	−2,373	DDIT4	−1,514	ID3	−0,811
**6**	PFKFB4*	−0,71	CD83	−1,017	SOX4	−2,343	ID1	−1,493	METTL7A	−0,8
**7**	GPER	−0,68	METTL7A	−1,005	TNFSF8*	−2,264	RGCC	−1,239	CAB39L*	−0,799
**8**	PNOC	−0,67	ANKRD36BP2*	−0,983	RNF144B*	−2,213	IL7R	−1,152	TXNIP	−0,786
**9**	RGS18*	−0,66	ID3	−0,983	DNAJB4	−2,156	HEY2*	−1,136	BNIP3	−0,776
**10**	P4HA1	−0,654	ENPP3*	−0,937	ID1	−2,093	VEGFA	−1,115	ID4	−0,776
**11**	CCNG2	−0,635	IRF8	−0,924	BCL6	−2,037	GADD45A	−0,982	CCNG2	−0,721
**12**	HILPDA	−0,631	RGS18*	−0,919	RHOH	−2,005	CYTIP	−0,93	PFKFB4*	−0,709
**13**	ID2	−0,585	FAM167A*	−0,909	RGCC	−1,946	PCDH9	−0,914	KDM3A	−0,69
**14**	SAMD13*	−0,582	AICDA	−0,864	NCKAP1	−1,857	CCNG2	−0,889	HILPDA*	−0,678
**15**	CD24	−0,578	GPER	−0,852	IKZF1*	−1,845	TXNIP	−0,872	FAM167A*	−0,673
**16**	ID3	−0,564	ID4	−0,835	HEY2*	−1,761	BICD2	−0,868	PTPRC	−0,665
**17**	C3orf37	−0,56	PDZRN4	−0,809	PCDH9	−1,751	RGS18*	−0,861	STAT2*	−0,649
**18**	GPR18	−0,541	BNIP3	−0,785	SOX2*	−1,723	ANKRD37*	−0,852	SAMD13*	−0,631
**19**	ALDOC	−0,516	VEGFA	−0,777	MYCT1*	−1,716	TRIM8	−0,836	ENPP3*	−0,609
**20**	BBOX1	−0,516	TP53INP1*	−0,776	RGS18*	−1,715	KIAA0907	−0,786	SMAD1*	−0,593
**21**	CD83	−0,509	PTPRC	−0,77	SMAD1	−1,679	NEDD9	0,787	P4HA1	−0,593
**22**	POLD4	−0,494	RASSF6*	−0,767	MLLT3	−1,656	RELB	0,79	VEGFA	−0,592
**23**	HBP1	−0,484	CCNG2	−0,761	KIF20A	−1,631	PAPD5*	0,791	KLRC3	−0,591
**24**	FAM167A*	−0,476	CLEC2B	−0,74	FAM214A*	−1,612	ZNFX1*	0,794	GPER	−0,588
**25**	EVI2A	−0,476	BMP7	−0,735	BCL2	1,618	B3GNT2*	0,797	ZNF385B*	−0,588
**26**	TP53INP1*	−0,474	TLR1	−0,732	PSAT1*	1,62	HIF1A	0,814	TP53INP1*	−0,583
**27**	YPEL5	−0,467	SATB1	−0,731	LOC285628*	1,637	KIF26B	0,814	UBE2H*	−0,581
**28**	TRIM22	−0,464	BTG2	−0,726	FAIM3	1,641	MMD	0,82	TRIM22	−0,571
**29**	TLR1	−0,463	PCDH9	−0,725	MAP2K3	1,644	AICDA*	0,822	PIK3C2B	−0,571
**30**	CLEC2B	−0,463	PRKACB	−0,724	PPP1R15A	1,662	EPSTI1*	0,837	STAP1	−0,564
**31**	HLA-C	0,457	GPM6A	−0,721	FAM208B	1,674	RAB11FIP1	0,844	BNIP3L	−0,558
**32**	ACTR3*	0,46	STAT2*	−0,72	SIAH2	1,676	RAB30	0,845	MYBL1	−0,556
**33**	NAF1*	0,46	CLIP2	0,721	HIVEP3*	1,679	SIN3A*	0,847	GPM6A	−0,549
**34**	PSMD12	0,461	ARHGAP17	0,727	METRNL*	1,711	PLEK	0,854	ANKRD37*	−0,548
**35**	NUDT5*	0,461	CFLAR	0,74	HERPUD1	1,727	IFITM1	0,863	SPG11	−0,537
**36**	TESC	0,463	FYTTD1*	0,743	KLF10	1,749	PRDM2	0,885	NARF	−0,536
**37**	CCAR1*	0,464	HLA-DMB	0,747	LTA	1,753	DUSP10	0,888	ABCA1	−0,531
**38**	ANKRD33B*	0,465	MTMR10*	0,749	GPR18	1,769	FAM100B*	0,892	SEC14L1	−0,531
**39**	RILPL2*	0,466	HLA-F	0,754	IFRD1	1,777	FAS	0,894	IL7R	−0,522
**40**	PLCB1	0,467	PLEK	0,757	CD274*	1,785	IL7	0,896	H1FX	−0,52
**41**	NFKBIE	0,47	PPP1R9B*	0,759	SLC3A2	1,79	CD69	0,921	ASF1A	−0,511
**42**	DDX21	0,47	TESC	0,764	HSPA5	1,8	B4GALT1*	0,923	GLCCI1*	−0,509
**43**	TAPBP	0,471	IGLL1	0,765	ATF3	1,834	BCL2L1	0,924	EVI2A	−0,508
**44**	RBBP6	0,474	SNX20*	0,765	DENND4A	1,848	CD40	0,934	VGLL4	−0,507
**45**	SOGA2	0,474	CREM*	0,77	ARHGAP25	1,849	TET3*	0,951	PRKACB	−0,503
**46**	HINT1*	0,474	IKBKE	0,782	TOR3A	1,85	SOCS1	0,962	LRP4	−0,499
**47**	SRRM1	0,475	FAS	0,788	IRF2BP2*	1,868	NFKBID*	0,983	PLCXD1	−0,498
**48**	PHACTR4	0,475	CIITA	0,788	SLC30A1	1,886	SRSF5	0,996	CLEC2B	−0,497
**49**	AK3*	0,476	TAP1	0,789	TNF	1,905	MASTL*	1,011	LOC440864*	−0,494
**50**	KPNB1	0,477	NOTCH2NL*	0,797	CTH	1,91	TLR7	1,036	SLC44A1*	−0,493
**51**	GTF2H2B*	0,477	RFX5	0,806	APOBEC3B	1,928	MCL1	1,04	TSPAN11*	−0,484
**52**	TAF1D	0,477	FDXR	0,81	NFKB1	1,94	PIM2	1,06	BTN2A2	0,488
**53**	CSF2RB	0,479	NFKBIE	0,827	CD58	1,946	IER2	1,067	NFKBIE	0,493
**54**	MYBBP1A	0,481	MIR155HG*	0,829	NFKBIE	1,958	ZFP36L1	1,072	HLA-C	0,495
**55**	YY1*	0,483	LOXL2	0,829	NAB2	1,984	IFIT2*	1,072	LSS	0,502
**56**	FLVCR1*	0,488	HLA-B	0,831	JUND	2,024	SNX11	1,078	FDXR	0,502
**57**	NAA16	0,49	VAV2*	0,832	ARL4C	2,026	TAP1	1,086	KLHDC5*	0,505
**58**	SRRT	0,491	ZNF385C*	0,836	UPP1	2,029	JUNB	1,088	HLA-B	0,511
**59**	TNRC6A*	0,493	RASSF2	0,841	CDKN1A	2,033	LRRC32	1,1	CCDC28B	0,514
**60**	FNBP1	0,494	HLA-E	0,843	FAM100B*	2,059	NA	1,101	HLA-DMB	0,517
**61**	CIZ1	0,495	BIRC3	0,845	MDFIC	2,091	MARCKS	1,108	CUX2	0,519
**62**	TOMM40	0,495	ANKRD33B*	0,847	IER2	2,137	DUSP2	1,134	DUSP22*	0,523
**63**	TBC1D24*	0,498	ANXA7	0,85	DUSP10	2,144	NUDT4	1,139	RPS6KA1	0,527
**64**	ILF3	0,498	HVCN1*	0,85	FAM102A	2,198	CXCR5	1,155	HLA-DOA*	0,527
**65**	NFKB2	0,501	EPB41L4B*	0,86	RAB8B*	2,198	IER5	1,155	SYNCRIP	0,528
**66**	TFDP1*	0,505	PIK3CD	0,864	PTGER4	2,204	BCL3	1,16	ZNF385C*	0,533
**67**	ZNF385C*	0,508	LAT2	0,871	HLA-DQB1	2,206	SAMSN1	1,18	DENND4A	0,535
**68**	LYAR*	0,508	HCP5	0,884	ARAP2	2,214	NFKBIZ*	1,2	PDLIM3*	0,535
**69**	BIRC3	0,508	HLA-DOA*	0,894	SLC7A11	2,215	IL2RA	1,211	FYTTD1*	0,536
**70**	LRP8	0,513	SYNGR2	0,901	MIR155HG*	2,249	PARP9*	1,212	ELL2*	0,537
**71**	CCDC28B	0,514	ALCAM	0,905	CD69	2,278	USP18	1,229	HLA-E	0,539
**72**	C8orf12*	0,514	WNT5A	0,916	TSC22D3	2,287	MAP3K8	1,333	IRF2BP2*	0,539
**73**	HLA-B	0,519	RUNX3	0,932	RGS16	2,316	ZC3H12A	1,38	DNAJB1	0,54
**74**	HSP90B1	0,523	BMP2K*	0,935	ZFP36L1	2,331	NFKBIA	1,435	IGLL1	0,545
**75**	KLHDC5*	0,525	CTSH	0,935	TRIB3	2,354	RGS16	1,437	TFDP1*	0,558
**76**	RP9*	0,526	PLEKHO1	0,94	IL21R*	2,373	GADD45B	1,481	PSPC1*	0,56
**77**	PEA15	0,528	RPS6KA1	0,956	KLF6*	2,383	MIR155HG*	1,498	ANKRD33B*	0,562
**78**	MT1X	0,534	DUSP22	0,963	PHACTR1	2,423	LINC00158*	1,525	CREBZF	0,565
**79**	MAN1A1	0,539	IFIH1	0,991	SESN2*	2,446	PTGER4	1,563	IRF4	0,575
**80**	DNAJB1	0,543	OLFML2A	0,994	TNFAIP3	2,612	DTX3L*	1,58	C8orf12*	0,579
**81**	SOGA1*	0,554	HLA-DQB1	0,994	CD1C	2,662	CXCL10	1,616	FNBP1	0,617
**82**	CENPV*	0,555	COL1A1*	1,003	PLEK	2,716	TNFAIP3	1,636	ANXA7	0,641
**83**	ICAM1	0,567	ICAM1	1,006	FAM46C*	2,926	MX1	1,638	MT1X	0,67
**84**	CD58	0,568	NFKB2	1,009	CD83	2,973	STAT1	1,659	BATF	0,702
**85**	NCOR2	0,578	NEIL2*	1,021	DDIT3	2,977	BIRC3	1,66	CALR	0,703
**86**	SNHG12*	0,585	FNBP1	1,041	DUSP2	3,004	EGR2	1,69	HLA-DQA1*	0,706
**87**	SLC23A2	0,586	BATF	1,061	SQSTM1	3,036	NFKBIE	1,765	OLFML2A	0,714
**88**	OLFML2A	0,588	CCDC28B	1,066	PHLDA1	3,048	IFIT5	1,913	UMODL1*	0,716
**89**	LRRFIP1	0,59	MAN1A1	1,082	BHLHE40	3,141	BCL2A1	1,942	HLA-DQB1	0,723
**90**	DENND4A	0,599	CUX2	1,089	SLAMF7*	3,147	IRF1	1,965	IKBKE	0,723
**91**	IGLL1	0,614	HLA-DQA1*	1,092	CCR7	3,572	SLC30A1	1,969	NEIL2*	0,726
**92**	OVOS2*	0,63	DENND4A	1,101	NA*	3,693	IFIT3*	1,973	RILPL2*	0,779
**93**	C1orf63	0,649	HLA-DPA1	1,105	LINC00158*	3,798	IRF4	2,167	MAN1A1	0,781
**94**	EIF5B	0,681	RILPL2*	1,117	LY9*	4,131	IFIT1	2,186	CREM	0,795
**95**	RUNX3	0,702	DOCK10	1,202	EGR1*	4,232	CMPK2*	2,253	HLA-DPA1	0,816
**96**	NA	0,704	BTN2A2	1,254	BCL2A1	4,295	BATF	2,3	ACTR3*	0,833
**97**	HLA-DQB1	0,726	NA	1,262	EGR2	4,518	CD83	2,705	CD58	0,834
**98**	HLA-DQA1	0,731	ELL2*	1,467	SGK1	4,84	SGK1	2,798	NA	0,952
**99**	TCOF1	0,744	CD58	1,62	DUSP5	5,011	ICAM1	2,918	LINC00158*	0,954
**100**	ELL2*	0,747	LINC00158*	1,793	RGS1	5,476	IRF9	3,155	IFIT3*	1,051

Two independent patients cohort were tested. The gene expression from 219 primary lymphoma samples described by Hummel et al. (MMML-1 cohort) and 99 primary lymphoma samples published by Dave et al. (LLMPP-cohort) were compared to global gene expression changes described within the text [20,21]. The deviation from the TOP100 LIMMA gene lists from *in vitro* interventions is determined by the absence of hybridization probes on the microarrays used for lymphoma gene expression description. In addition multiple hybridization probes for one gene were condensed into a single event.

**Figure 4:**
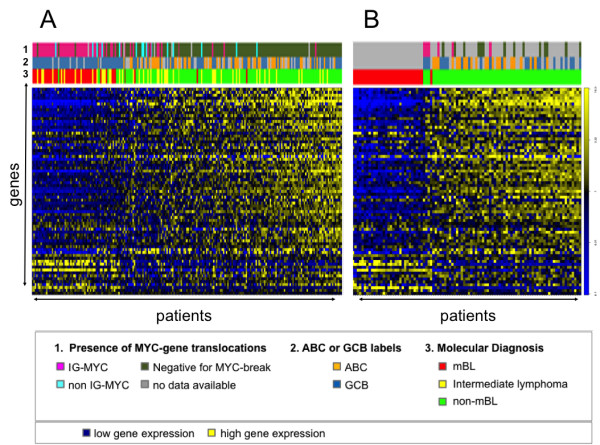
**Changes in global gene expression separates Burkitt lymphoma and diffuse large B cell lymphoma.** The expression of the TOP100 most prominent responding genes upon stimulation of BL2 with αIgM F(ab)_2_ fragments was investigated in primary lymphoma. Left panel: 220 lymphoma cases [20]; right panel: 99 lymphoma cases [21]. NHL cases were ordered from left to right according to their expression of αIgM affected genes in BL2 cells. The Heatmaps display the expression of target genes (columns) across lymphoma samples (rows). The lower colour bar above the heatmaps marks mBL in red, non-mBL in green and intermediate lymphoma in yellow (molecular diagnosis). The affiliation of samples to ABC/GCB DLBCL subgroups and the presence of an *IG-MYC* translocation is encoded in the middle and upper bar on top of the map (see legend for colour coding). Relative gene expression is encoded with yellow (high expression) and blue (low expression). As different microarrays were used for the whole genome expression analyses of cell perturbation and patient samples (Affymetrix HGU-133A and HGU133 plus2.0), the list of TOP100 genes had to be adapted to be able to transfer the resulting genes to patient data (see also Table 3, Material and Methods section and Supplementary material for additional details).

αIgM driven gene expression changes had the greatest absolute fold changes therefore we started with these. The expression levels of a list of 100 genes with a FDR < 0.1 were examined in clinical lymphoma samples. Their joint expression was estimated using a standard additive model fitted by Tuckey’s median polish procedure. These gene groups are further referred to as gene modules. The αIgM gene module can be used to differentiate BLs from DLBCLs shown in a heatmap (Figure 4A**)**[20]. On top of the heatmap are labels for the molecular classification (index of Burkitt-likeness, molecular ABC/GCB-classification) and the presence of a chromosomal translocation of *MYC*. Patients from the MMML1 cohort are sorted according to their increase in the expression of genes from the gene module. On the right part of the heatmap lymphomas are depicted characterized by a high expression of genes reflecting an increased expression of genes building the αIgM gene module. Lymphoma cases represented on the left side of the heatmap are characterized by gene expression comparable to unstimulated cells *in vitro* (BL2). Note that the genes are coherently expressed across lymphoma. There is a continuous gradient when lymphomas are arranged by increasing expression of genes from the αIgM gene module. Thus, the global gene expression change is absent or present in individual lymphomas (Figure 4A). Most BLs are characterized by the absence or low expression of the αIgM gene module and thus lack corresponding pathway activities. This is also observed in the LLMPP cohort (Figure 4B) [21]. Therefore, it is reasonable to believe that individual lymphomas with a high gene module expression are characterized by a stronger activation of oncogenic pathways than those with a low expression of same genes. Therefore human transformed GC B cells (BL2) can be defined as a suitable *in vitro* model used as surrogate for pathway activity.

### Gene modules of IL21, CD40L or αIgM is almost perfectly discriminate individual DLBCL

**Figure 5:**
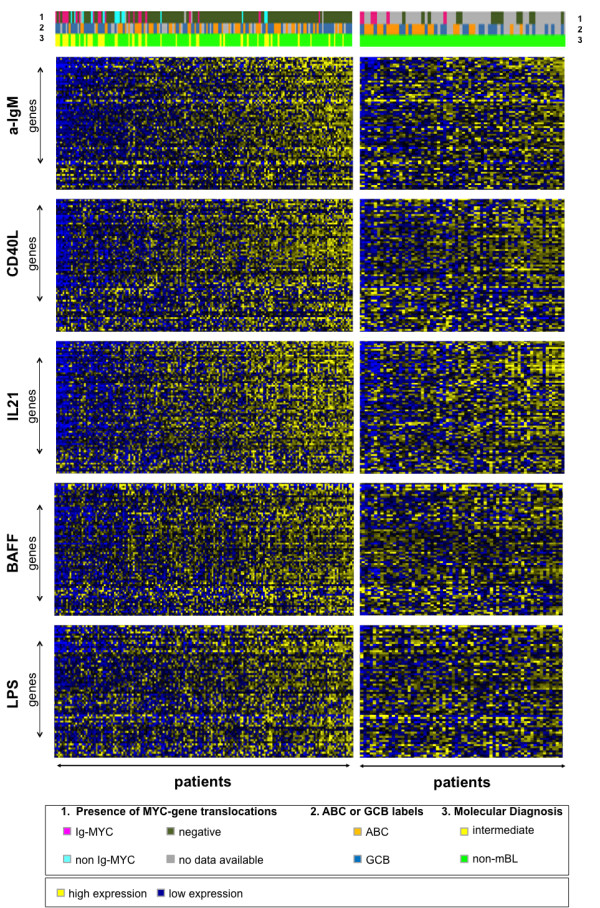
**Individual diffuse large B cell lymphomas are characterized by a specific activation of gene modules.** The TOP100 most prominent responding genes upon stimulation of BL2 with αIgM, CD40L, IL21, LPS and BAFF (see also Table 1) were investigated for their expression in the gene expression profiles of two distinct datasets of primary lymphoma. (A) left panel: 175 DLBCLs [20]; right panel: 99 DLBCL cases [21]. DLBCL cases were ordered from left to right according to the similarity of gene expression to the stimulated status of BL2 cells. The Heatmaps display the expression of target genes (columns) across lymphoma samples (rows). The colour bar above the heatmaps marks mBL in red, non-mBL in green and intermediate lymphoma in yellow. The affiliation of samples to ABC/GCB DLBCL subgroups and the presence of an *IG-MYC* translocation in encoded in a bar on top of the map (see legend for colour coding). Relative gene expression is encoded with yellow (high expression) and blue (low expression). Additional details on statistical significant differences are summarized within Additional file 7: Figure S1.

As BLs are discriminated on the molecular level from other lymphomas as shown by us and Dave et al. [20,21], we next focused on gene expression changes mediated by BAFF, LPS, IL21 or CD40L *in vitro* in comparison to αIgM in individual DLBCLs (Figure 5). DLBCL cases were arranged according to the activity of the αIgM gene module. The genes are coherently expressed across lymphomas and there is a continuous gradient when lymphomas are arranged by their increase in the expression of genes from the gene module of IL21 or CD40L in a comparable way as αIgM. This holds also true for the BAFF/LPS driven gene modules within the MMML1 cohort. This highly significant difference is observed by comparing lymphoma cases from the MMML-1 cohort by describing three main groups with low, intermediate and high module activation using corresponding box plots (Additional file 23: Figure S2). The differences are highly significant with respective p-values: p < 2.2e-16 / p = 1.669e-10 (αIgM), p < 2.2e-16 / p = 9.1e-07 (CD40L), p < 2.2e-16 / p = 5.9e-08 (IL21), p < 2.2e-16 / p = 2.614e-05 (BAFF), p < 2.2e-16 / p = 1.6e-4 (LPS) in MMML or LLMPP samples.

The comparison of our data with the recently defined groups of ABC-like or GCB-like DLBCLs reveals no direct association with one of the gene modules presented here (Figure 5, labels on top of the heatmaps) [22]. At the same time, DLBCLs with a *MYC* translocation are characterized by low gene module activation. Lymphomas carrying a *MYC* break are absent in those patients characterized by a higher activation of gene modules. Importantly, DLBCLs characterized by a very high gene module activation show evidence for the expression of genes involved in cell-cell communication or immune responses as well as negative feedback regulatory loops as RGSs and DUSPs [56,57]. A different expression of genes involved in cell-cell communication or immune responses in GCB-like DLBCLs may suggest a different capacity of lymphoma cells to evade immune responses of the host. Furthermore, the activation of negative feedback loops suggests, that although gene modules are typical for acutely activated genes, their outcome seems to be a balance of activating and suppressing signals. These signals imply strong oncogenic pathway activation but also damped cellular activity due to diverse negative feedback reactions or still present tumor suppressor activities.

Highly activated *CD58* is part of gene expression changes defined by four stimuli and may present an important marker for DLBCLs. This is in line with recent observations from transcriptome sequencing of DLBCLs. A significant number of DLBCL mutations were identified affecting the CD58 gene [58]. It was suggested that these mutations might play a role in the escape from immune-surveillance of these lymphomas [58,59]. Therefore, it is tempting to speculate that DLBCL with high CD58 expression would be less efficient in immune escape compared to those with reduced CD58 expression or loss of expression due to genetic alterations in this gene. This is also in agreement with our GO analysis, suggesting strong effects on antigen presentation. This is further supported by the expression changes of HLA molecules.

The DUSP family is a set of molecular control molecules which modulate MAPK signalling. DUSPs are affected by all stimuli and also present in the gene modules identified. Their role, either as phosphatases or scaffold proteins, remains to be elucidated as they are involved in defining the magnitude of pathway activity in DLBCLs. The same holds true for the SLAMFs. They play an essential and non-redundant role in the control of humoral immune responses. It would be interesting to investigate whether their expression is functionally linked to the recently observed aberrations in *CD58* or *ß2M* in DLBCLs that might be involved in differences in the capacity to escape host immune responses [41,58].

RGS1 gene expression is characteristic for GCB-like DLBCLs [20]. It is part of the αIgM driven gene module. RGS1 affects chemokine receptor signalling contributing to its desensitization [56]. However, the role of chemokine signalling in lymphomagenesis is not yet fully understood. There are reports suggesting that NHLs express functional chemokine receptors. These, at least in part, dictate tissue localisation and perhaps metastatic potential. However, other reports show that DLBCLs are less sensitive for the CXCR4 ligands CXCL12 and 13 [60,61]. The gene expression changes described above for *CCR7* and *CXCL10* suggest a strong difference of DLBCLs regarding migratory potential and recruitment capacity of cells of the microenvironment but also specific chemokine responsiveness. Because CCR7 and CXCL10 play a pivotal role in the homing of tumour cells as shown by its role in chronic lymphatic leukemia or Hodgkin lymphoma this has to be investigated in the future in more detail. It would be interesting to estimate its role in differences in lymphoma dissemination in relation to the clinical outcome [43,44,62,63].

Strikingly, gene modules of IL21, CD40L or αIgM, even though derived from different data sets, almost perfectly discriminate individual DLBCL. The higher a lymphoma expresses direct αIgM targets the higher it also expresses IL21 or CD40L inducible genes and vice versa. While some explanations can be taken into account, we would favour the following: the aperture of global gene expression changes obtained by computational biology is condensing pathway activities and supports the idea of parallel or equivalent functioning oncogenic activities in individual DLBCLs.

We wanted to further explore potential regulatory mechanisms driving differential expression of gene modules. In order to define potential key molecular determinants, signalling pathways involved in the regulation of a set of genes affected by *in vitro* interventions were specially inhibited using chemical inhibitors.

### B cell receptor regulated genes are dominantly affected by ERK1/2 and PI3K activation

**Figure 6:**
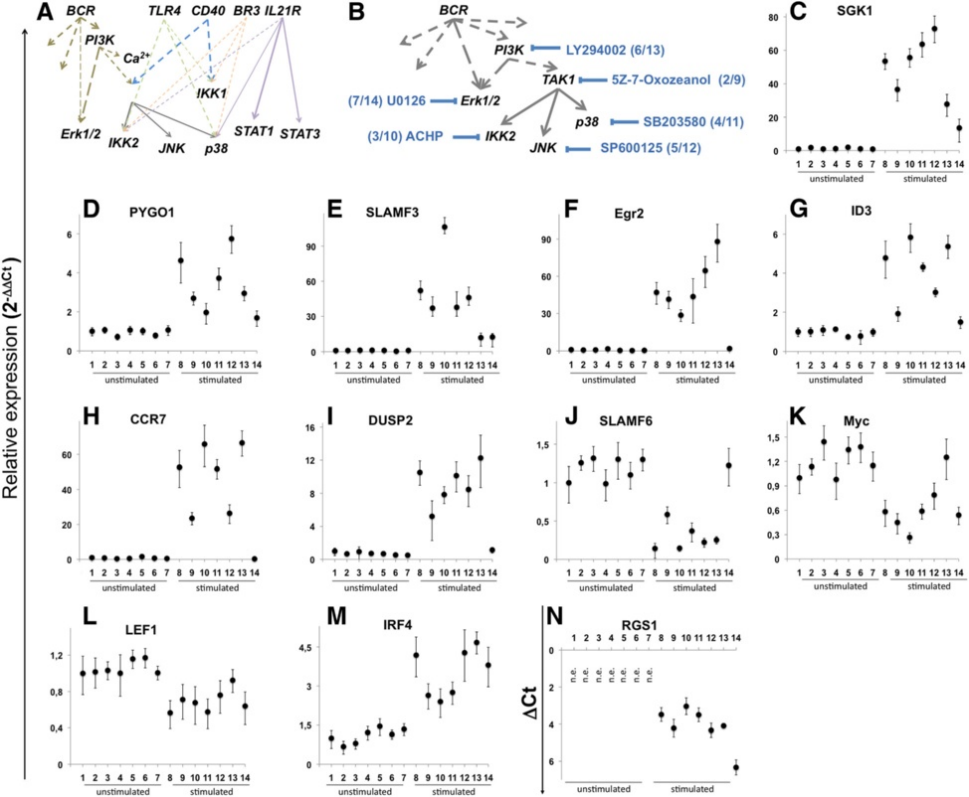
**Pathways involved in the regulation of a selected set of genes in response to αIgM treatment.** (A) Prior knowledge scheme of monitored pathways. Dashed lines implicate the presence of additional signalling molecules interlinking the depicted factors, solid lines reflect a direct link. (B) hierarchical presentation of kinases affected by utilized chemical inhibitors [2]. (C-N) BL2 cell were preincubated for 3 hrs with 2 μM SB203580 (p38), 10 μM SP600125 (JNK), 10 μM U0126 (MAP2K), 100nM 5Z-7-oxozeaenol (TAK), 7 μM ACHP (IKK2) or 10 μM Ly294002 (PI3K) inhibitors and then stimulated by 1.3 μg/ml αIgM F(ab)_2_ fragments for additional 3 hrs in the presence of respective inhibitors. Cells were harvested to isolate RNA for corresponding qRT-PCR. Expression of the following genes is shown: *SGK1, PYGO1, SLAMF3, EGR2, ID3, CCR7, DUSP2, SLAMF6, MYC, LEF1, IRF4 and RGS1.* Results are presented as 2^-ΔΔCT^ values, relative to abl housekeeper expression and compared to the corresponding unstimulated inhibitor treated control. As *RGS1* (N) expression is below detectable levels in unstimulated probes, only ΔCt values relative to stimulated control without inhibitors were compared. One representative experiment out of three or more biological replicates is shown. Cells were treated with DMSO (1/8) 5Z-7-oxozeaenol (2/9), ACHP (3/10), SB203580 (4/11), SP600125 (5/12), Ly294002 (6/13), U0126 (7/14) in the absence (1–7) or presence of αIgM F(ab)_2_ fragments (8–14). n.e. – not expressed. Details on pathway activations as measured by immunoblot, kinase activity and Ca^2+^ influx analysis are summarized in Additional file 24: Figure S3.

Pathway activation by IL21, CD40L, αIgM, BAFF or LPS reflects qualitative and quantitative differences mediated by the activation of the following pathways: Jak/STAT, NF-кB, JNK1/2, p38a, PI3K, Erk1/2 and Ca^2+^ influx by immunoblotting, kinase activity measurement or flow cytometry (Additional file 24: Figure S3). We summarized the pathways activated in our model system in a scheme on Figure 6A. αIgM treatment is associated with Ca^2+^ mobilization. Furthermore Erk1/2, Akt and p38a phosphorylation or enhanced activity of JNK is observed. In addition, the canonical and non-canonical NFкB pathways are activated to some extent as revealed by IкBα degradation and p100 to p52 processing. CD40L activates both canonical and non-canonical NF-кB at the highest level compared to the other stimuli. In addition a p38 phosphorylation and JNK kinase activity is observed comparable to that of αIgM treatment. IL21 stimulation of BL2 cells is mainly associated with STAT1 and STAT3 activation as shown by tyrosine phosphorylation. A slightly reduced expression of IкBα after IL21 treatment is observed, suggesting an activation of the canonical NF-кB. Thus, the perfect discrimination of individual DLBCLs by three different gene modules suggest different magnitudes of simultaneous oncogenic activities mediated by for example Jak/STAT, NF-кB, MAPK (MAPK8/JNK1, MAPK14/p38a, MAP2K1/2), PI3K and Ca^2+^ mediated responses.

Of the stimuli used in this study, αIgM treatment had the strongest effects on gene expression *in vitro* and was capable to activate a wide range of signalling pathways. Therefore, we wanted to further explore pathways involved in the observed differences between individual lymphomas characterized by specific gene module activation. We used chemical kinase inhibitors to identify the pathways involved in the regulation of gene modules in response to stimulation. The utilized inhibitors are summarized in a scheme in Figure 6B showing the hierarchy of kinases in a prior knowledge scheme [2]. The following kinases were considered: MAPK including p38, JNK1/2 or MAP2K1/2 affecting Erk1/2 activation or MAP3K7/TAK1 potentially involved in NF-κB and MAPK signalling. Furthermore, we investigated IKK2 as part of NF-кB signalling and PI3K as it is involved in numerous pathways activated through αIgM, including Akt.

BL2 cell were preincubated for 3 hrs with specific inhibitors and then stimulated by αIgM for additional 3 hrs in the presence of respective inhibitors.

The expression of *SGK1, PYGO1, SLAMF3, DUSP10, EGR2, ID3, CCR7, DUSP2, SLAMF6, BCL6, MYC, LEF1, BCL9, IRF4 and RGS1, DUSP5, SLAMF7* after αIgM treatment was investigated in the absence or presence of the above mentioned kinase inhibitors. Three main groups of regulatory interactions are observed:

Within the first group are genes affected by U0126 interrupting the activity of MAP2K1/2 and Ly294002 inhibiting PI3K. Within this group are *SGK1, PYGO1, SLAMF3/7 and DUSP10 or BCL6,* (Figure 6C, D, E and Additional file 25: Figure S4 A, B, D). This suggests a central role for Erk1/2 and PI3K. Within the second group are genes, dominantly affected by U0126 but not Ly294002. The expression of *EGR2, ID3, CCR7, DUSP2/5 or SLAMF6 and RGS1* is mostly regulated by Erk1/2 (Figure 6F, G, H, I, J, N and Additional file 25: Figure S4 E). In addition, a third group of genes including *MYC, LEF1* as well as *BCL9* is affected by Ly294002 but not U0126 (Figure 6K, L and Additional file 25: Figure S4C).

Interestingly, *IRF4* is the only gene which αIgM affected gene expression is regulated through TAK1/IKK2/p38 (correspondingly 5Z-7-oxozeaenol / ACHP / SB203580) without Erk1/2 or PI3K involvement (Figure 6M).

In addition, αIgM mediated activation of *SGK1* is affected by TAK1 inhibition (Figure 6C), whereas for example *CCR7* activation is regulated through TAK1 and JNK (Figure 6J). Furthermore, for *SGK1, ID3, CCR7* or *SLAMF6,* the effect of the TAK-inhibitor is not accompanied by a comparable IKK2 inhibition. Whereas for *CCR7* and *ID3* the known signalling cascade TAK1-JNK can be proposed, for *SGK1* either a more direct TAK1 effect or a PI3K-TAK1-Erk1/2 cascade has to be taken into account (Figure 6C, G, H). Whereas the expression of *PYGO1* is affected by the well-known TAK1-IKK2 cascade (Figure 6D, I) for *SLAMF6* and *IRF4* also the TAK1-p38 cascade seems to play a role (Figure 6J, M).

αIgM mediated *MYC* inhibition is reversed by the PI3K inhibitor Ly294002. This demonstrates an involvement of PI3K signalling to inhibit aberrant *MYC* expression (Figure 6K). Furthermore, an effect of JNK-, IKK2- or PI3K inhibition on basal expression of *MYC* can be observed. This supports a role of a tonic activation (basal signalling in unstimulated BL2 cells) of PI3K, JNK and IKK2 mediated signalling activity in regulating aberrant “basal” *MYC* expression. Interestingly, a new murine model for lymphomas has been described supporting the view of a synergistic action of c-Myc and PI3K signalling [64]. Furthermore, a tonic BCR signalling and PI(3) kinase activity in Burkitt’s lymphoma has been recently described by Schmitz and co-workers [65]. However, this link between tonic PI3K signalling and *MYC* expression has not been described in this publication. Interestingly, in this study treatment of BL lines with BKM120, a PI(3) kinase inhibitor in clinical trials, or rapamycin, an inhibitor of the mTORC1 complex, was toxic to most BL lines after 4 days. Therefore, their rapamycin signature has to be taken into account for future investigations. Surprisingly, IKK2 inhibition was associated with a much stronger αIgM mediated suppression of *MYC* expression (Figure 6K) [66]. Therefore, we observed a suppressive role of tonic IKK2 activity onto *MYC* expression in BL2 cells. This sheds new light onto the regulation of the aberrant expression of *MYC*. Positive and negative signals from PI3K, MAPK and NF-kB pathways can now be investigated in more detail for example in order to delineate differences between BLs and DLBCLs characterized by a high Myc-index or *MYC* break [67].

A comparable effect of PI3K-inhibition as described for *MYC* is observed also for *BCL6, LEF1* and *BCL9* (Figure 6L and Additional file 24: Figure S3 B, C). However, as for *MYC*, the expression of *BCL6* or *BCL9* is already affected to some extend by Ly294002 in unstimulated BL2 cells. Therefore, it is difficult to interpret these data for *BCL6* and *BCL9* to the end (Additional file 25: Figure S4 B, C). We speculate that combinations of pathways are involved in both basal and αIgM mediated gene expression.

**Figure 7:**
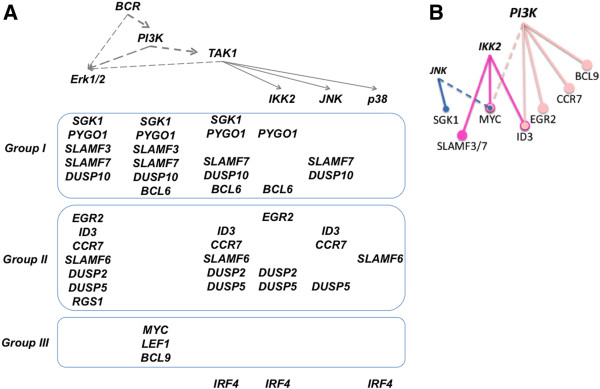
**Schemes summarizing investigated kinase involvement in αIgM mediated gene expression.** αIgM mediated activation / induction of gene expression (**A**) and suppressive effects of pathway elements onto selected genes affecting basal and αIgM mediated gene expression (**B**). Within the scheme (A) genes are listed and sorted according to the groups described within the main text in relation to corresponding kinases. Data for gene expression changes of *BCL6, BCL9, DUSP5, DUSP10* and *SLAMF7* are described within Additional file 25: Figure S4.

In Figure 7A a scheme summarizes the main effects of kinase inhibition observed after αIgM treatment.

As already noted above, in some cases the treatment of cells with inhibitors is associated with an enhanced activation or inhibition of respective genes. For example treatment of cells with Ly294002 led to a stronger activation of *EGR2* or *CCR7* by αIgM treatment (Figure 6E, G). Comparable effects are observed for IKK2 inhibition for *SLAMF3* and *ID3*, for p38 or JNK inhibition analysing *SGK1*, *ID3* or *PYGO1* respectively (Figure 6C, D, E, G). In Figure 7B a respective summary of main αIgM enhancing effects after inhibition of specific kinases is shown, including effects of these inhibitors onto the basal expression levels of analysed genes as for example *MYC* or *BCL9*.

Overall, we found that the expression of most of the analyzed genes affected by αIgM treatment is regulated through Erk1/2 activation accompanied by PI3K, TAK1 and partially to lower extent by IKK2 and JNK.

Erk and PI3K signalling is exclusive to the αIgM gene module (Additional file 24: Figure S3 D, G). These pathways are not affected by the other *in vitro* treatments Activated NF-кB signalling seems to be less important for the αIgM gene module. However, the analysis of CD40 mediated expression of *ICAM1, CD58, SLAMF3* or *CCR7* revealed a strong involvement of NF-кB signalling (data not shown). Our analysis supports the idea that the MAPK/Erk-pathway has a major impact on gene expression in individual DLBCL with a high activation of the αIgM gene module. Therefore, it is reasonable to discuss the use of drugs targeting Erk1/2 for a subgroup of DLBCL characterized by a high activation of the αIgM driven gene module [68]. In a recent study, a molecular interaction of Erk and CHK2 was shown to affect DNA-damage response and apoptosis of DLBCLs [69]. The recently described success of using Syk or Btk inhibitors or even mTOR and PKC inhibitors to treat DLBCL might be explained by the activity of these signalling pathways [70-75]. We are aware of the limitations of chemical kinase inhibitors to analyse pathway elements. However, as comparable compounds are developed for clinical applications, the information drawn from studies integrating *in vitro* stimulations as pathway surrogates with gene expression of individual lymphoma patients will provide comprehensive insights into potential targets for therapy. In the future the utilized *in vitro* stimulations can be used in combination with kinase inhibitors to delineate respective pathway interactions as for example a link between TAK1 and Erk1/2 or the different branches within PI3K signalling by applying also alternative experimental approaches. Furthermore, our data indicate that a global investigation of kinase inhibitors and their combinations would be useful for a better understanding of gene regulation of global gene expression changes and their integration with patient’s data.

## Conclusions

We provide an *in vitro* model system to investigate pathway activations qualitatively and quantitatively. B cell specific stimuli are used to identify gene expression changes allowing to “switch“ gene expression from one steady state level characteristic for BL towards that of DLBCLs. We defined the extent to which specific signalling pathways are responsible for differences in gene expression that distinguish individual DLBCL. Gene modules of IL21, CD40L or αIgM discriminate individual DLBCL, from each other, even though derived from different data sets. The greater an individual lymphoma expresses αIgM target genes, the greater it will also express IL21 or CD40L regulated genes.

We have shown that mitogen activated protein kinase- and phosphoinositide 3 kinase-signaling are an important part of pathway networks describing differences in gene expression that distinguish individual DLBCL. This observation supports recent findings about the role of tonic and/or chronic active MAPK signalling in individual lymphoma and might therefore constitute a promising target for future therapy approaches. Although the discrimination of individual DLBCL by three different gene modules suggest different magnitudes of parallel or equivalent oncogenic activities mediated by Jak/STAT, NF-кB, MAPK. Therefore, transformed human germinal centre B cells can be used to test new compounds and their influence on the respective pathways in DLBCLs. A useful tool to test for individual treatment strategies is offered, which is independent from heterogeneous lymphoma associated mutations know from DLBCLs.

## Materials and methods

### Cell culture and stimulation

BL2 cells were cultivated as described previously at cell densities between 2 × 10^5^ and 1 × 10^6^ cells/ml [63]. For stimulation studies, cells were cultured in cell culture medium supplemented with 10 mM HEPES at 1 × 10^6^ cells/ml and incubated with indicated reagents for up to 9 hrs. To crosslink the BCR, BL2 cells were cultured in the presence of 1.3 μg/ml goat αIgM F(ab)_2_ fragments (Jackson Immunity). Recombinant human sCD40L (AutogenBioclear), human BAFF (R&D Systems) and recombinant human IL21 (Peprotech) were used at a concentration of 200 ng/ml, 100 ng/ml and 100 ng/ml respectively. LPS (E. coli strain 055:B5, Sigma) was added to the cells at a concentration of 1 μM.

Cells were harvested using corresponding inhibitors of phosphatases and proteases and RNA was isolated using the RNeasy Plus Mini Kit (Qiagen).

Immunoblot, Calcium Measurement, JNK Immunocomplex kinase assays and qRT-PCR analysis are summarized within supplemental Material and Methods.

### Gene expression analysis

For gene expression analysis RNA was isolated with RNeasy Plus Mini Kit (Qiagen) according to the manufacturer’s instructions. For real time PCR analysis RNA was reverse transcribed using SuperScript II Reverse Transcriptase (Invitrogen) and random hexamer primers (IBA BioTAGnology). cDNA samples were further analysed by SYBR Green-based real-time PCR using the 7900HT Fast Real-Time PCR System (Applied Biosystems) (additional details for used primers within the Additional file 26: Table S17). For whole genome micorarrays RNA was labelled for microarray hybridization using Affymetrix GeneChip® IVT Labelling Kit (Affymetrix). Fragmentation and hybridization of labelled anti sense RNA on Human Genome U133A 2.0 plus Arrays (Affymetrix) was performed according to manufacturer’s recommendations by the Kompetenzzentrum für Fluoreszente Bioanalytik. Rawdata have been uploaded to GEO and can be assessed using GSE42660. Gene expression values were obtained by first correcting for the background and normalizing on probe level using the variance stabilization method by Huber and colleagues [55]. The normalized probe intensities were summarized into gene expression levels by using an additive model fitted by the median polish procedure [76,77]. If there was more than one probeset per gene, we kept the probeset best responding. This was done by looking at the fold changes between control and stimulation, the probeset with the highest fold change was kept. Additional details for Biostatistics are summarized within supplemental Material and Methods. Ethical approval for gene expression studies on human lymphoma material was granted and described in detail by Hummel and colleagues [20] as well as Dave and colleagues [21]. These studies were conducted in compliance with the Declaration of Helsinki.

## Competing interests

The authors declare that they have no competing interests.

## Authors’ contributions

SA, VM, KA, EM performed experiments and wrote the manuscript. VBF, HE, UA, MK, LD performed experiments. MK, performed statistics and wrote the manuscript. HM, TL wrote the manuscript. KD. design of the study, wrote the manuscript and finally approved. All authors read and approved the final manuscript.

## Supplementary Material

Additional file 1 Supplementary Materials and Methods. Click here for file

Additional file 2 **Table S1.** Genes affected by α-IgM treatment. Click here for file

Additional file 3 **Table S2.** Genes affected by IL21 treatment. Click here for file

Additional file 4 **Table S3.** Genes affected by CD40L treatment. Click here for file

Additional file 5 **Table S4.** Genes affected by BAFF treatment. Click here for file

Additional file 6 **Table S5.** Genes affected by LPS treatment. Click here for file

Additional file 7 **Figure S1.** Global gene expression changes of CD40L stimulation are highly comparable in distinct Burkitt Lymphoma cell lines (Ramos and BL2). Click here for file

Additional file 8 **Supplemental 1.** A selection of microarray data providing insight into gene expression changes affected by CD40L, B cell receptor activation, for BAFF, LPS or IL21. Click here for file

Additional file 9 **Supplemental 2.** Geneset enrichment Analysis identifying enriched pathways in differentially expressed genes. Click here for file

Additional file 10 **Table S6.** Differentially expressed genes overlapping between CD40L and IL21 stimulation. Click here for file

Additional file 11 **Table S7.** Differentially expressed genes overlapping between α-IgM and IL21 stimulation. Click here for file

Additional file 12 **Table S8.** Differentially expressed genes overlapping between LPS and IL21 stimulation. Click here for file

Additional file 13 **Table S9.** Differentially expressed genes overlapping between BAFF and IL21 stimulation. Click here for file

Additional file 14 **Table S10.** Differentially expressed genes overlapping between CD40L and α-IgM stimulation. Click here for file

Additional file 15 **Table S11.** Differentially expressed genes overlapping between CD40L and LPS stimulation. Click here for file

Additional file 16 **Table S12.** Differentially expressed genes overlapping between CD40L and BAFF stimulation. Click here for file

Additional file 17 **Table S13.** Differentially expressed genes overlapping between LPS and α-IgM stimulation. Click here for file

Additional file 18 **Table S14.** Differentially expressed genes overlapping between BAFF and α-IgM stimulation. Click here for file

Additional file 19 **Table S15.** Differentially expressed genes overlapping between LPS and BAFF stimulation. Click here for file

Additional file 20 **Supplemental 3.** Geneset enrichment Analysis identifying enriched pathways in differentially expressed genes overlapping between stimulations. Click here for file

Additional file 21 **Table S16.** Genes differentially expressed in response to only one specific stimulation (unique). Click here for file

Additional file 22 **Supplemental 4.** Geneset enrichment Analysis identifying enriched pathways in differentially expressed genes unique for each specific stimulation. Click here for file

Additional file 23 **Figure S2.** Lymphoma cases showing a high expression of α-IgM responsive genes as well characterized by a high expression of the genes affected by the other analysed interventions. Click here for file

Additional file 24 **Figure S3.** Qualitative and quantitative differences in pathway activation by IL21, CD40L, αIgM, BAFF or LPS in human transformed GC B cells *in vitro*. Click here for file

Additional file 25 **Figure S4.** Pathways involved in the regulation of a selected set of induced genes in response to αIgM treatment. Click here for file

Additional file 26 **Table S17.** Utilized Oligonucleotides. Click here for file
