# Supplementary material for: Global gene expression changes of in vitro stimulated human transformed germinal centre B cells as surrogate for oncogenic pathway activation in individual aggressive B cell lymphomas
Source: Cell Commun Signal. 2012 Dec 20;10:43. doi: 10.1186/1478-811X-10-43 (PMC3566944; doi:10.1186/1478-811X-10-43)
Supplement: Additional file 1 — Supplementary Materials and Methods. [file 1478-811X-10-43-S1.docx]

*Western blot analysis*

Cells were analysed for protein expression by SDS polyacrylamide gel electrophoresis and Western blot analysis using the following antibodies: mouse monoclonal anti α-tubulin ((#05-829) Upstate/Millipore), rabbit α-p-AKT (Ser473) ((#9271) Cell Signalling/ New England Biolabs), rabbit α- pan AKT ((#100-401-401) Rockland via BioMol), rabbit α-pan AKT (#9272), Cell Signalling/ New England Biolabs), rabbit α-p-p38 ((#9211) Cell Signalling/ New England Biolabs), rabbit α-p38 ((#9212) Cell Signalling/ New England Biolabs), rabbit α-p-p42/44 ((#4377) Cell Signalling/ New England Biolabs), rabbit α-p-42/44 ((#4695) Cell Signalling/ New England Biolabs), rabbit α-p-STAT1 (Tyr701) ((#9171) Cell Signalling/ New England Biolabs), rabbit α-STAT1 ((#9172), Cell Signalling/ New England Biolabs), rabbit α- p-STAT3 (Ser727) ((#9134) Cell Signalling/ New England Biolabs), rabbit α- STAT3 ((#9132), Cell Signalling/ New England Biolabs), rabbit α- p-STAT5 (#9351), Cell Signalling/ New England Biolabs), mouse α- STAT5 ((#610191) BD/Pharmigen), rabbit α- p-STAT6 (Y641) ((#9361) Cell Signalling/ New England Biolabs), mouse α-STAT6 (S-20) X ((#611291), BD/Pharmigen), rabbit α- p100/p52 ((#4882), Cell Signalling/ New England Biolabs), rabbit α-IkappaBalpha (44D4) ((#4812), Cell Signalling/ New England Biolabs), α-mouse HRP polyclonal goat ((D1609) Santa Cruz) and α-abbit HRP polyclonal goat ((E1710) Santa Cruz).

For the gene expression analyses in combination with pathway inhibition 1x10^6^ cells/ml were treated for 3h prior to BCR activation with 100nM 5Z-7-oxozeaenol (TAK1-inhibitor), 7µM 2-Amino-6-(2-(cyclopropylmethoxy)-6-hydroxyphenyl)-4-(4-piperidinyl)-3-pyridinecarbonitrile (ACHP) (IKK2-inhibitor), 10µM Ly294002 (PI3K inhibitor), 2µM SB203580 (p38/MAPK14 inhibitor), 10µM SP600125 (JNK inhibitor) and 10µM U0126 (MAP2K1/2 inhibitor). All inhibitors are purchased from Merck-Calbiochem. The stimulation with αIgM was performed for another 3h [1-6].

*qRT-PCR*

qRT-PCR was perfomed using SYBR green. ∆Ct values were normalised to ß2m and abl expression and ∆∆Ct values were calculated. Oligonucleotides used are summerized in **suppl. Table 17**.

*Calcium Measurement:*

The Ca^2+^ mobilization in BL cells was measured using the Ca^2+^-sensitive fluorophore Indo-1 (Indo-1-AM) and flow cytometry as described in [7]. Briefly, 1x10^6^ cells were harvested at 300xg, 4°C, 5 min. The cells were resuspended in 700µl RPMI containing 5% FCS. The cells were loaded with Indo-1 for 25 min at 30°C and diluted with 700µl of the corresponding prewarmed medium containing 10% FCS. Cells were incubated for 10 min at 37°C and washed twice with Ca^2+^ containing Krebs-Ringer solution. Cells were resuspended in Ca^2+^-containing Krebs-Ringer solution. The ratio of 355 nm-induced fluorescence signals at 405nm and 530nm (Indo-violet/Indo-blue) was measured using a LSR II (Becton Dickinson). After 30s, stimulation was performed with either 1.3µg/ml αIgM F(ab’)2 fragment or 200ng/ml sCD40L, 100ng/ml BAFF, 100ng/ml IL21 or 1µM LPS. The Ca^2+^-mobilization profiles were analyzed using FlowJo software.

*JNK Immunocomplex kinase assays*

Cells were treated as described in the figure legends and in cell culture methods. Immunoprecipitations were performed as described [8] using the monoclonal α-hemagglutinin antibody 12CA5 (Boehringer) or the rabbit anti-JNK1 antibody C-17 (Santa Cruz Biotech.), immobilized to protein G–Sepharose beads (Pharmacia), to immunoprecipitate HA-JNK1 or endogenous JNK1, respectively. In vitro immunocomplex kinase assays with the immunoprecipitated kinases were performed as described [72] using glutathione-S-transferase (GST)-tagged c-Jun (purified from *E.coli*) as substrates for JNK1. As indicated, kinase reactions or total cell lysates were separated by SDS–PAGE and blotted onto Hybond-C membranes (Amersham). Kinase reactions were analysed by autoradiography and phosphoimager scanning. The following antibodies were used for immunoblotting: the rabbit α-JNK1 antibody (C-17, Santa Cruz Biotech).

*Bioinformatics*

Differentially expressed genes between perturbed and control cell lines were identified using linear models as implemented in the bioconductor package LIMMA [9]. The experimental batches were explicitly modelled. False discovery rates for lists of differentially expressed genes were calculated according to Benjamini and Hochberg [10]. Genes were ranked according to their p-value for differential expression from the microarray experiments. Similarity in the rankings of lists of differentially expressed genes between perturbations were assessed using the ordered list algorithm [11]. The expression levels of a list of 100 genes with a FDR < 0.01 were examined in clinical lymphoma samples [12, 13]. Of these 100 genes, 68 genes were present on the Affymetrix HG-U133A gene chip used for profiling the lymphomas. Their joint expression was condensed using a standard additive model fitted by Tuckey’s median polish procedure. The primary data are available from GEO (http://www.ncbi.nlm.nih.gov/geo/) under series accession no. GSEXX, GSEXX, GSEXX, GSEXX. Raw data for gene expression changes for LPS stimulated BL2 cells have been used previously but not described in experimental details [14].

1. Vockerodt M, Pinkert D, Smola-Hess S, Michels A, Ransohoff RM, Tesch H, Kube D: **The Epstein-Barr virus oncoprotein latent membrane protein 1 induces expression of the chemokine IP-10: importance of mRNA half-life regulation.** *Int J Cancer* 2005, **114:**598-605.

2. Kutz H, Reisbach G, Schultheiss U, Kieser A: **The c-Jun N-terminal kinase pathway is critical for cell transformation by the latent membrane protein 1 of Epstein-Barr virus.** *Virology* 2008, **371:**246-256.

3. Ear T, Fortin CF, Simard FA, McDonald PP: **Constitutive association of TGF-beta-activated kinase 1 with the IkappaB kinase complex in the nucleus and cytoplasm of human neutrophils and its impact on downstream processes.** *J Immunol* 2010, **184:**3897-3906.

4. de Jong SJ, Albrecht JC, Schmidt M, Muller-Fleckenstein I, Biesinger B: **Activation of noncanonical NF-kappaB signaling by the oncoprotein Tio.** *J Biol Chem* 2010, **285:**16495-16503.

5. Adachi S, Kuwata T, Miyaike M, Iwata M: **Induction of CCR7 expression in thymocytes requires both ERK signal and Ca(2+) signal.** *Biochem Biophys Res Commun* 2001, **288:**1188-1193.

6. Uddin S, Hussain AR, Siraj AK, Manogaran PS, Al-Jomah NA, Moorji A, Atizado V, Al-Dayel F, Belgaumi A, El-Solh H, et al: **Role of phosphatidylinositol 3'-kinase/AKT pathway in diffuse large B-cell lymphoma survival.** *Blood* 2006, **108:**4178-4186.

7. Stork B, Engelke M, Frey J, Horejsi V, Hamm-Baarke A, Schraven B, Kurosaki T, Wienands J: **Grb2 and the non-T cell activation linker NTAL constitute a Ca(2+)-regulating signal circuit in B lymphocytes.** *Immunity* 2004, **21:**681-691.

8. Kieser A, Kilger E, Gires O, Ueffing M, Kolch W, Hammerschmidt W: **Epstein-Barr virus latent membrane protein-1 triggers AP-1 activity via the c-Jun N-terminal kinase cascade.** *EMBO J* 1997, **16:**6478-6485.

9. Smyth GK, Michaud J, Scott HS: **Use of within-array replicate spots for assessing differential expression in microarray experiments.** *Bioinformatics* 2005, **21:**2067-2075.

10. Benjamini Y, Hochberg Y: **Controlling the false discovery rate: a practical and powerful approach to multiple testing.** *J R Statist Soc B* 1995, **57:**289-300.

11. Lottaz C, Yang X, Scheid S, Spang R: **OrderedList--a bioconductor package for detecting similarity in ordered gene lists.** *Bioinformatics* 2006, **22:**2315-2316.

12. Dave SS, Fu K, Wright GW, Lam LT, Kluin P, Boerma EJ, Greiner TC, Weisenburger DD, Rosenwald A, Ott G, et al: **Molecular diagnosis of Burkitt's lymphoma.** *N Engl J Med* 2006, **354:**2431-2442.

13. Hummel M, Bentink S, Berger H, Klapper W, Wessendorf S, Barth TF, Bernd HW, Cogliatti SB, Dierlamm J, Feller AC, et al: **A biologic definition of Burkitt's lymphoma from transcriptional and genomic profiling.** *N Engl J Med* 2006, **354:**2419-2430.

14. Maneck M, Schrader A, Kube D, Spang R: **Genomic data integration using guided clustering.** *Bioinformatics* 2011.
