# Supplementary material for: Global gene expression changes of in vitro stimulated human transformed germinal centre B cells as surrogate for oncogenic pathway activation in individual aggressive B cell lymphomas
Source: Cell Commun Signal. 2012 Dec 20;10:43. doi: 10.1186/1478-811X-10-43 (PMC3566944; doi:10.1186/1478-811X-10-43)
Supplement: Additional file 7 — Figure S1. Global gene expression changes of CD40L stimulation are highly comparable in distinct Burkitt Lymphoma cell lines (Ramos and BL2). [file 1478-811X-10-43-S7.docx]

**Supplementary Figure 1: Global gene expression changes of CD40L stimulation are highly comparable in distinct Burkitt Lymphoma cell lines (Ramos and BL2).** Geneset Enrichment Analyses were utilized to investigate the similarities of the CD40L effects on gene expression profiles of Ramos and BL2 cells. We found that the CD40L affected genes identified by Basso et al. in the BL cell line Ramos (geneset size 121 genes) are highly enriched in the CD40L responsive genes identified in our BL2 cell system. This is visualized by the sharp increase of the Enrichment Score in high positions of the ranked list of genes (highlighted in magenta).
