# Supplementary material for: Global gene expression changes of in vitro stimulated human transformed germinal centre B cells as surrogate for oncogenic pathway activation in individual aggressive B cell lymphomas
Source: Cell Commun Signal. 2012 Dec 20;10:43. doi: 10.1186/1478-811X-10-43 (PMC3566944; doi:10.1186/1478-811X-10-43)
Supplement: Additional file 8 — Supplemental 1. A selection of microarray data providing insight into gene expression changes affected by CD40L, B cell receptor activation, for BAFF, LPS or IL21. [file 1478-811X-10-43-S8.docx]

A selection of microarray data providing insight into gene expression changes affected by CD40L, B cell receptor activation, for BAFF, LPS or IL21.

1. Basso K, Klein U, Niu H, Stolovitzky GA, Tu Y, Califano A *et al.* Tracking CD40 signaling during germinal center development. *Blood* 2004; **104**(13)**:** 4088-96.

2. Dadgostar H, Zarnegar B, Hoffmann A, Qin XF, Truong U, Rao G *et al.* Cooperation of multiple signaling pathways in CD40-regulated gene expression in B lymphocytes. *Proc Natl Acad Sci U S A* 2002; **99**(3)**:** 1497-502.

3. Zhu X, Hart R, Chang MS, Kim JW, Lee SY, Cao YA *et al.* Analysis of the major patterns of B cell gene expression changes in response to short-term stimulation with 33 single ligands. *J Immunol* 2004; **173**(12)**:** 7141-9.

4. Schuh W, Meister S, Herrmann K, Bradl H, Jack HM. Transcriptome analysis in primary B lymphoid precursors following induction of the pre-B cell receptor. *Mol Immunol* 2008; **45**(2)**:** 362-75.

5. Aflakian N, Ravichandran S, Jamal MS, Jarvenpaa H, Lahesmaa R, Rao KV. Integration of signals from the B-cell antigen receptor and the IL-4 receptor leads to a cooperative shift in the cellular response axis. *Mol Biosyst* 2009; **5**(12)**:** 1661-71.

6. Jamal MS, Ravichandran S, Jailkhani N, Chatterjee S, Dua R, Rao KV. Defining the antigen receptor-dependent regulatory network that induces arrest of cycling immature B-lymphocytes. *BMC Syst Biol* 2010; **4:** 169.

7. Natarajan M, Lin KM, Hsueh RC, Sternweis PC, Ranganathan R. A global analysis of cross-talk in a mammalian cellular signalling network. *Nat Cell Biol* 2006; **8**(6)**:** 571-80.

8. Jain S, Chodisetti SB, Agrewala JN. CD40 signaling synergizes with TLR-2 in the BCR independent activation of resting B cells. *PLoS One* 2011; **6**(6)**:** e20651.

9. Gricks CS, Zahrieh D, Zauls AJ, Gorgun G, Drandi D, Mauerer K *et al.* Differential regulation of gene expression following CD40 activation of leukemic compared to healthy B cells. *Blood* 2004; **104**(13)**:** 4002-9.

10. Sarosiek KA, Malumbres R, Nechushtan H, Gentles AJ, Avisar E, Lossos IS. Novel IL-21 signaling pathway up-regulates c-Myc and induces apoptosis of diffuse large B-cell lymphomas. *Blood* 2010; **115**(3)**:** 570-80.

11. Tai YT, Li XF, Breitkreutz I, Song W, Neri P, Catley L *et al.* Role of B-cell-activating factor in adhesion and growth of human multiple myeloma cells in the bone marrow microenvironment. *Cancer Res* 2006; **66**(13)**:** 6675-82.

12. Zarnegar B, He JQ, Oganesyan G, Hoffmann A, Baltimore D, Cheng G. Unique CD40-mediated biological program in B cell activation requires both type 1 and type 2 NF-kappaB activation pathways. *Proc Natl Acad Sci U S A* 2004; **101**(21)**:** 8108-13.

13. Lee JA, Sinkovits RS, Mock D, Rab EL, Cai J, Yang P *et al.* Components of the antigen processing and presentation pathway revealed by gene expression microarray analysis following B cell antigen receptor (BCR) stimulation. *BMC Bioinformatics* 2006; **7:** 237.

14. Murn J, Mlinaric-Rascan I, Vaigot P, Alibert O, Frouin V, Gidrol X. A Myc-regulated transcriptional network controls B-cell fate in response to BCR triggering. *BMC Genomics* 2009; **10:** 323.

15. Shaffer AL, Wright G, Yang L, Powell J, Ngo V, Lamy L *et al.* A library of gene expression signatures to illuminate normal and pathological lymphoid biology. *Immunol Rev* 2006; **210:** 67-85.
