# Supplementary material for: Global gene expression changes of in vitro stimulated human transformed germinal centre B cells as surrogate for oncogenic pathway activation in individual aggressive B cell lymphomas
Source: Cell Commun Signal. 2012 Dec 20;10:43. doi: 10.1186/1478-811X-10-43 (PMC3566944; doi:10.1186/1478-811X-10-43)
Supplement: Additional file 9 — Supplemental 2. Geneset enrichment Analysis identifying enriched pathways in differentially expressed genes. [file 1478-811X-10-43-S9.zip › supplementalFile2_GO_AnalysenLIMMA/BAFF.1_up.html]

- 265 unique Entrez Gene IDs considered
- on chip with 22283 probesets

- Molecular function
- Biological process
- Cellular component
- Pathways (KEGG)

### Molecular Function

- 10870 Entrez Gene IDs have annotations in category 'MF'
- 245 of these are in the above list
- upreg means upregulated in group BAFF\_regulated.1 and downreg means downregulated in group BAFF\_regulated.1

|  |  |  |  |  |  |  |
| --- | --- | --- | --- | --- | --- | --- |
| **GO ID** | **GO Term** | **upreg. p-value** | **upreg. int. Count** | **downreg. p-value** | **downreg. int. Count** | **GO Count** |
| GO:0003676 | nucleic acid binding | 3e-13 | 94 | 0.570 | 1 | 2069 |
| GO:0003723 | RNA binding | 8e-11 | 40 | 0.195 | 1 | 574 |
| GO:0000166 | nucleotide binding | 2e-10 | 79 | 0.510 | 1 | 1776 |
| GO:0004386 | helicase activity | 3e-07 | 14 | 1.000 | 0 | 116 |
| GO:0005524 | ATP binding | 4e-07 | 53 | 1.000 | 0 | 1183 |
| GO:0032559 | adenyl ribonucleotide binding | 6e-07 | 53 | 1.000 | 0 | 1202 |
| GO:0016887 | ATPase activity | 1e-06 | 21 | 1.000 | 0 | 284 |
| GO:0030554 | adenyl nucleotide binding | 1e-06 | 54 | 0.391 | 1 | 1268 |
| GO:0001882 | nucleoside binding | 2e-06 | 55 | 0.402 | 1 | 1311 |
| GO:0003678 | DNA helicase activity | 2e-06 | 8 | 1.000 | 0 | 39 |
| GO:0008094 | DNA-dependent ATPase activity | 3e-06 | 9 | 1.000 | 0 | 55 |
| GO:0001883 | purine nucleoside binding | 3e-06 | 54 | 0.399 | 1 | 1301 |
| GO:0016818 | hydrolase activity, acting on acid anhydrides, in phosphorus-containing anhydrides | 5e-06 | 32 | 1.000 | 0 | 611 |
| GO:0016817 | hydrolase activity, acting on acid anhydrides | 5e-06 | 32 | 1.000 | 0 | 613 |
| GO:0032553 | ribonucleotide binding | 1e-05 | 57 | 1.000 | 0 | 1460 |
| GO:0032555 | purine ribonucleotide binding | 1e-05 | 57 | 1.000 | 0 | 1460 |
| GO:0016462 | pyrophosphatase activity | 1e-05 | 31 | 1.000 | 0 | 608 |
| GO:0017111 | nucleoside-triphosphatase activity | 1e-05 | 30 | 1.000 | 0 | 584 |
| GO:0008026 | ATP-dependent helicase activity | 2e-05 | 10 | 1.000 | 0 | 84 |
| GO:0070035 | purine NTP-dependent helicase activity | 2e-05 | 10 | 1.000 | 0 | 84 |
| GO:0017076 | purine nucleotide binding | 2e-05 | 58 | 0.454 | 1 | 1528 |
| GO:0005515 | protein binding | 4e-05 | 175 | 0.825 | 2 | 6561 |
| GO:0003677 | DNA binding | 4e-05 | 53 | 0.421 | 1 | 1386 |
| GO:0004527 | exonuclease activity | 4e-05 | 7 | 1.000 | 0 | 43 |
| GO:0005488 | binding | 5e-05 | 223 | 0.877 | 3 | 9129 |
| GO:0043138 | 3'-5' DNA helicase activity | 1e-04 | 3 | 1.000 | 0 | 5 |
| GO:0003697 | single-stranded DNA binding | 2e-04 | 7 | 1.000 | 0 | 54 |
| GO:0004003 | ATP-dependent DNA helicase activity | 2e-04 | 5 | 1.000 | 0 | 25 |
| GO:0016018 | cyclosporin A binding | 4e-04 | 3 | 1.000 | 0 | 7 |
| GO:0032181 | dinucleotide repeat insertion binding | 5e-04 | 2 | 1.000 | 0 | 2 |
| GO:0042623 | ATPase activity, coupled | 7e-04 | 14 | 1.000 | 0 | 232 |
| GO:0042393 | histone binding | 7e-04 | 6 | 1.000 | 0 | 49 |
| GO:0008022 | protein C-terminus binding | 9e-04 | 10 | 1.000 | 0 | 136 |
| GO:0043566 | structure-specific DNA binding | 9e-04 | 10 | 1.000 | 0 | 136 |
| GO:0016779 | nucleotidyltransferase activity | 0.001 | 8 | 1.000 | 0 | 94 |
| GO:0035064 | methylated histone residue binding | 0.001 | 3 | 1.000 | 0 | 10 |
| GO:0008408 | 3'-5' exonuclease activity | 0.001 | 4 | 1.000 | 0 | 22 |
| GO:0004518 | nuclease activity | 0.001 | 9 | 1.000 | 0 | 119 |
| GO:0003918 | DNA topoisomerase (ATP-hydrolyzing) activity | 0.001 | 2 | 1.000 | 0 | 3 |
| GO:0032138 | single base insertion or deletion binding | 0.001 | 2 | 1.000 | 0 | 3 |
| GO:0032139 | dinucleotide insertion or deletion binding | 0.001 | 2 | 1.000 | 0 | 3 |
| GO:0032142 | single guanine insertion binding | 0.001 | 2 | 1.000 | 0 | 3 |
| GO:0032356 | oxidized DNA binding | 0.001 | 2 | 1.000 | 0 | 3 |
| GO:0032357 | oxidized purine DNA binding | 0.001 | 2 | 1.000 | 0 | 3 |
| GO:0043515 | kinetochore binding | 0.001 | 2 | 1.000 | 0 | 3 |
| GO:0003755 | peptidyl-prolyl cis-trans isomerase activity | 0.003 | 4 | 1.000 | 0 | 27 |
| GO:0000739 | DNA strand annealing activity | 0.003 | 2 | 1.000 | 0 | 4 |
| GO:0003916 | DNA topoisomerase activity | 0.003 | 2 | 1.000 | 0 | 4 |
| GO:0032135 | DNA insertion or deletion binding | 0.003 | 2 | 1.000 | 0 | 4 |
| GO:0032137 | guanine/thymine mispair binding | 0.003 | 2 | 1.000 | 0 | 4 |
| GO:0043140 | ATP-dependent 3'-5' DNA helicase activity | 0.003 | 2 | 1.000 | 0 | 4 |
| GO:0016859 | cis-trans isomerase activity | 0.004 | 4 | 1.000 | 0 | 29 |
| GO:0003690 | double-stranded DNA binding | 0.004 | 7 | 1.000 | 0 | 90 |
| GO:0048256 | flap endonuclease activity | 0.005 | 2 | 1.000 | 0 | 5 |
| GO:0016796 | exonuclease activity, active with either ribo- or deoxyribonucleic acids and producing 5'-phosphomonoesters | 0.006 | 3 | 1.000 | 0 | 17 |
| GO:0003899 | DNA-directed RNA polymerase activity | 0.006 | 4 | 1.000 | 0 | 33 |
| GO:0034062 | RNA polymerase activity | 0.006 | 4 | 1.000 | 0 | 33 |
| GO:0003777 | microtubule motor activity | 0.006 | 5 | 1.000 | 0 | 53 |
| GO:0019237 | centromeric DNA binding | 0.007 | 2 | 1.000 | 0 | 6 |
| GO:0032405 | MutLalpha complex binding | 0.007 | 2 | 1.000 | 0 | 6 |
| GO:0070087 | chromo shadow domain binding | 0.007 | 2 | 1.000 | 0 | 6 |
| GO:0070628 | proteasome binding | 0.007 | 2 | 1.000 | 0 | 6 |
| GO:0016853 | isomerase activity | 0.007 | 7 | 1.000 | 0 | 101 |
| GO:0004402 | histone acetyltransferase activity | 0.008 | 4 | 1.000 | 0 | 36 |
| GO:0004468 | lysine N-acetyltransferase activity | 0.008 | 4 | 1.000 | 0 | 36 |
| GO:0016455 | RNA polymerase II transcription mediator activity | 0.008 | 3 | 1.000 | 0 | 19 |
| GO:0003682 | chromatin binding | 0.009 | 9 | 0.058 | 1 | 160 |
| GO:0004520 | endodeoxyribonuclease activity | 0.009 | 3 | 1.000 | 0 | 20 |
| GO:0032404 | mismatch repair complex binding | 0.010 | 2 | 1.000 | 0 | 7 |
| GO:0034450 | ubiquitin-ubiquitin ligase activity | 0.010 | 2 | 1.000 | 0 | 7 |
| GO:0045502 | dynein binding | 0.010 | 2 | 1.000 | 0 | 7 |
| GO:0060590 | ATPase regulator activity | 0.010 | 2 | 1.000 | 0 | 7 |

### Biological Process

- 10392 Entrez Gene IDs have annotations in category 'BP'
- 228 of these are in the above list
- upreg means upregulated in group BAFF\_regulated.1 and downreg means downregulated in group BAFF\_regulated.1

|  |  |  |  |  |  |  |
| --- | --- | --- | --- | --- | --- | --- |
| **GO ID** | **GO Term** | **upreg. p-value** | **upreg. int. Count** | **downreg. p-value** | **downreg. int. Count** | **GO Count** |
| GO:0090304 | nucleic acid metabolic process | <2e-16 | 120 | 0.710 | 1 | 2768 |
| GO:0006139 | nucleobase, nucleoside, nucleotide and nucleic acid metabolic process | 3e-16 | 128 | 0.774 | 1 | 3229 |
| GO:0034641 | cellular nitrogen compound metabolic process | 8e-15 | 131 | 0.804 | 1 | 3479 |
| GO:0044260 | cellular macromolecule metabolic process | 3e-14 | 149 | 0.884 | 1 | 4325 |
| GO:0006396 | RNA processing | 7e-14 | 40 | 1.000 | 0 | 475 |
| GO:0006807 | nitrogen compound metabolic process | 8e-14 | 131 | 0.815 | 1 | 3573 |
| GO:0008380 | RNA splicing | 1e-13 | 29 | 1.000 | 0 | 251 |
| GO:0006996 | organelle organization | 3e-12 | 65 | 0.398 | 1 | 1238 |
| GO:0022402 | cell cycle process | 2e-11 | 40 | 1.000 | 0 | 566 |
| GO:0016071 | mRNA metabolic process | 2e-11 | 29 | 1.000 | 0 | 310 |
| GO:0051276 | chromosome organization | 3e-11 | 35 | 1.000 | 0 | 448 |
| GO:0043170 | macromolecule metabolic process | 5e-11 | 151 | 0.915 | 1 | 4772 |
| GO:0048285 | organelle fission | 5e-11 | 25 | 1.000 | 0 | 239 |
| GO:0007049 | cell cycle | 6e-11 | 49 | 1.000 | 0 | 837 |
| GO:0000279 | M phase | 8e-11 | 29 | 1.000 | 0 | 328 |
| GO:0006260 | DNA replication | 8e-11 | 23 | 1.000 | 0 | 206 |
| GO:0000280 | nuclear division | 1e-10 | 24 | 1.000 | 0 | 230 |
| GO:0007067 | mitosis | 1e-10 | 24 | 1.000 | 0 | 230 |
| GO:0051301 | cell division | 2e-10 | 27 | 1.000 | 0 | 294 |
| GO:0022403 | cell cycle phase | 2e-10 | 33 | 1.000 | 0 | 432 |
| GO:0006397 | mRNA processing | 2e-10 | 25 | 1.000 | 0 | 257 |
| GO:0000087 | M phase of mitotic cell cycle | 3e-10 | 24 | 1.000 | 0 | 238 |
| GO:0000278 | mitotic cell cycle | 5e-10 | 32 | 1.000 | 0 | 423 |
| GO:0010467 | gene expression | 5e-10 | 104 | 0.721 | 1 | 2838 |
| GO:0007059 | chromosome segregation | 8e-10 | 14 | 1.000 | 0 | 77 |
| GO:0044237 | cellular metabolic process | 2e-09 | 166 | 0.759 | 2 | 5718 |
| GO:0016070 | RNA metabolic process | 2e-09 | 79 | 0.562 | 1 | 1940 |
| GO:0006281 | DNA repair | 5e-09 | 23 | 1.000 | 0 | 254 |
| GO:0006259 | DNA metabolic process | 6e-09 | 33 | 1.000 | 0 | 494 |
| GO:0006974 | response to DNA damage stimulus | 3e-08 | 26 | 1.000 | 0 | 347 |
| GO:0071103 | DNA conformation change | 4e-08 | 14 | 1.000 | 0 | 104 |
| GO:0044238 | primary metabolic process | 8e-08 | 164 | 0.963 | 1 | 5838 |
| GO:0007051 | spindle organization | 1e-07 | 10 | 1.000 | 0 | 52 |
| GO:0016043 | cellular component organization | 3e-07 | 87 | 0.663 | 1 | 2471 |
| GO:0009987 | cellular process | 5e-07 | 214 | 0.527 | 4 | 8854 |
| GO:0022613 | ribonucleoprotein complex biogenesis | 8e-07 | 16 | 1.000 | 0 | 170 |
| GO:0010948 | negative regulation of cell cycle process | 1e-06 | 8 | 1.000 | 0 | 38 |
| GO:0006352 | transcription initiation | 1e-06 | 11 | 1.000 | 0 | 81 |
| GO:0007017 | microtubule-based process | 2e-06 | 18 | 1.000 | 0 | 224 |
| GO:0008152 | metabolic process | 2e-06 | 171 | 0.841 | 2 | 6427 |
| GO:0000226 | microtubule cytoskeleton organization | 3e-06 | 14 | 1.000 | 0 | 144 |
| GO:0034645 | cellular macromolecule biosynthetic process | 3e-06 | 87 | 0.685 | 1 | 2604 |
| GO:0030261 | chromosome condensation | 4e-06 | 6 | 1.000 | 0 | 21 |
| GO:0006310 | DNA recombination | 5e-06 | 12 | 1.000 | 0 | 111 |
| GO:0000070 | mitotic sister chromatid segregation | 8e-06 | 7 | 1.000 | 0 | 35 |
| GO:0009059 | macromolecule biosynthetic process | 8e-06 | 87 | 0.694 | 1 | 2665 |
| GO:0000819 | sister chromatid segregation | 1e-05 | 7 | 1.000 | 0 | 36 |
| GO:0022618 | ribonucleoprotein complex assembly | 1e-05 | 9 | 1.000 | 0 | 66 |
| GO:0006367 | transcription initiation from RNA polymerase II promoter | 1e-05 | 9 | 1.000 | 0 | 68 |
| GO:0044085 | cellular component biogenesis | 2e-05 | 42 | 1.000 | 0 | 1007 |
| GO:0050657 | nucleic acid transport | 2e-05 | 10 | 1.000 | 0 | 89 |
| GO:0050658 | RNA transport | 2e-05 | 10 | 1.000 | 0 | 89 |
| GO:0051236 | establishment of RNA localization | 2e-05 | 10 | 1.000 | 0 | 89 |
| GO:0032392 | DNA geometric change | 2e-05 | 5 | 1.000 | 0 | 17 |
| GO:0032508 | DNA duplex unwinding | 2e-05 | 5 | 1.000 | 0 | 17 |
| GO:0006403 | RNA localization | 2e-05 | 10 | 1.000 | 0 | 90 |
| GO:0043933 | macromolecular complex subunit organization | 3e-05 | 33 | 1.000 | 0 | 724 |
| GO:0000375 | RNA splicing, via transesterification reactions | 3e-05 | 10 | 1.000 | 0 | 94 |
| GO:0033554 | cellular response to stress | 3e-05 | 29 | 0.211 | 1 | 599 |
| GO:0015931 | nucleobase, nucleoside, nucleotide and nucleic acid transport | 6e-05 | 10 | 1.000 | 0 | 101 |
| GO:0070925 | organelle assembly | 8e-05 | 7 | 1.000 | 0 | 49 |
| GO:0034621 | cellular macromolecular complex subunit organization | 8e-05 | 21 | 1.000 | 0 | 384 |
| GO:0006323 | DNA packaging | 8e-05 | 9 | 1.000 | 0 | 85 |
| GO:0065003 | macromolecular complex assembly | 9e-05 | 30 | 1.000 | 0 | 665 |
| GO:0006325 | chromatin organization | 1e-04 | 19 | 1.000 | 0 | 339 |
| GO:0000075 | cell cycle checkpoint | 3e-04 | 9 | 1.000 | 0 | 98 |
| GO:0051028 | mRNA transport | 3e-04 | 8 | 1.000 | 0 | 78 |
| GO:0010564 | regulation of cell cycle process | 3e-04 | 11 | 1.000 | 0 | 144 |
| GO:0034622 | cellular macromolecular complex assembly | 3e-04 | 18 | 1.000 | 0 | 332 |
| GO:0006265 | DNA topological change | 3e-04 | 3 | 1.000 | 0 | 7 |
| GO:0006401 | RNA catabolic process | 4e-04 | 7 | 1.000 | 0 | 62 |
| GO:0007062 | sister chromatid cohesion | 4e-04 | 4 | 1.000 | 0 | 17 |
| GO:0022607 | cellular component assembly | 4e-04 | 35 | 1.000 | 0 | 898 |
| GO:0000733 | DNA strand renaturation | 5e-04 | 2 | 1.000 | 0 | 2 |
| GO:0033962 | cytoplasmic mRNA processing body assembly | 5e-04 | 2 | 1.000 | 0 | 2 |
| GO:0045875 | negative regulation of sister chromatid cohesion | 5e-04 | 2 | 1.000 | 0 | 2 |
| GO:0000377 | RNA splicing, via transesterification reactions with bulged adenosine as nucleophile | 5e-04 | 8 | 1.000 | 0 | 85 |
| GO:0000398 | nuclear mRNA splicing, via spliceosome | 5e-04 | 8 | 1.000 | 0 | 85 |
| GO:0009058 | biosynthetic process | 6e-04 | 96 | 0.392 | 2 | 3372 |
| GO:0044249 | cellular biosynthetic process | 7e-04 | 94 | 0.782 | 1 | 3289 |
| GO:0006457 | protein folding | 7e-04 | 10 | 1.000 | 0 | 135 |
| GO:0006338 | chromatin remodeling | 7e-04 | 6 | 1.000 | 0 | 50 |
| GO:0016568 | chromatin modification | 7e-04 | 15 | 1.000 | 0 | 270 |
| GO:0042254 | ribosome biogenesis | 8e-04 | 9 | 1.000 | 0 | 114 |
| GO:0051726 | regulation of cell cycle | 8e-04 | 19 | 1.000 | 0 | 393 |
| GO:0007091 | mitotic metaphase/anaphase transition | 9e-04 | 4 | 1.000 | 0 | 21 |
| GO:0030071 | regulation of mitotic metaphase/anaphase transition | 9e-04 | 4 | 1.000 | 0 | 21 |
| GO:0007088 | regulation of mitosis | 0.001 | 6 | 1.000 | 0 | 54 |
| GO:0051783 | regulation of nuclear division | 0.001 | 6 | 1.000 | 0 | 54 |
| GO:0006301 | postreplication repair | 0.001 | 3 | 1.000 | 0 | 10 |
| GO:0051983 | regulation of chromosome segregation | 0.001 | 3 | 1.000 | 0 | 10 |
| GO:0002200 | somatic diversification of immune receptors | 0.001 | 5 | 1.000 | 0 | 38 |
| GO:0006189 | 'de novo' IMP biosynthetic process | 0.001 | 2 | 1.000 | 0 | 3 |
| GO:0045002 | double-strand break repair via single-strand annealing | 0.001 | 2 | 1.000 | 0 | 3 |
| GO:0045292 | nuclear mRNA cis splicing, via spliceosome | 0.001 | 2 | 1.000 | 0 | 3 |
| GO:0045842 | positive regulation of mitotic metaphase/anaphase transition | 0.001 | 2 | 1.000 | 0 | 3 |
| GO:0046599 | regulation of centriole replication | 0.001 | 2 | 1.000 | 0 | 3 |
| GO:0071479 | cellular response to ionizing radiation | 0.001 | 2 | 1.000 | 0 | 3 |
| GO:0007076 | mitotic chromosome condensation | 0.001 | 3 | 1.000 | 0 | 11 |
| GO:0007094 | mitotic cell cycle spindle assembly checkpoint | 0.001 | 3 | 1.000 | 0 | 11 |
| GO:0045841 | negative regulation of mitotic metaphase/anaphase transition | 0.001 | 3 | 1.000 | 0 | 11 |
| GO:0051310 | metaphase plate congression | 0.001 | 3 | 1.000 | 0 | 11 |
| GO:0060255 | regulation of macromolecule metabolic process | 0.001 | 75 | 0.676 | 1 | 2551 |
| GO:0006302 | double-strand break repair | 0.002 | 6 | 1.000 | 0 | 59 |
| GO:0007346 | regulation of mitotic cell cycle | 0.002 | 10 | 1.000 | 0 | 153 |
| GO:0071174 | mitotic cell cycle spindle checkpoint | 0.002 | 3 | 1.000 | 0 | 12 |
| GO:0051053 | negative regulation of DNA metabolic process | 0.002 | 5 | 1.000 | 0 | 42 |
| GO:0051640 | organelle localization | 0.002 | 7 | 1.000 | 0 | 85 |
| GO:0071173 | spindle assembly checkpoint | 0.002 | 3 | 1.000 | 0 | 13 |
| GO:0006350 | transcription | 0.002 | 60 | 0.569 | 1 | 1974 |
| GO:0006188 | IMP biosynthetic process | 0.003 | 2 | 1.000 | 0 | 4 |
| GO:0030263 | apoptotic chromosome condensation | 0.003 | 2 | 1.000 | 0 | 4 |
| GO:0043570 | maintenance of DNA repeat elements | 0.003 | 2 | 1.000 | 0 | 4 |
| GO:0046040 | IMP metabolic process | 0.003 | 2 | 1.000 | 0 | 4 |
| GO:0006406 | mRNA export from nucleus | 0.003 | 4 | 1.000 | 0 | 28 |
| GO:0050000 | chromosome localization | 0.003 | 3 | 1.000 | 0 | 14 |
| GO:0051303 | establishment of chromosome localization | 0.003 | 3 | 1.000 | 0 | 14 |
| GO:0006368 | RNA elongation from RNA polymerase II promoter | 0.003 | 5 | 1.000 | 0 | 47 |
| GO:0010639 | negative regulation of organelle organization | 0.003 | 7 | 1.000 | 0 | 91 |
| GO:0031577 | spindle checkpoint | 0.004 | 3 | 1.000 | 0 | 15 |
| GO:0045839 | negative regulation of mitosis | 0.004 | 3 | 1.000 | 0 | 15 |
| GO:0051784 | negative regulation of nuclear division | 0.004 | 3 | 1.000 | 0 | 15 |
| GO:0051052 | regulation of DNA metabolic process | 0.004 | 8 | 1.000 | 0 | 118 |
| GO:0016445 | somatic diversification of immunoglobulins | 0.004 | 4 | 1.000 | 0 | 31 |
| GO:0006354 | RNA elongation | 0.004 | 5 | 1.000 | 0 | 50 |
| GO:0051716 | cellular response to stimulus | 0.004 | 33 | 0.320 | 1 | 954 |
| GO:0010556 | regulation of macromolecule biosynthetic process | 0.004 | 62 | 0.596 | 1 | 2106 |
| GO:0006999 | nuclear pore organization | 0.004 | 2 | 1.000 | 0 | 5 |
| GO:0007063 | regulation of sister chromatid cohesion | 0.004 | 2 | 1.000 | 0 | 5 |
| GO:0034501 | protein localization to kinetochore | 0.004 | 2 | 1.000 | 0 | 5 |
| GO:0051096 | positive regulation of helicase activity | 0.004 | 2 | 1.000 | 0 | 5 |
| GO:0051382 | kinetochore assembly | 0.004 | 2 | 1.000 | 0 | 5 |
| GO:0051383 | kinetochore organization | 0.004 | 2 | 1.000 | 0 | 5 |
| GO:0051297 | centrosome organization | 0.005 | 4 | 1.000 | 0 | 33 |
| GO:0006461 | protein complex assembly | 0.005 | 21 | 1.000 | 0 | 532 |
| GO:0070271 | protein complex biogenesis | 0.005 | 21 | 1.000 | 0 | 532 |
| GO:0045934 | negative regulation of nucleobase, nucleoside, nucleotide and nucleic acid metabolic process | 0.006 | 20 | 0.179 | 1 | 499 |
| GO:0031023 | microtubule organizing center organization | 0.006 | 4 | 1.000 | 0 | 34 |
| GO:0051172 | negative regulation of nitrogen compound metabolic process | 0.006 | 20 | 0.181 | 1 | 505 |
| GO:0009411 | response to UV | 0.006 | 5 | 1.000 | 0 | 55 |
| GO:0002566 | somatic diversification of immune receptors via somatic mutation | 0.007 | 2 | 1.000 | 0 | 6 |
| GO:0006264 | mitochondrial DNA replication | 0.007 | 2 | 1.000 | 0 | 6 |
| GO:0010824 | regulation of centrosome duplication | 0.007 | 2 | 1.000 | 0 | 6 |
| GO:0016446 | somatic hypermutation of immunoglobulin genes | 0.007 | 2 | 1.000 | 0 | 6 |
| GO:0010605 | negative regulation of macromolecule metabolic process | 0.007 | 26 | 0.250 | 1 | 721 |
| GO:0010212 | response to ionizing radiation | 0.007 | 5 | 1.000 | 0 | 56 |
| GO:0033043 | regulation of organelle organization | 0.007 | 11 | 1.000 | 0 | 216 |
| GO:0002562 | somatic diversification of immune receptors via germline recombination within a single locus | 0.007 | 4 | 1.000 | 0 | 36 |
| GO:0016444 | somatic cell DNA recombination | 0.007 | 4 | 1.000 | 0 | 36 |
| GO:0006298 | mismatch repair | 0.007 | 3 | 1.000 | 0 | 19 |
| GO:0006405 | RNA export from nucleus | 0.008 | 4 | 1.000 | 0 | 37 |
| GO:0080090 | regulation of primary metabolic process | 0.008 | 74 | 0.696 | 1 | 2672 |
| GO:0045333 | cellular respiration | 0.008 | 6 | 1.000 | 0 | 82 |
| GO:0051225 | spindle assembly | 0.009 | 3 | 1.000 | 0 | 20 |
| GO:0019219 | regulation of nucleobase, nucleoside, nucleotide and nucleic acid metabolic process | 0.009 | 63 | 0.616 | 1 | 2213 |
| GO:0009200 | deoxyribonucleoside triphosphate metabolic process | 0.009 | 2 | 1.000 | 0 | 7 |
| GO:0032042 | mitochondrial DNA metabolic process | 0.009 | 2 | 1.000 | 0 | 7 |
| GO:0034508 | centromere complex assembly | 0.009 | 2 | 1.000 | 0 | 7 |
| GO:0043044 | ATP-dependent chromatin remodeling | 0.009 | 2 | 1.000 | 0 | 7 |
| GO:0046605 | regulation of centrosome cycle | 0.009 | 2 | 1.000 | 0 | 7 |
| GO:0051095 | regulation of helicase activity | 0.009 | 2 | 1.000 | 0 | 7 |
| GO:0033044 | regulation of chromosome organization | 0.010 | 4 | 1.000 | 0 | 39 |
| GO:0045840 | positive regulation of mitosis | 0.010 | 3 | 1.000 | 0 | 21 |
| GO:0051785 | positive regulation of nuclear division | 0.010 | 3 | 1.000 | 0 | 21 |
| GO:0032502 | developmental process | 0.987 | 46 | 0.005 | 4 | 2770 |
| GO:0007275 | multicellular organismal development | 0.961 | 44 | 0.004 | 4 | 2529 |
| GO:0001501 | skeletal system development | 0.784 | 4 | 0.003 | 2 | 247 |
| GO:0048856 | anatomical structure development | 0.993 | 36 | 0.003 | 4 | 2332 |
| GO:0019221 | cytokine-mediated signaling pathway | 1.000 | 0 | 3e-04 | 2 | 80 |
| GO:0023036 | initiation of signal transduction | 1.000 | 0 | 3e-04 | 2 | 80 |
| GO:0023038 | signal initiation by diffusible mediator | 1.000 | 0 | 3e-04 | 2 | 80 |
| GO:0023049 | signal initiation by protein/peptide mediator | 1.000 | 0 | 3e-04 | 2 | 80 |

### Cellular Component

- 11181 Entrez Gene IDs have annotations in category 'CC'
- 239 of these are in the above list
- upreg means upregulated in group BAFF\_regulated.1 and downreg means downregulated in group BAFF\_regulated.1

|  |  |  |  |  |  |  |
| --- | --- | --- | --- | --- | --- | --- |
| **GO ID** | **GO Term** | **upreg. p-value** | **upreg. int. Count** | **downreg. p-value** | **downreg. int. Count** | **GO Count** |
| GO:0044428 | nuclear part | <2e-16 | 114 | 1.000 | 0 | 1625 |
| GO:0005634 | nucleus | <2e-16 | 165 | 0.880 | 1 | 3864 |
| GO:0031974 | membrane-enclosed lumen | <2e-16 | 97 | 1.000 | 0 | 1622 |
| GO:0031981 | nuclear lumen | <2e-16 | 85 | 1.000 | 0 | 1280 |
| GO:0070013 | intracellular organelle lumen | <2e-16 | 94 | 1.000 | 0 | 1555 |
| GO:0043233 | organelle lumen | <2e-16 | 94 | 1.000 | 0 | 1591 |
| GO:0044424 | intracellular part | <2e-16 | 226 | 0.979 | 2 | 8157 |
| GO:0043231 | intracellular membrane-bounded organelle | <2e-16 | 199 | 0.879 | 2 | 6261 |
| GO:0043227 | membrane-bounded organelle | <2e-16 | 199 | 0.879 | 2 | 6268 |
| GO:0044446 | intracellular organelle part | <2e-16 | 153 | 1.000 | 0 | 3939 |
| GO:0005622 | intracellular | <2e-16 | 227 | 0.984 | 2 | 8358 |
| GO:0044422 | organelle part | <2e-16 | 153 | 0.890 | 1 | 3992 |
| GO:0043229 | intracellular organelle | <2e-16 | 205 | 0.928 | 2 | 6933 |
| GO:0043226 | organelle | <2e-16 | 205 | 0.928 | 2 | 6947 |
| GO:0005654 | nucleoplasm | <2e-16 | 58 | 1.000 | 0 | 821 |
| GO:0032991 | macromolecular complex | 4e-14 | 106 | 0.731 | 1 | 2584 |
| GO:0005694 | chromosome | 5e-14 | 37 | 1.000 | 0 | 416 |
| GO:0043228 | non-membrane-bounded organelle | 2e-13 | 91 | 0.644 | 1 | 2087 |
| GO:0043232 | intracellular non-membrane-bounded organelle | 2e-13 | 91 | 0.644 | 1 | 2087 |
| GO:0000793 | condensed chromosome | 3e-12 | 19 | 1.000 | 0 | 117 |
| GO:0005681 | spliceosomal complex | 2e-10 | 17 | 1.000 | 0 | 113 |
| GO:0000777 | condensed chromosome kinetochore | 4e-10 | 13 | 1.000 | 0 | 62 |
| GO:0000779 | condensed chromosome, centromeric region | 8e-10 | 13 | 1.000 | 0 | 66 |
| GO:0005730 | nucleolus | 9e-10 | 38 | 1.000 | 0 | 604 |
| GO:0000775 | chromosome, centromeric region | 3e-09 | 16 | 1.000 | 0 | 118 |
| GO:0044427 | chromosomal part | 3e-09 | 27 | 1.000 | 0 | 344 |
| GO:0043234 | protein complex | 5e-09 | 82 | 0.654 | 1 | 2139 |
| GO:0000776 | kinetochore | 7e-09 | 13 | 1.000 | 0 | 78 |
| GO:0030529 | ribonucleoprotein complex | 8e-09 | 29 | 1.000 | 0 | 409 |
| GO:0005819 | spindle | 1e-08 | 17 | 1.000 | 0 | 146 |
| GO:0044451 | nucleoplasm part | 2e-08 | 32 | 1.000 | 0 | 508 |
| GO:0034399 | nuclear periphery | 6e-08 | 11 | 1.000 | 0 | 63 |
| GO:0000228 | nuclear chromosome | 1e-07 | 16 | 1.000 | 0 | 153 |
| GO:0015630 | microtubule cytoskeleton | 1e-07 | 29 | 1.000 | 0 | 465 |
| GO:0016363 | nuclear matrix | 2e-07 | 10 | 1.000 | 0 | 56 |
| GO:0000940 | outer kinetochore of condensed chromosome | 9e-07 | 5 | 1.000 | 0 | 10 |
| GO:0044464 | cell part | 5e-06 | 232 | 0.956 | 4 | 10383 |
| GO:0005623 | cell | 5e-06 | 232 | 0.956 | 4 | 10384 |
| GO:0016607 | nuclear speck | 7e-06 | 11 | 1.000 | 0 | 99 |
| GO:0000922 | spindle pole | 1e-05 | 8 | 1.000 | 0 | 52 |
| GO:0016604 | nuclear body | 2e-05 | 14 | 1.000 | 0 | 173 |
| GO:0005815 | microtubule organizing center | 2e-05 | 16 | 1.000 | 0 | 222 |
| GO:0031080 | Nup107-160 complex | 4e-05 | 4 | 1.000 | 0 | 10 |
| GO:0044454 | nuclear chromosome part | 4e-05 | 11 | 1.000 | 0 | 118 |
| GO:0044430 | cytoskeletal part | 1e-04 | 31 | 1.000 | 0 | 723 |
| GO:0000796 | condensin complex | 2e-04 | 3 | 1.000 | 0 | 6 |
| GO:0005643 | nuclear pore | 4e-04 | 7 | 1.000 | 0 | 64 |
| GO:0000176 | nuclear exosome (RNase complex) | 4e-04 | 2 | 1.000 | 0 | 2 |
| GO:0032302 | MutSbeta complex | 4e-04 | 2 | 1.000 | 0 | 2 |
| GO:0016591 | DNA-directed RNA polymerase II, holoenzyme | 5e-04 | 7 | 1.000 | 0 | 67 |
| GO:0044450 | microtubule organizing center part | 7e-04 | 6 | 1.000 | 0 | 52 |
| GO:0000428 | DNA-directed RNA polymerase complex | 8e-04 | 4 | 1.000 | 0 | 21 |
| GO:0030880 | RNA polymerase complex | 8e-04 | 4 | 1.000 | 0 | 21 |
| GO:0055029 | nuclear DNA-directed RNA polymerase complex | 8e-04 | 4 | 1.000 | 0 | 21 |
| GO:0046930 | pore complex | 0.001 | 7 | 1.000 | 0 | 77 |
| GO:0000178 | exosome (RNase complex) | 0.001 | 3 | 1.000 | 0 | 11 |
| GO:0005813 | centrosome | 0.003 | 9 | 1.000 | 0 | 139 |
| GO:0030532 | small nuclear ribonucleoprotein complex | 0.003 | 4 | 1.000 | 0 | 28 |
| GO:0005665 | DNA-directed RNA polymerase II, core complex | 0.003 | 3 | 1.000 | 0 | 14 |
| GO:0005635 | nuclear envelope | 0.003 | 11 | 1.000 | 0 | 198 |
| GO:0005737 | cytoplasm | 0.004 | 146 | 0.979 | 1 | 5993 |
| GO:0032300 | mismatch repair complex | 0.004 | 2 | 1.000 | 0 | 5 |
| GO:0005856 | cytoskeleton | 0.005 | 35 | 1.000 | 0 | 1065 |
| GO:0005669 | transcription factor TFIID complex | 0.005 | 3 | 1.000 | 0 | 17 |
| GO:0030530 | heterogeneous nuclear ribonucleoprotein complex | 0.005 | 3 | 1.000 | 0 | 17 |
| GO:0000790 | nuclear chromatin | 0.005 | 5 | 1.000 | 0 | 54 |
| GO:0042382 | paraspeckles | 0.006 | 2 | 1.000 | 0 | 6 |
| GO:0005678 | chromatin assembly complex | 0.009 | 2 | 1.000 | 0 | 7 |
| GO:0005832 | chaperonin-containing T-complex | 0.009 | 2 | 1.000 | 0 | 7 |

### Distribution of KEGG annotations

- Up regulated probes with KEGG annotations in above list: 144
- Down regulated probes with KEGG annotations in above list: 2
- The chip holds 7585 probes annotated to 214 pathways

|  |  |  |  |  |  |  |
| --- | --- | --- | --- | --- | --- | --- |
| **KEGG ID** | **Path Name** | **upreg.p.value** | **upreg.Int.Count** | **downreg.p.value** | **downreg.Int.Count** | **KEGG.Count** |
| 03040 | Spliceosome | 7e-09 | 19 | 1 | 0 | 203 |
| 00230 | Purine metabolism | 8e-08 | 18 | 1 | 0 | 211 |
| 03430 | Mismatch repair | 4e-05 | 6 | 1 | 0 | 35 |
| 00670 | One carbon pool by folate | 1e-04 | 5 | 1 | 0 | 27 |
| 03018 | RNA degradation | 2e-04 | 8 | 1 | 0 | 82 |
| 00240 | Pyrimidine metabolism | 2e-04 | 9 | 1 | 0 | 107 |
| 00790 | Folate biosynthesis | 2e-04 | 4 | 1 | 0 | 16 |
| 03020 | RNA polymerase | 3e-04 | 5 | 1 | 0 | 31 |
| 00290 | Valine, leucine and isoleucine biosynthesis | 0.003 | 3 | 1 | 0 | 16 |

#99CCCC #CCCCCC #E8E8E8

Annotations from:

- Data package 'hgu133a.db' version 2.4.5 packaged on 2010-09-23 21:50:14 UTC; mcarlson
- Data package 'GO.db' version 2.4.5 packaged on 2010-09-23 21:49:10 UTC; mcarlson
- Data package 'KEGG.db' version 2.4.5 packaged on 2010-09-23 22:03:46 UTC; mcarlson
