# Supplementary material for: Global gene expression changes of in vitro stimulated human transformed germinal centre B cells as surrogate for oncogenic pathway activation in individual aggressive B cell lymphomas
Source: Cell Commun Signal. 2012 Dec 20;10:43. doi: 10.1186/1478-811X-10-43 (PMC3566944; doi:10.1186/1478-811X-10-43)
Supplement: Additional file 9 — Supplemental 2. Geneset enrichment Analysis identifying enriched pathways in differentially expressed genes. [file 1478-811X-10-43-S9.zip › supplementalFile2_GO_AnalysenLIMMA/BAFF.2_dn.html]

- 123 unique Entrez Gene IDs considered
- on chip with 22283 probesets

- Molecular function
- Biological process
- Cellular component
- Pathways (KEGG)

### Molecular Function

- 10870 Entrez Gene IDs have annotations in category 'MF'
- 108 of these are in the above list
- upreg means upregulated in group BAFF\_regulated.2 and downreg means downregulated in group BAFF\_regulated.2

|  |  |  |  |  |  |  |
| --- | --- | --- | --- | --- | --- | --- |
| **GO ID** | **GO Term** | **upreg. p-value** | **upreg. int. Count** | **downreg. p-value** | **downreg. int. Count** | **GO Count** |
| GO:0004872 | receptor activity | 1.000 | 0 | 0.010 | 18 | 1046 |
| GO:0005198 | structural molecule activity | 1.000 | 0 | 0.009 | 11 | 507 |
| GO:0004930 | G-protein coupled receptor activity | 1.000 | 0 | 0.008 | 9 | 364 |
| GO:0005200 | structural constituent of cytoskeleton | 1.000 | 0 | 0.005 | 4 | 72 |
| GO:0015280 | amiloride-sensitive sodium channel activity | 1.000 | 0 | 0.002 | 2 | 8 |
| GO:0008083 | growth factor activity | 1.000 | 0 | 0.002 | 6 | 139 |
| GO:0004888 | transmembrane receptor activity | 1.000 | 0 | 0.001 | 16 | 729 |
| GO:0004871 | signal transducer activity | 1.000 | 0 | 7e-04 | 26 | 1416 |
| GO:0060089 | molecular transducer activity | 1.000 | 0 | 7e-04 | 26 | 1416 |

### Biological Process

- 10392 Entrez Gene IDs have annotations in category 'BP'
- 104 of these are in the above list
- upreg means upregulated in group BAFF\_regulated.2 and downreg means downregulated in group BAFF\_regulated.2

|  |  |  |  |  |  |  |
| --- | --- | --- | --- | --- | --- | --- |
| **GO ID** | **GO Term** | **upreg. p-value** | **upreg. int. Count** | **downreg. p-value** | **downreg. int. Count** | **GO Count** |
| GO:0007283 | spermatogenesis | 0.007 | 2 | 0.021 | 6 | 230 |
| GO:0048232 | male gamete generation | 0.007 | 2 | 0.021 | 6 | 230 |
| GO:0015674 | di-, tri-valent inorganic cation transport | 1.000 | 0 | 0.010 | 6 | 193 |
| GO:0006576 | cellular biogenic amine metabolic process | 1.000 | 0 | 0.009 | 4 | 86 |
| GO:0009605 | response to external stimulus | 1.000 | 0 | 0.009 | 12 | 584 |
| GO:0048699 | generation of neurons | 1.000 | 0 | 0.008 | 11 | 508 |
| GO:0048520 | positive regulation of behavior | 1.000 | 0 | 0.008 | 3 | 44 |
| GO:0051930 | regulation of sensory perception of pain | 1.000 | 0 | 0.007 | 2 | 14 |
| GO:0051931 | regulation of sensory perception | 1.000 | 0 | 0.007 | 2 | 14 |
| GO:0007267 | cell-cell signaling | 1.000 | 0 | 0.007 | 13 | 638 |
| GO:0030865 | cortical cytoskeleton organization | 1.000 | 0 | 0.006 | 2 | 13 |
| GO:0030866 | cortical actin cytoskeleton organization | 1.000 | 0 | 0.006 | 2 | 13 |
| GO:0031069 | hair follicle morphogenesis | 1.000 | 0 | 0.006 | 2 | 13 |
| GO:0050921 | positive regulation of chemotaxis | 1.000 | 0 | 0.006 | 3 | 40 |
| GO:0006935 | chemotaxis | 1.000 | 0 | 0.006 | 6 | 175 |
| GO:0042330 | taxis | 1.000 | 0 | 0.006 | 6 | 175 |
| GO:0009416 | response to light stimulus | 1.000 | 0 | 0.006 | 5 | 123 |
| GO:0032502 | developmental process | 0.505 | 2 | 0.006 | 38 | 2770 |
| GO:0009268 | response to pH | 1.000 | 0 | 0.005 | 2 | 12 |
| GO:0022008 | neurogenesis | 1.000 | 0 | 0.005 | 12 | 548 |
| GO:0048666 | neuron development | 1.000 | 0 | 0.005 | 9 | 344 |
| GO:0070838 | divalent metal ion transport | 1.000 | 0 | 0.005 | 6 | 166 |
| GO:0048513 | organ development | 1.000 | 0 | 0.005 | 26 | 1651 |
| GO:0002548 | monocyte chemotaxis | 1.000 | 0 | 0.005 | 2 | 11 |
| GO:0007168 | receptor guanylyl cyclase signaling pathway | 1.000 | 0 | 0.005 | 2 | 11 |
| GO:0006816 | calcium ion transport | 1.000 | 0 | 0.004 | 6 | 163 |
| GO:0021761 | limbic system development | 1.000 | 0 | 0.004 | 3 | 35 |
| GO:0030182 | neuron differentiation | 1.000 | 0 | 0.004 | 11 | 461 |
| GO:0007275 | multicellular organismal development | 0.812 | 1 | 0.004 | 36 | 2529 |
| GO:0021536 | diencephalon development | 1.000 | 0 | 0.004 | 3 | 34 |
| GO:0048731 | system development | 1.000 | 0 | 0.004 | 32 | 2157 |
| GO:0040036 | regulation of fibroblast growth factor receptor signaling pathway | 1.000 | 0 | 0.004 | 2 | 10 |
| GO:0050896 | response to stimulus | 1.000 | 0 | 0.002 | 40 | 2834 |
| GO:0009581 | detection of external stimulus | 1.000 | 0 | 0.002 | 4 | 60 |
| GO:0007167 | enzyme linked receptor protein signaling pathway | 0.224 | 1 | 0.002 | 11 | 430 |
| GO:0023052 | signaling | 0.872 | 1 | 0.002 | 42 | 3012 |
| GO:0009583 | detection of light stimulus | 1.000 | 0 | 0.002 | 3 | 28 |
| GO:0009628 | response to abiotic stimulus | 1.000 | 0 | 0.002 | 10 | 362 |
| GO:0007166 | cell surface receptor linked signaling pathway | 0.554 | 1 | 0.002 | 23 | 1309 |
| GO:0007399 | nervous system development | 1.000 | 0 | 0.002 | 19 | 990 |
| GO:0048856 | anatomical structure development | 1.000 | 0 | 0.002 | 35 | 2332 |
| GO:0045161 | neuronal ion channel clustering | 1.000 | 0 | 0.002 | 2 | 7 |
| GO:0007602 | phototransduction | 1.000 | 0 | 0.002 | 3 | 25 |
| GO:0007601 | visual perception | 1.000 | 0 | 0.002 | 7 | 180 |
| GO:0050953 | sensory perception of light stimulus | 1.000 | 0 | 0.002 | 7 | 180 |
| GO:0050909 | sensory perception of taste | 1.000 | 0 | 0.001 | 3 | 24 |
| GO:0009582 | detection of abiotic stimulus | 1.000 | 0 | 0.001 | 4 | 51 |
| GO:0071109 | superior temporal gyrus development | 1.000 | 0 | 0.001 | 2 | 6 |
| GO:0042634 | regulation of hair cycle | 1.000 | 0 | 9e-04 | 2 | 5 |
| GO:0007606 | sensory perception of chemical stimulus | 1.000 | 0 | 8e-04 | 5 | 78 |
| GO:0007626 | locomotory behavior | 1.000 | 0 | 8e-04 | 9 | 263 |
| GO:0008543 | fibroblast growth factor receptor signaling pathway | 1.000 | 0 | 6e-04 | 4 | 42 |
| GO:0021854 | hypothalamus development | 1.000 | 0 | 5e-04 | 2 | 4 |
| GO:0051797 | regulation of hair follicle development | 1.000 | 0 | 5e-04 | 2 | 4 |
| GO:0007610 | behavior | 1.000 | 0 | 2e-04 | 13 | 436 |
| GO:0007600 | sensory perception | 1.000 | 0 | 7e-06 | 14 | 364 |
| GO:0003008 | system process | 1.000 | 0 | 6e-06 | 26 | 1089 |
| GO:0032501 | multicellular organismal process | 0.661 | 2 | 2e-06 | 56 | 3528 |
| GO:0050877 | neurological system process | 1.000 | 0 | 1e-06 | 22 | 752 |

### Cellular Component

- 11181 Entrez Gene IDs have annotations in category 'CC'
- 107 of these are in the above list
- upreg means upregulated in group BAFF\_regulated.2 and downreg means downregulated in group BAFF\_regulated.2

|  |  |  |  |  |  |  |
| --- | --- | --- | --- | --- | --- | --- |
| **GO ID** | **GO Term** | **upreg. p-value** | **upreg. int. Count** | **downreg. p-value** | **downreg. int. Count** | **GO Count** |
| GO:0044459 | plasma membrane part | 1.000 | 0 | 0.008 | 25 | 1724 |
| GO:0032589 | neuron projection membrane | 1.000 | 0 | 0.005 | 2 | 12 |
| GO:0044463 | cell projection part | 1.000 | 0 | 0.004 | 8 | 283 |
| GO:0030863 | cortical cytoskeleton | 1.000 | 0 | 0.004 | 3 | 35 |
| GO:0030424 | axon | 1.000 | 0 | 0.003 | 6 | 156 |
| GO:0005886 | plasma membrane | 1.000 | 0 | 1e-04 | 41 | 2680 |

### Distribution of KEGG annotations

- Up regulated probes with KEGG annotations in above list: 3
- Down regulated probes with KEGG annotations in above list: 84
- The chip holds 7585 probes annotated to 214 pathways

|  |  |  |  |  |  |  |
| --- | --- | --- | --- | --- | --- | --- |
| **KEGG ID** | **Path Name** | **upreg.p.value** | **upreg.Int.Count** | **downreg.p.value** | **downreg.Int.Count** | **KEGG.Count** |
| 04142 | Lysosome | 2e-05 | 3 | 1.000 | 0 | 212 |
| 04010 | MAPK signaling pathway | 1 | 0 | 6e-04 | 14 | 474 |
| 04060 | Cytokine-cytokine receptor interaction | 1 | 0 | 0.003 | 11 | 374 |
| 04512 | ECM-receptor interaction | 1 | 0 | 0.003 | 7 | 177 |
| 04530 | Tight junction | 1 | 0 | 0.003 | 8 | 218 |
| 04742 | Taste transduction | 1 | 0 | 0.003 | 4 | 52 |
| 04950 | Maturity onset diabetes of the young | 1 | 0 | 0.009 | 3 | 39 |
| 04960 | Aldosterone-regulated sodium reabsorption | 1 | 0 | 0.007 | 4 | 69 |
| 05211 | Renal cell carcinoma | 1 | 0 | 2e-04 | 8 | 142 |

#99CCCC #CCCCCC #E8E8E8

Annotations from:

- Data package 'hgu133a.db' version 2.4.5 packaged on 2010-09-23 21:50:14 UTC; mcarlson
- Data package 'GO.db' version 2.4.5 packaged on 2010-09-23 21:49:10 UTC; mcarlson
- Data package 'KEGG.db' version 2.4.5 packaged on 2010-09-23 22:03:46 UTC; mcarlson
