# Supplementary material for: Global gene expression changes of in vitro stimulated human transformed germinal centre B cells as surrogate for oncogenic pathway activation in individual aggressive B cell lymphomas
Source: Cell Commun Signal. 2012 Dec 20;10:43. doi: 10.1186/1478-811X-10-43 (PMC3566944; doi:10.1186/1478-811X-10-43)
Supplement: Additional file 9 — Supplemental 2. Geneset enrichment Analysis identifying enriched pathways in differentially expressed genes. [file 1478-811X-10-43-S9.zip › supplementalFile2_GO_AnalysenLIMMA/BCR.1_up.html]

- 303 unique Entrez Gene IDs considered
- on chip with 22283 probesets

- Molecular function
- Biological process
- Cellular component
- Pathways (KEGG)

### Molecular Function

- 10870 Entrez Gene IDs have annotations in category 'MF'
- 272 of these are in the above list
- upreg means upregulated in group BCR\_regulated.1 and downreg means downregulated in group BCR\_regulated.1

|  |  |  |  |  |  |  |
| --- | --- | --- | --- | --- | --- | --- |
| **GO ID** | **GO Term** | **upreg. p-value** | **upreg. int. Count** | **downreg. p-value** | **downreg. int. Count** | **GO Count** |
| GO:0032405 | MutLalpha complex binding | 1.00 | 0 | 0.009 | 2 | 6 |
| GO:0070087 | chromo shadow domain binding | 1.00 | 0 | 0.009 | 2 | 6 |
| GO:0004536 | deoxyribonuclease activity | 1.00 | 0 | 0.008 | 4 | 32 |
| GO:0004540 | ribonuclease activity | 1.00 | 0 | 0.007 | 5 | 48 |
| GO:0004523 | ribonuclease H activity | 1.00 | 0 | 0.006 | 2 | 5 |
| GO:0003723 | RNA binding | 1.00 | 0 | 0.005 | 25 | 574 |
| GO:0032135 | DNA insertion or deletion binding | 1.00 | 0 | 0.004 | 2 | 4 |
| GO:0032137 | guanine/thymine mispair binding | 1.00 | 0 | 0.004 | 2 | 4 |
| GO:0003824 | catalytic activity | 1.00 | 0 | 0.003 | 120 | 3938 |
| GO:0030983 | mismatched DNA binding | 1.00 | 0 | 0.003 | 3 | 12 |
| GO:0008094 | DNA-dependent ATPase activity | 1.00 | 0 | 0.002 | 6 | 55 |
| GO:0004519 | endonuclease activity | 1.00 | 0 | 0.002 | 7 | 73 |
| GO:0003697 | single-stranded DNA binding | 1.00 | 0 | 0.002 | 6 | 54 |
| GO:0000400 | four-way junction DNA binding | 1.00 | 0 | 0.002 | 2 | 3 |
| GO:0004826 | phenylalanine-tRNA ligase activity | 1.00 | 0 | 0.002 | 2 | 3 |
| GO:0032138 | single base insertion or deletion binding | 1.00 | 0 | 0.002 | 2 | 3 |
| GO:0032139 | dinucleotide insertion or deletion binding | 1.00 | 0 | 0.002 | 2 | 3 |
| GO:0032142 | single guanine insertion binding | 1.00 | 0 | 0.002 | 2 | 3 |
| GO:0032356 | oxidized DNA binding | 1.00 | 0 | 0.002 | 2 | 3 |
| GO:0032357 | oxidized purine DNA binding | 1.00 | 0 | 0.002 | 2 | 3 |
| GO:0004521 | endoribonuclease activity | 1.00 | 0 | 0.002 | 5 | 35 |
| GO:0003774 | motor activity | 1.00 | 0 | 0.001 | 9 | 108 |
| GO:0045182 | translation regulator activity | 1.00 | 0 | 0.001 | 4 | 20 |
| GO:0000217 | DNA secondary structure binding | 1.00 | 0 | 0.001 | 3 | 9 |
| GO:0004518 | nuclease activity | 1.00 | 0 | 8e-04 | 10 | 119 |
| GO:0032181 | dinucleotide repeat insertion binding | 1.00 | 0 | 6e-04 | 2 | 2 |
| GO:0070259 | tyrosyl-DNA phosphodiesterase activity | 1.00 | 0 | 6e-04 | 2 | 2 |
| GO:0003688 | DNA replication origin binding | 1.00 | 0 | 5e-04 | 3 | 7 |
| GO:0016893 | endonuclease activity, active with either ribo- or deoxyribonucleic acids and producing 5'-phosphomonoesters | 1.00 | 0 | 5e-04 | 5 | 27 |
| GO:0005515 | protein binding | 0.60 | 1 | 5e-04 | 190 | 6561 |
| GO:0016891 | endoribonuclease activity, producing 5'-phosphomonoesters | 1.00 | 0 | 2e-04 | 5 | 23 |
| GO:0003689 | DNA clamp loader activity | 1.00 | 0 | 1e-04 | 3 | 5 |
| GO:0033170 | protein-DNA loading ATPase activity | 1.00 | 0 | 1e-04 | 3 | 5 |
| GO:0003690 | double-stranded DNA binding | 1.00 | 0 | 8e-05 | 10 | 90 |
| GO:0003684 | damaged DNA binding | 1.00 | 0 | 4e-05 | 7 | 39 |
| GO:0005488 | binding | 0.84 | 1 | 3e-05 | 250 | 9129 |
| GO:0043566 | structure-specific DNA binding | 1.00 | 0 | 3e-05 | 13 | 136 |
| GO:0003677 | DNA binding | 0.13 | 1 | 1e-05 | 60 | 1386 |
| GO:0016817 | hydrolase activity, acting on acid anhydrides | 1.00 | 0 | 9e-06 | 34 | 613 |
| GO:0016818 | hydrolase activity, acting on acid anhydrides, in phosphorus-containing anhydrides | 1.00 | 0 | 9e-06 | 34 | 611 |
| GO:0017111 | nucleoside-triphosphatase activity | 1.00 | 0 | 9e-06 | 33 | 584 |
| GO:0016462 | pyrophosphatase activity | 1.00 | 0 | 8e-06 | 34 | 608 |
| GO:0003777 | microtubule motor activity | 1.00 | 0 | 6e-06 | 9 | 53 |
| GO:0019237 | centromeric DNA binding | 1.00 | 0 | 5e-06 | 4 | 6 |
| GO:0001882 | nucleoside binding | 1.00 | 0 | 8e-07 | 61 | 1311 |
| GO:0000166 | nucleotide binding | 1.00 | 0 | 6e-07 | 76 | 1776 |
| GO:0001883 | purine nucleoside binding | 1.00 | 0 | 6e-07 | 61 | 1301 |
| GO:0030554 | adenyl nucleotide binding | 1.00 | 0 | 2e-07 | 61 | 1268 |
| GO:0017076 | purine nucleotide binding | 1.00 | 0 | 2e-07 | 70 | 1528 |
| GO:0032559 | adenyl ribonucleotide binding | 1.00 | 0 | 8e-08 | 60 | 1202 |
| GO:0032553 | ribonucleotide binding | 1.00 | 0 | 6e-08 | 69 | 1460 |
| GO:0032555 | purine ribonucleotide binding | 1.00 | 0 | 6e-08 | 69 | 1460 |
| GO:0005524 | ATP binding | 1.00 | 0 | 5e-08 | 60 | 1183 |
| GO:0003676 | nucleic acid binding | 0.19 | 1 | 3e-08 | 89 | 2069 |

### Biological Process

- 10392 Entrez Gene IDs have annotations in category 'BP'
- 259 of these are in the above list
- upreg means upregulated in group BCR\_regulated.1 and downreg means downregulated in group BCR\_regulated.1

|  |  |  |  |  |  |  |
| --- | --- | --- | --- | --- | --- | --- |
| **GO ID** | **GO Term** | **upreg. p-value** | **upreg. int. Count** | **downreg. p-value** | **downreg. int. Count** | **GO Count** |
| GO:0031324 | negative regulation of cellular metabolic process | 1.000 | 0 | 0.010 | 28 | 710 |
| GO:0008380 | RNA splicing | 1.000 | 0 | 0.010 | 13 | 251 |
| GO:0007019 | microtubule depolymerization | 1.000 | 0 | 0.009 | 3 | 18 |
| GO:0031572 | G2/M transition DNA damage checkpoint | 1.000 | 0 | 0.009 | 3 | 18 |
| GO:0002566 | somatic diversification of immune receptors via somatic mutation | 1.000 | 0 | 0.009 | 2 | 6 |
| GO:0010390 | histone monoubiquitination | 1.000 | 0 | 0.009 | 2 | 6 |
| GO:0016446 | somatic hypermutation of immunoglobulin genes | 1.000 | 0 | 0.009 | 2 | 6 |
| GO:0033523 | histone H2B ubiquitination | 1.000 | 0 | 0.009 | 2 | 6 |
| GO:0085020 | protein K6-linked ubiquitination | 1.000 | 0 | 0.009 | 2 | 6 |
| GO:0051188 | cofactor biosynthetic process | 1.000 | 0 | 0.008 | 7 | 93 |
| GO:0016569 | covalent chromatin modification | 1.000 | 0 | 0.007 | 9 | 138 |
| GO:0010639 | negative regulation of organelle organization | 1.000 | 0 | 0.007 | 7 | 91 |
| GO:0009314 | response to radiation | 1.000 | 0 | 0.007 | 11 | 187 |
| GO:0016570 | histone modification | 1.000 | 0 | 0.007 | 9 | 136 |
| GO:0007163 | establishment or maintenance of cell polarity | 1.000 | 0 | 0.007 | 5 | 48 |
| GO:0000245 | spliceosome assembly | 1.000 | 0 | 0.006 | 4 | 30 |
| GO:0007063 | regulation of sister chromatid cohesion | 1.000 | 0 | 0.006 | 2 | 5 |
| GO:0009120 | deoxyribonucleoside metabolic process | 1.000 | 0 | 0.006 | 2 | 5 |
| GO:0032506 | cytokinetic process | 1.000 | 0 | 0.006 | 2 | 5 |
| GO:0051096 | positive regulation of helicase activity | 1.000 | 0 | 0.006 | 2 | 5 |
| GO:0090224 | regulation of spindle organization | 1.000 | 0 | 0.006 | 2 | 5 |
| GO:0031577 | spindle checkpoint | 1.000 | 0 | 0.006 | 3 | 15 |
| GO:0050684 | regulation of mRNA processing | 1.000 | 0 | 0.006 | 3 | 15 |
| GO:0000377 | RNA splicing, via transesterification reactions with bulged adenosine as nucleophile | 1.000 | 0 | 0.005 | 7 | 85 |
| GO:0000398 | nuclear mRNA splicing, via spliceosome | 1.000 | 0 | 0.005 | 7 | 85 |
| GO:0006323 | DNA packaging | 1.000 | 0 | 0.005 | 7 | 85 |
| GO:0009059 | macromolecule biosynthetic process | 0.256 | 1 | 0.005 | 85 | 2665 |
| GO:0051439 | regulation of ubiquitin-protein ligase activity involved in mitotic cell cycle | 1.000 | 0 | 0.005 | 6 | 63 |
| GO:0071103 | DNA conformation change | 1.000 | 0 | 0.004 | 8 | 104 |
| GO:0034645 | cellular macromolecule biosynthetic process | 0.251 | 1 | 0.004 | 84 | 2604 |
| GO:0051053 | negative regulation of DNA metabolic process | 1.000 | 0 | 0.004 | 5 | 42 |
| GO:0032259 | methylation | 1.000 | 0 | 0.004 | 6 | 60 |
| GO:0043414 | macromolecule methylation | 1.000 | 0 | 0.004 | 6 | 60 |
| GO:0000212 | meiotic spindle organization | 1.000 | 0 | 0.004 | 2 | 4 |
| GO:0000920 | cytokinetic cell separation | 1.000 | 0 | 0.004 | 2 | 4 |
| GO:0031440 | regulation of mRNA 3'-end processing | 1.000 | 0 | 0.004 | 2 | 4 |
| GO:0043570 | maintenance of DNA repeat elements | 1.000 | 0 | 0.004 | 2 | 4 |
| GO:0060236 | regulation of mitotic spindle organization | 1.000 | 0 | 0.004 | 2 | 4 |
| GO:0034621 | cellular macromolecular complex subunit organization | 1.000 | 0 | 0.003 | 19 | 384 |
| GO:0044238 | primary metabolic process | 0.562 | 1 | 0.003 | 167 | 5838 |
| GO:0031123 | RNA 3'-end processing | 1.000 | 0 | 0.003 | 4 | 24 |
| GO:0031570 | DNA integrity checkpoint | 1.000 | 0 | 0.003 | 6 | 56 |
| GO:0000375 | RNA splicing, via transesterification reactions | 1.000 | 0 | 0.002 | 8 | 94 |
| GO:0006289 | nucleotide-excision repair | 1.000 | 0 | 0.002 | 6 | 54 |
| GO:0007088 | regulation of mitosis | 1.000 | 0 | 0.002 | 6 | 54 |
| GO:0051783 | regulation of nuclear division | 1.000 | 0 | 0.002 | 6 | 54 |
| GO:0070507 | regulation of microtubule cytoskeleton organization | 1.000 | 0 | 0.002 | 5 | 37 |
| GO:0000077 | DNA damage checkpoint | 1.000 | 0 | 0.002 | 6 | 53 |
| GO:0016071 | mRNA metabolic process | 1.000 | 0 | 0.002 | 17 | 310 |
| GO:0045132 | meiotic chromosome segregation | 1.000 | 0 | 0.002 | 2 | 3 |
| GO:0045292 | nuclear mRNA cis splicing, via spliceosome | 1.000 | 0 | 0.002 | 2 | 3 |
| GO:0006396 | RNA processing | 1.000 | 0 | 0.002 | 23 | 475 |
| GO:0007127 | meiosis I | 1.000 | 0 | 0.001 | 5 | 34 |
| GO:0006412 | translation | 1.000 | 0 | 0.001 | 18 | 327 |
| GO:0000724 | double-strand break repair via homologous recombination | 1.000 | 0 | 0.001 | 4 | 20 |
| GO:0000725 | recombinational repair | 1.000 | 0 | 0.001 | 4 | 20 |
| GO:0030010 | establishment of cell polarity | 1.000 | 0 | 0.001 | 4 | 20 |
| GO:0051716 | cellular response to stimulus | 0.092 | 1 | 0.001 | 39 | 954 |
| GO:0007143 | female meiosis | 1.000 | 0 | 0.001 | 3 | 9 |
| GO:0007093 | mitotic cell cycle checkpoint | 1.000 | 0 | 0.001 | 6 | 48 |
| GO:0033043 | regulation of organelle organization | 1.000 | 0 | 1e-03 | 14 | 216 |
| GO:0031124 | mRNA 3'-end processing | 1.000 | 0 | 9e-04 | 4 | 18 |
| GO:0007099 | centriole replication | 1.000 | 0 | 8e-04 | 3 | 8 |
| GO:0022616 | DNA strand elongation | 1.000 | 0 | 8e-04 | 3 | 8 |
| GO:0006297 | nucleotide-excision repair, DNA gap filling | 1.000 | 0 | 7e-04 | 4 | 17 |
| GO:0007062 | sister chromatid cohesion | 1.000 | 0 | 7e-04 | 4 | 17 |
| GO:0000236 | mitotic prometaphase | 1.000 | 0 | 6e-04 | 2 | 2 |
| GO:0051299 | centrosome separation | 1.000 | 0 | 6e-04 | 2 | 2 |
| GO:0032886 | regulation of microtubule-based process | 1.000 | 0 | 5e-04 | 6 | 42 |
| GO:0046605 | regulation of centrosome cycle | 1.000 | 0 | 5e-04 | 3 | 7 |
| GO:0010212 | response to ionizing radiation | 1.000 | 0 | 4e-04 | 7 | 56 |
| GO:0051298 | centrosome duplication | 1.000 | 0 | 4e-04 | 4 | 15 |
| GO:0033044 | regulation of chromosome organization | 1.000 | 0 | 4e-04 | 6 | 39 |
| GO:0044237 | cellular metabolic process | 0.550 | 1 | 3e-04 | 169 | 5718 |
| GO:0010824 | regulation of centrosome duplication | 1.000 | 0 | 3e-04 | 3 | 6 |
| GO:0006270 | DNA-dependent DNA replication initiation | 1.000 | 0 | 3e-04 | 5 | 24 |
| GO:0006397 | mRNA processing | 1.000 | 0 | 2e-04 | 17 | 257 |
| GO:0007126 | meiosis | 1.000 | 0 | 2e-04 | 9 | 83 |
| GO:0051327 | M phase of meiotic cell cycle | 1.000 | 0 | 2e-04 | 9 | 83 |
| GO:0006271 | DNA strand elongation involved in DNA replication | 1.000 | 0 | 1e-04 | 3 | 5 |
| GO:0034501 | protein localization to kinetochore | 1.000 | 0 | 1e-04 | 3 | 5 |
| GO:0007346 | regulation of mitotic cell cycle | 1.000 | 0 | 1e-04 | 13 | 153 |
| GO:0000910 | cytokinesis | 1.000 | 0 | 1e-04 | 7 | 45 |
| GO:0051310 | metaphase plate congression | 1.000 | 0 | 1e-04 | 4 | 11 |
| GO:0051983 | regulation of chromosome segregation | 1.000 | 0 | 7e-05 | 4 | 10 |
| GO:0007018 | microtubule-based movement | 1.000 | 0 | 7e-05 | 10 | 89 |
| GO:0051321 | meiotic cell cycle | 1.000 | 0 | 5e-05 | 10 | 85 |
| GO:0007080 | mitotic metaphase plate congression | 1.000 | 0 | 4e-05 | 4 | 9 |
| GO:0051656 | establishment of organelle localization | 1.000 | 0 | 3e-05 | 9 | 64 |
| GO:0006310 | DNA recombination | 1.000 | 0 | 2e-05 | 12 | 111 |
| GO:0006807 | nitrogen compound metabolic process | 0.344 | 1 | 2e-05 | 121 | 3573 |
| GO:0031023 | microtubule organizing center organization | 1.000 | 0 | 2e-05 | 7 | 34 |
| GO:0046599 | regulation of centriole replication | 1.000 | 0 | 2e-05 | 3 | 3 |
| GO:0050000 | chromosome localization | 1.000 | 0 | 2e-05 | 5 | 14 |
| GO:0051303 | establishment of chromosome localization | 1.000 | 0 | 2e-05 | 5 | 14 |
| GO:0043170 | macromolecule metabolic process | 0.459 | 1 | 2e-05 | 152 | 4772 |
| GO:0006302 | double-strand break repair | 1.000 | 0 | 1e-05 | 9 | 59 |
| GO:0051297 | centrosome organization | 1.000 | 0 | 1e-05 | 7 | 33 |
| GO:0000132 | establishment of mitotic spindle orientation | 1.000 | 0 | 1e-05 | 4 | 7 |
| GO:0051294 | establishment of spindle orientation | 1.000 | 0 | 1e-05 | 4 | 7 |
| GO:0007010 | cytoskeleton organization | 1.000 | 0 | 1e-05 | 27 | 438 |
| GO:0009987 | cellular process | 0.852 | 1 | 1e-05 | 242 | 8854 |
| GO:0007052 | mitotic spindle organization | 1.000 | 0 | 9e-06 | 6 | 21 |
| GO:0006261 | DNA-dependent DNA replication | 1.000 | 0 | 5e-06 | 10 | 67 |
| GO:0034641 | cellular nitrogen compound metabolic process | 0.335 | 1 | 4e-06 | 121 | 3479 |
| GO:0016043 | cellular component organization | 1.000 | 0 | 3e-06 | 94 | 2471 |
| GO:0051293 | establishment of spindle localization | 1.000 | 0 | 2e-06 | 5 | 10 |
| GO:0051653 | spindle localization | 1.000 | 0 | 2e-06 | 5 | 10 |
| GO:0051726 | regulation of cell cycle | 0.038 | 1 | 2e-06 | 27 | 393 |
| GO:0051640 | organelle localization | 1.000 | 0 | 1e-06 | 12 | 85 |
| GO:0040001 | establishment of mitotic spindle localization | 1.000 | 0 | 1e-06 | 5 | 9 |
| GO:0000075 | cell cycle checkpoint | 1.000 | 0 | 8e-07 | 13 | 98 |
| GO:0007098 | centrosome cycle | 1.000 | 0 | 7e-07 | 7 | 22 |
| GO:0006139 | nucleobase, nucleoside, nucleotide and nucleic acid metabolic process | 0.311 | 1 | 4e-07 | 118 | 3229 |
| GO:0033554 | cellular response to stress | 1.000 | 0 | 2e-07 | 37 | 599 |
| GO:0051276 | chromosome organization | 1.000 | 0 | 2e-07 | 31 | 448 |
| GO:0010564 | regulation of cell cycle process | 1.000 | 0 | 9e-08 | 17 | 144 |
| GO:0044260 | cellular macromolecule metabolic process | 0.416 | 1 | 9e-08 | 149 | 4325 |
| GO:0090304 | nucleic acid metabolic process | 0.266 | 1 | 3e-08 | 109 | 2768 |
| GO:0000819 | sister chromatid segregation | 1.000 | 0 | 1e-08 | 10 | 36 |
| GO:0000070 | mitotic sister chromatid segregation | 1.000 | 0 | 8e-09 | 10 | 35 |
| GO:0006260 | DNA replication | 1.000 | 0 | 8e-09 | 22 | 206 |
| GO:0007051 | spindle organization | 1.000 | 0 | 4e-09 | 12 | 52 |
| GO:0006974 | response to DNA damage stimulus | 1.000 | 0 | 2e-11 | 33 | 347 |
| GO:0006281 | DNA repair | 1.000 | 0 | 5e-12 | 29 | 254 |
| GO:0007059 | chromosome segregation | 1.000 | 0 | 3e-13 | 18 | 77 |
| GO:0051301 | cell division | 1.000 | 0 | 2e-13 | 33 | 294 |
| GO:0000226 | microtubule cytoskeleton organization | 1.000 | 0 | 1e-13 | 24 | 144 |
| GO:0006996 | organelle organization | 1.000 | 0 | 5e-14 | 75 | 1238 |
| GO:0000278 | mitotic cell cycle | 1.000 | 0 | 7e-15 | 42 | 423 |
| GO:0007017 | microtubule-based process | 1.000 | 0 | 6e-16 | 32 | 224 |
| GO:0006259 | DNA metabolic process | 1.000 | 0 | <2e-16 | 50 | 494 |
| GO:0048285 | organelle fission | 1.000 | 0 | <2e-16 | 36 | 239 |
| GO:0022403 | cell cycle phase | 1.000 | 0 | <2e-16 | 48 | 432 |
| GO:0000280 | nuclear division | 1.000 | 0 | <2e-16 | 36 | 230 |
| GO:0007067 | mitosis | 1.000 | 0 | <2e-16 | 36 | 230 |
| GO:0000087 | M phase of mitotic cell cycle | 1.000 | 0 | <2e-16 | 37 | 238 |
| GO:0007049 | cell cycle | 0.081 | 1 | <2e-16 | 70 | 837 |
| GO:0000279 | M phase | 1.000 | 0 | <2e-16 | 45 | 328 |
| GO:0022402 | cell cycle process | 1.000 | 0 | <2e-16 | 59 | 566 |

### Cellular Component

- 11181 Entrez Gene IDs have annotations in category 'CC'
- 268 of these are in the above list
- upreg means upregulated in group BCR\_regulated.1 and downreg means downregulated in group BCR\_regulated.1

|  |  |  |  |  |  |  |
| --- | --- | --- | --- | --- | --- | --- |
| **GO ID** | **GO Term** | **upreg. p-value** | **upreg. int. Count** | **downreg. p-value** | **downreg. int. Count** | **GO Count** |
| GO:0005881 | cytoplasmic microtubule | 1.000 | 0 | 0.010 | 3 | 19 |
| GO:0000796 | condensin complex | 1.000 | 0 | 0.008 | 2 | 6 |
| GO:0070531 | BRCA1-A complex | 1.000 | 0 | 0.008 | 2 | 6 |
| GO:0005856 | cytoskeleton | 1.000 | 0 | 0.008 | 38 | 1065 |
| GO:0005737 | cytoplasm | 0.536 | 1 | 0.005 | 164 | 5993 |
| GO:0000145 | exocyst | 1.000 | 0 | 0.005 | 2 | 5 |
| GO:0005664 | nuclear origin of replication recognition complex | 1.000 | 0 | 0.005 | 2 | 5 |
| GO:0005828 | kinetochore microtubule | 1.000 | 0 | 0.005 | 2 | 5 |
| GO:0032300 | mismatch repair complex | 1.000 | 0 | 0.005 | 2 | 5 |
| GO:0005871 | kinesin complex | 1.000 | 0 | 0.005 | 3 | 15 |
| GO:0008023 | transcription elongation factor complex | 1.000 | 0 | 0.005 | 3 | 15 |
| GO:0005657 | replication fork | 1.000 | 0 | 0.005 | 4 | 29 |
| GO:0051233 | spindle midzone | 1.000 | 0 | 0.003 | 2 | 4 |
| GO:0016607 | nuclear speck | 1.000 | 0 | 0.003 | 8 | 99 |
| GO:0030496 | midbody | 1.000 | 0 | 0.002 | 4 | 24 |
| GO:0000315 | organellar large ribosomal subunit | 1.000 | 0 | 0.002 | 3 | 11 |
| GO:0005762 | mitochondrial large ribosomal subunit | 1.000 | 0 | 0.002 | 3 | 11 |
| GO:0016593 | Cdc73/Paf1 complex | 1.000 | 0 | 0.002 | 2 | 3 |
| GO:0032133 | chromosome passenger complex | 1.000 | 0 | 0.002 | 2 | 3 |
| GO:0045254 | pyruvate dehydrogenase complex | 1.000 | 0 | 0.002 | 2 | 3 |
| GO:0000152 | nuclear ubiquitin ligase complex | 1.000 | 0 | 0.002 | 4 | 22 |
| GO:0005623 | cell | 0.929 | 1 | 1e-03 | 260 | 10384 |
| GO:0044464 | cell part | 0.929 | 1 | 1e-03 | 260 | 10383 |
| GO:0000939 | inner kinetochore of condensed chromosome | 1.000 | 0 | 6e-04 | 2 | 2 |
| GO:0031436 | BRCA1-BARD1 complex | 1.000 | 0 | 6e-04 | 2 | 2 |
| GO:0032302 | MutSbeta complex | 1.000 | 0 | 6e-04 | 2 | 2 |
| GO:0044429 | mitochondrial part | 1.000 | 0 | 5e-04 | 24 | 479 |
| GO:0044451 | nucleoplasm part | 1.000 | 0 | 5e-04 | 25 | 508 |
| GO:0005840 | ribosome | 1.000 | 0 | 3e-04 | 12 | 154 |
| GO:0005663 | DNA replication factor C complex | 1.000 | 0 | 3e-04 | 3 | 6 |
| GO:0005759 | mitochondrial matrix | 1.000 | 0 | 2e-04 | 14 | 188 |
| GO:0031980 | mitochondrial lumen | 1.000 | 0 | 2e-04 | 14 | 188 |
| GO:0015030 | Cajal body | 1.000 | 0 | 1e-04 | 6 | 34 |
| GO:0000794 | condensed nuclear chromosome | 1.000 | 0 | 1e-04 | 7 | 46 |
| GO:0000313 | organellar ribosome | 1.000 | 0 | 9e-05 | 6 | 32 |
| GO:0005761 | mitochondrial ribosome | 1.000 | 0 | 9e-05 | 6 | 32 |
| GO:0000785 | chromatin | 1.000 | 0 | 8e-05 | 14 | 175 |
| GO:0000780 | condensed nuclear chromosome, centromeric region | 1.000 | 0 | 6e-05 | 4 | 10 |
| GO:0000808 | origin recognition complex | 1.000 | 0 | 2e-05 | 4 | 8 |
| GO:0016604 | nuclear body | 1.000 | 0 | 2e-05 | 15 | 173 |
| GO:0000942 | outer kinetochore of condensed nuclear chromosome | 1.000 | 0 | 1e-05 | 3 | 3 |
| GO:0044430 | cytoskeletal part | 1.000 | 0 | 8e-06 | 37 | 723 |
| GO:0005814 | centriole | 1.000 | 0 | 7e-06 | 7 | 31 |
| GO:0044454 | nuclear chromosome part | 1.000 | 0 | 5e-06 | 13 | 118 |
| GO:0044450 | microtubule organizing center part | 1.000 | 0 | 3e-06 | 9 | 52 |
| GO:0000940 | outer kinetochore of condensed chromosome | 1.000 | 0 | 2e-06 | 5 | 10 |
| GO:0030529 | ribonucleoprotein complex | 1.000 | 0 | 2e-06 | 27 | 409 |
| GO:0005874 | microtubule | 1.000 | 0 | 1e-06 | 19 | 216 |
| GO:0000228 | nuclear chromosome | 1.000 | 0 | 7e-07 | 16 | 153 |
| GO:0005876 | spindle microtubule | 1.000 | 0 | 6e-07 | 8 | 32 |
| GO:0043234 | protein complex | 1.000 | 0 | 4e-07 | 85 | 2139 |
| GO:0000778 | condensed nuclear chromosome kinetochore | 1.000 | 0 | 3e-07 | 4 | 4 |
| GO:0005813 | centrosome | 1.000 | 0 | 2e-07 | 16 | 139 |
| GO:0000922 | spindle pole | 1.000 | 0 | 3e-08 | 11 | 52 |
| GO:0005815 | microtubule organizing center | 1.000 | 0 | 2e-08 | 22 | 222 |
| GO:0005739 | mitochondrion | 1.000 | 0 | 4e-09 | 53 | 956 |
| GO:0000779 | condensed chromosome, centromeric region | 1.000 | 0 | 4e-09 | 13 | 66 |
| GO:0000777 | condensed chromosome kinetochore | 1.000 | 0 | 2e-09 | 13 | 62 |
| GO:0000776 | kinetochore | 1.000 | 0 | 4e-10 | 15 | 78 |
| GO:0000793 | condensed chromosome | 1.000 | 0 | 3e-10 | 18 | 117 |
| GO:0015630 | microtubule cytoskeleton | 1.000 | 0 | 9e-11 | 37 | 465 |
| GO:0032991 | macromolecular complex | 1.000 | 0 | 5e-11 | 109 | 2584 |
| GO:0031981 | nuclear lumen | 1.000 | 0 | 3e-11 | 69 | 1280 |
| GO:0044422 | organelle part | 1.000 | 0 | 2e-12 | 151 | 3992 |
| GO:0043228 | non-membrane-bounded organelle | 1.000 | 0 | 6e-13 | 99 | 2087 |
| GO:0043232 | intracellular non-membrane-bounded organelle | 1.000 | 0 | 6e-13 | 99 | 2087 |
| GO:0044446 | intracellular organelle part | 1.000 | 0 | 5e-13 | 151 | 3939 |
| GO:0005654 | nucleoplasm | 1.000 | 0 | 4e-13 | 56 | 821 |
| GO:0043233 | organelle lumen | 1.000 | 0 | 3e-13 | 84 | 1591 |
| GO:0031974 | membrane-enclosed lumen | 1.000 | 0 | 1e-13 | 86 | 1622 |
| GO:0070013 | intracellular organelle lumen | 1.000 | 0 | 8e-14 | 84 | 1555 |
| GO:0000775 | chromosome, centromeric region | 1.000 | 0 | 5e-14 | 22 | 118 |
| GO:0005819 | spindle | 1.000 | 0 | 7e-15 | 25 | 146 |
| GO:0044428 | nuclear part | 1.000 | 0 | 4e-15 | 89 | 1625 |
| GO:0044427 | chromosomal part | 1.000 | 0 | 3e-16 | 39 | 344 |
| GO:0005694 | chromosome | 1.000 | 0 | <2e-16 | 44 | 416 |
| GO:0043226 | organelle | 0.621 | 1 | <2e-16 | 228 | 6947 |
| GO:0043229 | intracellular organelle | 0.620 | 1 | <2e-16 | 228 | 6933 |
| GO:0005622 | intracellular | 0.748 | 1 | <2e-16 | 253 | 8358 |
| GO:0043227 | membrane-bounded organelle | 0.561 | 1 | <2e-16 | 217 | 6268 |
| GO:0005634 | nucleus | 0.346 | 1 | <2e-16 | 162 | 3864 |
| GO:0043231 | intracellular membrane-bounded organelle | 0.560 | 1 | <2e-16 | 217 | 6261 |
| GO:0044424 | intracellular part | 0.730 | 1 | <2e-16 | 251 | 8157 |

### Distribution of KEGG annotations

- Up regulated probes with KEGG annotations in above list: 1
- Down regulated probes with KEGG annotations in above list: 165
- The chip holds 7585 probes annotated to 214 pathways

|  |  |  |  |  |  |  |
| --- | --- | --- | --- | --- | --- | --- |
| **KEGG ID** | **Path Name** | **upreg.p.value** | **upreg.Int.Count** | **downreg.p.value** | **downreg.Int.Count** | **KEGG.Count** |
| 00010 | Glycolysis / Gluconeogenesis | 1 | 0 | 0.005 | 7 | 95 |
| 00020 | Citrate cycle (TCA cycle) | 1 | 0 | 1e-03 | 6 | 53 |
| 00062 | Fatty acid elongation in mitochondria | 1 | 0 | 6e-09 | 7 | 14 |
| 00071 | Fatty acid metabolism | 1 | 0 | 3e-07 | 11 | 71 |
| 00240 | Pyrimidine metabolism | 1 | 0 | 0.009 | 7 | 107 |
| 00280 | Valine, leucine and isoleucine degradation | 1 | 0 | 1e-03 | 7 | 73 |
| 00290 | Valine, leucine and isoleucine biosynthesis | 1 | 0 | 3e-04 | 4 | 16 |
| 00300 | Lysine biosynthesis | 1 | 0 | 0.003 | 2 | 4 |
| 00310 | Lysine degradation | 1 | 0 | 6e-08 | 11 | 61 |
| 00380 | Tryptophan metabolism | 1 | 0 | 5e-06 | 9 | 60 |
| 00410 | beta-Alanine metabolism | 1 | 0 | 0.007 | 4 | 36 |
| 00620 | Pyruvate metabolism | 1 | 0 | 0.008 | 5 | 57 |
| 00640 | Propanoate metabolism | 1 | 0 | 0.005 | 5 | 51 |
| 00650 | Butanoate metabolism | 1 | 0 | 3e-07 | 9 | 44 |
| 00770 | Pantothenate and CoA biosynthesis | 1 | 0 | 0.008 | 3 | 19 |
| 00903 | Limonene and pinene degradation | 1 | 0 | 3e-04 | 4 | 16 |
| 00970 | Aminoacyl-tRNA biosynthesis | 1 | 0 | 0.003 | 5 | 44 |
| 01040 | Biosynthesis of unsaturated fatty acids | 1 | 0 | 0.007 | 4 | 36 |
| 03030 | DNA replication | 1 | 0 | 5e-11 | 13 | 52 |
| 03410 | Base excision repair | 1 | 0 | 4e-06 | 8 | 44 |
| 03420 | Nucleotide excision repair | 1 | 0 | 2e-04 | 7 | 58 |
| 03430 | Mismatch repair | 1 | 0 | 6e-07 | 8 | 35 |
| 03440 | Homologous recombination | 1 | 0 | 0.002 | 5 | 43 |
| 04110 | Cell cycle | 1 | 0 | 4e-12 | 26 | 234 |
| 04114 | Oocyte meiosis | 1 | 0 | 3e-04 | 13 | 196 |
| 04120 | Ubiquitin mediated proteolysis | 1 | 0 | 2e-04 | 15 | 235 |

#99CCCC #CCCCCC #E8E8E8

Annotations from:

- Data package 'hgu133a.db' version 2.4.5 packaged on 2010-09-23 21:50:14 UTC; mcarlson
- Data package 'GO.db' version 2.4.5 packaged on 2010-09-23 21:49:10 UTC; mcarlson
- Data package 'KEGG.db' version 2.4.5 packaged on 2010-09-23 22:03:46 UTC; mcarlson
