# Supplementary material for: Global gene expression changes of in vitro stimulated human transformed germinal centre B cells as surrogate for oncogenic pathway activation in individual aggressive B cell lymphomas
Source: Cell Commun Signal. 2012 Dec 20;10:43. doi: 10.1186/1478-811X-10-43 (PMC3566944; doi:10.1186/1478-811X-10-43)
Supplement: Additional file 9 — Supplemental 2. Geneset enrichment Analysis identifying enriched pathways in differentially expressed genes. [file 1478-811X-10-43-S9.zip › supplementalFile2_GO_AnalysenLIMMA/BCR.2_dn.html]

- 296 unique Entrez Gene IDs considered
- on chip with 22283 probesets

- Molecular function
- Biological process
- Cellular component
- Pathways (KEGG)

### Molecular Function

- 10870 Entrez Gene IDs have annotations in category 'MF'
- 265 of these are in the above list
- upreg means upregulated in group BCR\_regulated.2 and downreg means downregulated in group BCR\_regulated.2

|  |  |  |  |  |  |  |
| --- | --- | --- | --- | --- | --- | --- |
| **GO ID** | **GO Term** | **upreg. p-value** | **upreg. int. Count** | **downreg. p-value** | **downreg. int. Count** | **GO Count** |
| GO:0000166 | nucleotide binding | 4e-07 | 75 | 1.00 | 0 | 1776 |
| GO:0003924 | GTPase activity | 6e-07 | 17 | 1.00 | 0 | 167 |
| GO:0004812 | aminoacyl-tRNA ligase activity | 2e-06 | 8 | 1.00 | 0 | 36 |
| GO:0016875 | ligase activity, forming carbon-oxygen bonds | 2e-06 | 8 | 1.00 | 0 | 36 |
| GO:0016876 | ligase activity, forming aminoacyl-tRNA and related compounds | 2e-06 | 8 | 1.00 | 0 | 36 |
| GO:0032553 | ribonucleotide binding | 1e-05 | 61 | 1.00 | 0 | 1460 |
| GO:0032555 | purine ribonucleotide binding | 1e-05 | 61 | 1.00 | 0 | 1460 |
| GO:0017076 | purine nucleotide binding | 1e-05 | 63 | 1.00 | 0 | 1528 |
| GO:0005525 | GTP binding | 1e-05 | 20 | 1.00 | 0 | 277 |
| GO:0019001 | guanyl nucleotide binding | 2e-05 | 20 | 1.00 | 0 | 289 |
| GO:0032561 | guanyl ribonucleotide binding | 2e-05 | 20 | 1.00 | 0 | 289 |
| GO:0016874 | ligase activity | 1e-04 | 20 | 1.00 | 0 | 325 |
| GO:0005515 | protein binding | 1e-04 | 188 | 0.60 | 1 | 6561 |
| GO:0017111 | nucleoside-triphosphatase activity | 2e-04 | 29 | 1.00 | 0 | 584 |
| GO:0016462 | pyrophosphatase activity | 4e-04 | 29 | 1.00 | 0 | 608 |
| GO:0016818 | hydrolase activity, acting on acid anhydrides, in phosphorus-containing anhydrides | 4e-04 | 29 | 1.00 | 0 | 611 |
| GO:0016817 | hydrolase activity, acting on acid anhydrides | 4e-04 | 29 | 1.00 | 0 | 613 |
| GO:0004906 | interferon-gamma receptor activity | 6e-04 | 2 | 1.00 | 0 | 2 |
| GO:0005488 | binding | 0.001 | 239 | 0.84 | 1 | 9129 |
| GO:0008135 | translation factor activity, nucleic acid binding | 0.001 | 7 | 1.00 | 0 | 69 |
| GO:0004067 | asparaginase activity | 0.002 | 2 | 1.00 | 0 | 3 |
| GO:0004828 | serine-tRNA ligase activity | 0.002 | 2 | 1.00 | 0 | 3 |
| GO:0019964 | interferon-gamma binding | 0.002 | 2 | 1.00 | 0 | 3 |
| GO:0004563 | beta-N-acetylhexosaminidase activity | 0.003 | 2 | 1.00 | 0 | 4 |
| GO:0003743 | translation initiation factor activity | 0.004 | 5 | 1.00 | 0 | 44 |
| GO:0030170 | pyridoxal phosphate binding | 0.004 | 5 | 1.00 | 0 | 44 |
| GO:0070279 | vitamin B6 binding | 0.004 | 5 | 1.00 | 0 | 44 |
| GO:0019829 | cation-transporting ATPase activity | 0.004 | 4 | 1.00 | 0 | 28 |
| GO:0004904 | interferon receptor activity | 0.006 | 2 | 1.00 | 0 | 5 |
| GO:0019238 | cyclohydrolase activity | 0.006 | 2 | 1.00 | 0 | 5 |
| GO:0015175 | neutral amino acid transmembrane transporter activity | 0.006 | 3 | 1.00 | 0 | 16 |
| GO:0046961 | proton-transporting ATPase activity, rotational mechanism | 0.006 | 3 | 1.00 | 0 | 16 |
| GO:0004843 | ubiquitin-specific protease activity | 0.006 | 4 | 1.00 | 0 | 31 |
| GO:0046982 | protein heterodimerization activity | 0.006 | 11 | 1.00 | 0 | 189 |
| GO:0046983 | protein dimerization activity | 0.007 | 22 | 1.00 | 0 | 515 |
| GO:0019783 | small conjugating protein-specific protease activity | 0.008 | 4 | 1.00 | 0 | 33 |
| GO:0004372 | glycine hydroxymethyltransferase activity | 0.008 | 2 | 1.00 | 0 | 6 |
| GO:0016308 | 1-phosphatidylinositol-4-phosphate 5-kinase activity | 0.008 | 2 | 1.00 | 0 | 6 |
| GO:0019961 | interferon binding | 0.008 | 2 | 1.00 | 0 | 6 |
| GO:0031996 | thioesterase binding | 0.008 | 2 | 1.00 | 0 | 6 |
| GO:0051787 | misfolded protein binding | 0.008 | 2 | 1.00 | 0 | 6 |
| GO:0001883 | purine nucleoside binding | 0.009 | 45 | 1.00 | 0 | 1301 |
| GO:0001882 | nucleoside binding | 0.010 | 45 | 1.00 | 0 | 1311 |

### Biological Process

- 10392 Entrez Gene IDs have annotations in category 'BP'
- 254 of these are in the above list
- upreg means upregulated in group BCR\_regulated.2 and downreg means downregulated in group BCR\_regulated.2

|  |  |  |  |  |  |  |
| --- | --- | --- | --- | --- | --- | --- |
| **GO ID** | **GO Term** | **upreg. p-value** | **upreg. int. Count** | **downreg. p-value** | **downreg. int. Count** | **GO Count** |
| GO:0006418 | tRNA aminoacylation for protein translation | 2e-06 | 8 | 1.000 | 0 | 36 |
| GO:0043038 | amino acid activation | 2e-06 | 8 | 1.000 | 0 | 36 |
| GO:0043039 | tRNA aminoacylation | 2e-06 | 8 | 1.000 | 0 | 36 |
| GO:0006511 | ubiquitin-dependent protein catabolic process | 2e-05 | 18 | 1.000 | 0 | 239 |
| GO:0019941 | modification-dependent protein catabolic process | 3e-05 | 18 | 1.000 | 0 | 245 |
| GO:0043632 | modification-dependent macromolecule catabolic process | 3e-05 | 18 | 1.000 | 0 | 245 |
| GO:0051603 | proteolysis involved in cellular protein catabolic process | 7e-05 | 19 | 1.000 | 0 | 285 |
| GO:0044257 | cellular protein catabolic process | 7e-05 | 19 | 1.000 | 0 | 287 |
| GO:0016192 | vesicle-mediated transport | 2e-04 | 28 | 1.000 | 0 | 550 |
| GO:0051789 | response to protein stimulus | 2e-04 | 10 | 1.000 | 0 | 103 |
| GO:0006986 | response to unfolded protein | 3e-04 | 7 | 1.000 | 0 | 54 |
| GO:0030163 | protein catabolic process | 4e-04 | 19 | 1.000 | 0 | 329 |
| GO:0015988 | energy coupled proton transport, against electrochemical gradient | 5e-04 | 3 | 1.000 | 0 | 7 |
| GO:0015991 | ATP hydrolysis coupled proton transport | 5e-04 | 3 | 1.000 | 0 | 7 |
| GO:0044248 | cellular catabolic process | 5e-04 | 36 | 1.000 | 0 | 831 |
| GO:0043161 | proteasomal ubiquitin-dependent protein catabolic process | 8e-04 | 11 | 1.000 | 0 | 144 |
| GO:0044265 | cellular macromolecule catabolic process | 8e-04 | 21 | 1.000 | 0 | 401 |
| GO:0010498 | proteasomal protein catabolic process | 1e-03 | 11 | 1.000 | 0 | 148 |
| GO:0006399 | tRNA metabolic process | 0.001 | 8 | 1.000 | 0 | 88 |
| GO:0006434 | seryl-tRNA aminoacylation | 0.002 | 2 | 1.000 | 0 | 3 |
| GO:0006914 | autophagy | 0.002 | 5 | 1.000 | 0 | 39 |
| GO:0042147 | retrograde transport, endosome to Golgi | 0.003 | 3 | 1.000 | 0 | 12 |
| GO:0008104 | protein localization | 0.003 | 34 | 1.000 | 0 | 854 |
| GO:0015031 | protein transport | 0.003 | 30 | 1.000 | 0 | 725 |
| GO:0009057 | macromolecule catabolic process | 0.003 | 22 | 1.000 | 0 | 478 |
| GO:0006513 | protein monoubiquitination | 0.003 | 3 | 1.000 | 0 | 13 |
| GO:0007035 | vacuolar acidification | 0.003 | 2 | 1.000 | 0 | 4 |
| GO:0007264 | small GTPase mediated signal transduction | 0.004 | 17 | 1.000 | 0 | 339 |
| GO:0045184 | establishment of protein localization | 0.004 | 30 | 1.000 | 0 | 740 |
| GO:0031325 | positive regulation of cellular metabolic process | 0.004 | 34 | 1.000 | 0 | 875 |
| GO:0015804 | neutral amino acid transport | 0.004 | 3 | 1.000 | 0 | 14 |
| GO:0034976 | response to endoplasmic reticulum stress | 0.005 | 4 | 1.000 | 0 | 29 |
| GO:0051382 | kinetochore assembly | 0.006 | 2 | 1.000 | 0 | 5 |
| GO:0051383 | kinetochore organization | 0.006 | 2 | 1.000 | 0 | 5 |
| GO:0034660 | ncRNA metabolic process | 0.006 | 11 | 1.000 | 0 | 186 |
| GO:0006897 | endocytosis | 0.006 | 13 | 1.000 | 0 | 241 |
| GO:0010324 | membrane invagination | 0.006 | 13 | 1.000 | 0 | 241 |
| GO:0006412 | translation | 0.006 | 16 | 1.000 | 0 | 327 |
| GO:0033036 | macromolecule localization | 0.006 | 38 | 1.000 | 0 | 1034 |
| GO:0030968 | endoplasmic reticulum unfolded protein response | 0.008 | 3 | 1.000 | 0 | 17 |
| GO:0034620 | cellular response to unfolded protein | 0.008 | 3 | 1.000 | 0 | 17 |
| GO:0046488 | phosphatidylinositol metabolic process | 0.008 | 3 | 1.000 | 0 | 17 |
| GO:0009056 | catabolic process | 0.008 | 37 | 1.000 | 0 | 1017 |
| GO:0046653 | tetrahydrofolate metabolic process | 0.008 | 2 | 1.000 | 0 | 6 |
| GO:0006520 | cellular amino acid metabolic process | 0.009 | 11 | 1.000 | 0 | 197 |
| GO:0016071 | mRNA metabolic process | 0.009 | 15 | 1.000 | 0 | 310 |
| GO:0032535 | regulation of cellular component size | 0.009 | 15 | 1.000 | 0 | 312 |
| GO:0009893 | positive regulation of metabolic process | 0.010 | 34 | 1.000 | 0 | 924 |
| GO:0010604 | positive regulation of macromolecule metabolic process | 0.010 | 32 | 1.000 | 0 | 858 |

### Cellular Component

- 11181 Entrez Gene IDs have annotations in category 'CC'
- 275 of these are in the above list
- upreg means upregulated in group BCR\_regulated.2 and downreg means downregulated in group BCR\_regulated.2

|  |  |  |  |  |  |  |
| --- | --- | --- | --- | --- | --- | --- |
| **GO ID** | **GO Term** | **upreg. p-value** | **upreg. int. Count** | **downreg. p-value** | **downreg. int. Count** | **GO Count** |
| GO:0005773 | vacuole | 1e-05 | 18 | 1.000 | 0 | 229 |
| GO:0043231 | intracellular membrane-bounded organelle | 3e-05 | 186 | 0.560 | 1 | 6261 |
| GO:0043227 | membrane-bounded organelle | 3e-05 | 186 | 0.561 | 1 | 6268 |
| GO:0010008 | endosome membrane | 5e-05 | 14 | 1.000 | 0 | 163 |
| GO:0044440 | endosomal part | 5e-05 | 14 | 1.000 | 0 | 163 |
| GO:0005622 | intracellular | 8e-05 | 231 | 0.748 | 1 | 8358 |
| GO:0000323 | lytic vacuole | 8e-05 | 15 | 1.000 | 0 | 193 |
| GO:0005764 | lysosome | 8e-05 | 15 | 1.000 | 0 | 193 |
| GO:0044424 | intracellular part | 1e-04 | 226 | 0.730 | 1 | 8157 |
| GO:0005768 | endosome | 3e-04 | 19 | 1.000 | 0 | 313 |
| GO:0042470 | melanosome | 3e-04 | 9 | 1.000 | 0 | 89 |
| GO:0048770 | pigment granule | 3e-04 | 9 | 1.000 | 0 | 89 |
| GO:0005737 | cytoplasm | 5e-04 | 174 | 0.536 | 1 | 5993 |
| GO:0005774 | vacuolar membrane | 6e-04 | 9 | 1.000 | 0 | 96 |
| GO:0044437 | vacuolar part | 8e-04 | 9 | 1.000 | 0 | 100 |
| GO:0005770 | late endosome | 9e-04 | 8 | 1.000 | 0 | 82 |
| GO:0044444 | cytoplasmic part | 0.001 | 125 | 1.000 | 0 | 4110 |
| GO:0005850 | eukaryotic translation initiation factor 2 complex | 0.002 | 2 | 1.000 | 0 | 3 |
| GO:0031982 | vesicle | 0.002 | 27 | 1.000 | 0 | 616 |
| GO:0031410 | cytoplasmic vesicle | 0.002 | 26 | 1.000 | 0 | 586 |
| GO:0042588 | zymogen granule | 0.003 | 3 | 1.000 | 0 | 12 |
| GO:0043229 | intracellular organelle | 0.003 | 192 | 0.620 | 1 | 6933 |
| GO:0043226 | organelle | 0.003 | 192 | 0.621 | 1 | 6947 |
| GO:0031902 | late endosome membrane | 0.003 | 5 | 1.000 | 0 | 42 |
| GO:0005681 | spliceosomal complex | 0.007 | 8 | 1.000 | 0 | 113 |
| GO:0005765 | lysosomal membrane | 0.007 | 6 | 1.000 | 0 | 70 |
| GO:0030530 | heterogeneous nuclear ribonucleoprotein complex | 0.008 | 3 | 1.000 | 0 | 17 |

### Distribution of KEGG annotations

- Up regulated probes with KEGG annotations in above list: 222
- Down regulated probes with KEGG annotations in above list: 0
- The chip holds 7585 probes annotated to 214 pathways

|  |  |  |  |  |  |  |
| --- | --- | --- | --- | --- | --- | --- |
| **KEGG ID** | **Path Name** | **upreg.p.value** | **upreg.Int.Count** | **downreg.p.value** | **downreg.Int.Count** | **KEGG.Count** |
| 00970 | Aminoacyl-tRNA biosynthesis | 2e-10 | 13 | 1 | 0 | 44 |
| 04142 | Lysosome | 2e-07 | 22 | 1 | 0 | 212 |
| 04145 | Phagosome | 1e-06 | 24 | 1 | 0 | 272 |
| 00670 | One carbon pool by folate | 9e-06 | 7 | 1 | 0 | 27 |
| 00511 | Other glycan degradation | 1e-05 | 6 | 1 | 0 | 19 |
| 04966 | Collecting duct acid secretion | 8e-05 | 7 | 1 | 0 | 37 |
| 05120 | Epithelial cell signaling in Helicobacter pylori infection | 2e-04 | 12 | 1 | 0 | 121 |
| 04144 | Endocytosis | 4e-04 | 23 | 1 | 0 | 365 |
| 04710 | Circadian rhythm - mammal | 7e-04 | 6 | 1 | 0 | 38 |
| 00750 | Vitamin B6 metabolism | 8e-04 | 3 | 1 | 0 | 7 |
| 04141 | Protein processing in endoplasmic reticulum | 8e-04 | 18 | 1 | 0 | 268 |
| 05130 | Pathogenic Escherichia coli infection | 0.001 | 10 | 1 | 0 | 107 |
| 04340 | Hedgehog signaling pathway | 0.001 | 8 | 1 | 0 | 74 |
| 04120 | Ubiquitin mediated proteolysis | 0.001 | 16 | 1 | 0 | 235 |
| 00900 | Terpenoid backbone biosynthesis | 0.003 | 4 | 1 | 0 | 21 |
| 03040 | Spliceosome | 0.006 | 13 | 1 | 0 | 203 |
| 04612 | Antigen processing and presentation | 0.008 | 10 | 1 | 0 | 141 |

#99CCCC #CCCCCC #E8E8E8

Annotations from:

- Data package 'hgu133a.db' version 2.4.5 packaged on 2010-09-23 21:50:14 UTC; mcarlson
- Data package 'GO.db' version 2.4.5 packaged on 2010-09-23 21:49:10 UTC; mcarlson
- Data package 'KEGG.db' version 2.4.5 packaged on 2010-09-23 22:03:46 UTC; mcarlson
