# Supplementary material for: Global gene expression changes of in vitro stimulated human transformed germinal centre B cells as surrogate for oncogenic pathway activation in individual aggressive B cell lymphomas
Source: Cell Commun Signal. 2012 Dec 20;10:43. doi: 10.1186/1478-811X-10-43 (PMC3566944; doi:10.1186/1478-811X-10-43)
Supplement: Additional file 9 — Supplemental 2. Geneset enrichment Analysis identifying enriched pathways in differentially expressed genes. [file 1478-811X-10-43-S9.zip › supplementalFile2_GO_AnalysenLIMMA/CD40.1_up.html]

- 297 unique Entrez Gene IDs considered
- on chip with 22283 probesets

- Molecular function
- Biological process
- Cellular component
- Pathways (KEGG)

### Molecular Function

- 10870 Entrez Gene IDs have annotations in category 'MF'
- 249 of these are in the above list
- upreg means upregulated in group CD40\_regulated.1 and downreg means downregulated in group CD40\_regulated.1

|  |  |  |  |  |  |  |
| --- | --- | --- | --- | --- | --- | --- |
| **GO ID** | **GO Term** | **upreg. p-value** | **upreg. int. Count** | **downreg. p-value** | **downreg. int. Count** | **GO Count** |
| GO:0003723 | RNA binding | 4e-10 | 38 | 1.000 | 0 | 574 |
| GO:0000166 | nucleotide binding | 9e-09 | 73 | 0.454 | 3 | 1776 |
| GO:0017076 | purine nucleotide binding | 7e-06 | 58 | 0.354 | 3 | 1528 |
| GO:0032393 | MHC class I receptor activity | 8e-06 | 5 | 0.019 | 1 | 14 |
| GO:0030554 | adenyl nucleotide binding | 3e-05 | 49 | 0.536 | 2 | 1268 |
| GO:0032553 | ribonucleotide binding | 3e-05 | 54 | 0.327 | 3 | 1460 |
| GO:0032555 | purine ribonucleotide binding | 3e-05 | 54 | 0.327 | 3 | 1460 |
| GO:0001883 | purine nucleoside binding | 6e-05 | 49 | 0.551 | 2 | 1301 |
| GO:0001882 | nucleoside binding | 7e-05 | 49 | 0.555 | 2 | 1311 |
| GO:0005524 | ATP binding | 1e-04 | 45 | 0.497 | 2 | 1183 |
| GO:0032559 | adenyl ribonucleotide binding | 1e-04 | 45 | 0.506 | 2 | 1202 |
| GO:0008026 | ATP-dependent helicase activity | 4e-04 | 8 | 1.000 | 0 | 84 |
| GO:0070035 | purine NTP-dependent helicase activity | 4e-04 | 8 | 1.000 | 0 | 84 |
| GO:0017111 | nucleoside-triphosphatase activity | 8e-04 | 25 | 0.191 | 2 | 584 |
| GO:0004386 | helicase activity | 9e-04 | 9 | 1.000 | 0 | 116 |
| GO:0004812 | aminoacyl-tRNA ligase activity | 1e-03 | 5 | 1.000 | 0 | 36 |
| GO:0016875 | ligase activity, forming carbon-oxygen bonds | 1e-03 | 5 | 1.000 | 0 | 36 |
| GO:0016876 | ligase activity, forming aminoacyl-tRNA and related compounds | 1e-03 | 5 | 1.000 | 0 | 36 |
| GO:0003676 | nucleic acid binding | 0.001 | 64 | 0.810 | 2 | 2069 |
| GO:0008094 | DNA-dependent ATPase activity | 0.001 | 6 | 1.000 | 0 | 55 |
| GO:0016887 | ATPase activity | 0.001 | 15 | 0.328 | 1 | 284 |
| GO:0016462 | pyrophosphatase activity | 0.001 | 25 | 0.203 | 2 | 608 |
| GO:0016818 | hydrolase activity, acting on acid anhydrides, in phosphorus-containing anhydrides | 0.001 | 25 | 0.205 | 2 | 611 |
| GO:0016817 | hydrolase activity, acting on acid anhydrides | 0.002 | 25 | 0.206 | 2 | 613 |
| GO:0008320 | protein transmembrane transporter activity | 0.002 | 3 | 1.000 | 0 | 13 |
| GO:0022884 | macromolecule transmembrane transporter activity | 0.002 | 3 | 1.000 | 0 | 13 |
| GO:0016889 | endodeoxyribonuclease activity, producing 3'-phosphomonoesters | 0.003 | 2 | 1.000 | 0 | 4 |
| GO:0000049 | tRNA binding | 0.004 | 3 | 1.000 | 0 | 15 |
| GO:0004518 | nuclease activity | 0.004 | 8 | 1.000 | 0 | 119 |
| GO:0042623 | ATPase activity, coupled | 0.004 | 12 | 0.277 | 1 | 232 |
| GO:0046934 | phosphatidylinositol-4,5-bisphosphate 3-kinase activity | 0.004 | 2 | 1.000 | 0 | 5 |
| GO:0048256 | flap endonuclease activity | 0.004 | 2 | 1.000 | 0 | 5 |
| GO:0003730 | mRNA 3'-UTR binding | 0.004 | 3 | 1.000 | 0 | 16 |
| GO:0016874 | ligase activity | 0.005 | 15 | 1.000 | 0 | 325 |
| GO:0004519 | endonuclease activity | 0.005 | 6 | 1.000 | 0 | 73 |
| GO:0008409 | 5'-3' exonuclease activity | 0.007 | 2 | 1.000 | 0 | 6 |
| GO:0004520 | endodeoxyribonuclease activity | 0.009 | 3 | 1.000 | 0 | 20 |
| GO:0003678 | DNA helicase activity | 0.010 | 4 | 1.000 | 0 | 39 |
| GO:0022891 | substrate-specific transmembrane transporter activity | 0.998 | 5 | 0.009 | 4 | 629 |
| GO:0004871 | signal transducer activity | 1.000 | 9 | 0.008 | 6 | 1416 |
| GO:0060089 | molecular transducer activity | 1.000 | 9 | 0.008 | 6 | 1416 |
| GO:0015267 | channel activity | 1.000 | 0 | 0.008 | 3 | 308 |
| GO:0022803 | passive transmembrane transporter activity | 1.000 | 0 | 0.008 | 3 | 308 |
| GO:0022838 | substrate-specific channel activity | 1.000 | 0 | 0.007 | 3 | 295 |
| GO:0005216 | ion channel activity | 1.000 | 0 | 0.006 | 3 | 285 |
| GO:0015075 | ion transmembrane transporter activity | 1.000 | 1 | 0.006 | 4 | 546 |

### Biological Process

- 10392 Entrez Gene IDs have annotations in category 'BP'
- 240 of these are in the above list
- upreg means upregulated in group CD40\_regulated.1 and downreg means downregulated in group CD40\_regulated.1

|  |  |  |  |  |  |  |
| --- | --- | --- | --- | --- | --- | --- |
| **GO ID** | **GO Term** | **upreg. p-value** | **upreg. int. Count** | **downreg. p-value** | **downreg. int. Count** | **GO Count** |
| GO:0006396 | RNA processing | 9e-09 | 32 | 1.000 | 0 | 475 |
| GO:0034660 | ncRNA metabolic process | 5e-07 | 17 | 1.000 | 0 | 186 |
| GO:0006397 | mRNA processing | 7e-07 | 20 | 1.000 | 0 | 257 |
| GO:0016071 | mRNA metabolic process | 9e-07 | 22 | 1.000 | 0 | 310 |
| GO:0042254 | ribosome biogenesis | 1e-06 | 13 | 1.000 | 0 | 114 |
| GO:0022613 | ribonucleoprotein complex biogenesis | 4e-06 | 15 | 1.000 | 0 | 170 |
| GO:0008380 | RNA splicing | 8e-06 | 18 | 1.000 | 0 | 251 |
| GO:0006626 | protein targeting to mitochondrion | 9e-06 | 6 | 1.000 | 0 | 24 |
| GO:0070585 | protein localization in mitochondrion | 9e-06 | 6 | 1.000 | 0 | 24 |
| GO:0006364 | rRNA processing | 2e-05 | 10 | 1.000 | 0 | 86 |
| GO:0016072 | rRNA metabolic process | 2e-05 | 10 | 1.000 | 0 | 88 |
| GO:0002474 | antigen processing and presentation of peptide antigen via MHC class I | 2e-05 | 5 | 1.000 | 0 | 17 |
| GO:0007005 | mitochondrion organization | 8e-05 | 11 | 0.165 | 1 | 124 |
| GO:0006412 | translation | 9e-05 | 19 | 0.381 | 1 | 327 |
| GO:0034470 | ncRNA processing | 2e-04 | 12 | 1.000 | 0 | 157 |
| GO:0048002 | antigen processing and presentation of peptide antigen | 2e-04 | 5 | 1.000 | 0 | 26 |
| GO:0044260 | cellular macromolecule metabolic process | 6e-04 | 118 | 0.646 | 6 | 4325 |
| GO:0006399 | tRNA metabolic process | 6e-04 | 8 | 1.000 | 0 | 88 |
| GO:0010608 | posttranscriptional regulation of gene expression | 6e-04 | 13 | 1.000 | 0 | 210 |
| GO:0019882 | antigen processing and presentation | 9e-04 | 6 | 1.000 | 0 | 52 |
| GO:0006418 | tRNA aminoacylation for protein translation | 1e-03 | 5 | 1.000 | 0 | 36 |
| GO:0043038 | amino acid activation | 1e-03 | 5 | 1.000 | 0 | 36 |
| GO:0043039 | tRNA aminoacylation | 1e-03 | 5 | 1.000 | 0 | 36 |
| GO:0006417 | regulation of translation | 0.001 | 9 | 1.000 | 0 | 121 |
| GO:0051436 | negative regulation of ubiquitin-protein ligase activity involved in mitotic cell cycle | 0.001 | 6 | 1.000 | 0 | 57 |
| GO:0031145 | anaphase-promoting complex-dependent proteasomal ubiquitin-dependent protein catabolic process | 0.002 | 6 | 1.000 | 0 | 59 |
| GO:0051437 | positive regulation of ubiquitin-protein ligase activity involved in mitotic cell cycle | 0.002 | 6 | 1.000 | 0 | 60 |
| GO:0006839 | mitochondrial transport | 0.002 | 6 | 1.000 | 0 | 61 |
| GO:0051352 | negative regulation of ligase activity | 0.002 | 6 | 1.000 | 0 | 61 |
| GO:0051444 | negative regulation of ubiquitin-protein ligase activity | 0.002 | 6 | 1.000 | 0 | 61 |
| GO:0051439 | regulation of ubiquitin-protein ligase activity involved in mitotic cell cycle | 0.002 | 6 | 1.000 | 0 | 63 |
| GO:0007008 | outer mitochondrial membrane organization | 0.003 | 2 | 1.000 | 0 | 4 |
| GO:0009129 | pyrimidine nucleoside monophosphate metabolic process | 0.003 | 2 | 1.000 | 0 | 4 |
| GO:0009130 | pyrimidine nucleoside monophosphate biosynthetic process | 0.003 | 2 | 1.000 | 0 | 4 |
| GO:0031848 | protection from non-homologous end joining at telomere | 0.003 | 2 | 1.000 | 0 | 4 |
| GO:0043247 | telomere maintenance in response to DNA damage | 0.003 | 2 | 1.000 | 0 | 4 |
| GO:0070096 | mitochondrial outer membrane translocase complex assembly | 0.003 | 2 | 1.000 | 0 | 4 |
| GO:0031398 | positive regulation of protein ubiquitination | 0.003 | 7 | 1.000 | 0 | 87 |
| GO:0007006 | mitochondrial membrane organization | 0.003 | 4 | 1.000 | 0 | 28 |
| GO:0051443 | positive regulation of ubiquitin-protein ligase activity | 0.003 | 6 | 1.000 | 0 | 67 |
| GO:0051351 | positive regulation of ligase activity | 0.004 | 6 | 1.000 | 0 | 70 |
| GO:0016233 | telomere capping | 0.004 | 2 | 1.000 | 0 | 5 |
| GO:0045948 | positive regulation of translational initiation | 0.004 | 2 | 1.000 | 0 | 5 |
| GO:0016070 | RNA metabolic process | 0.005 | 58 | 0.800 | 2 | 1940 |
| GO:0031397 | negative regulation of protein ubiquitination | 0.005 | 6 | 1.000 | 0 | 73 |
| GO:0044419 | interspecies interaction between organisms | 0.005 | 15 | 1.000 | 0 | 330 |
| GO:0016567 | protein ubiquitination | 0.006 | 12 | 1.000 | 0 | 239 |
| GO:0051438 | regulation of ubiquitin-protein ligase activity | 0.006 | 6 | 1.000 | 0 | 76 |
| GO:0051340 | regulation of ligase activity | 0.007 | 6 | 1.000 | 0 | 79 |
| GO:0017038 | protein import | 0.008 | 9 | 0.207 | 1 | 159 |
| GO:0071806 | protein transmembrane transport | 0.008 | 9 | 0.208 | 1 | 160 |
| GO:0006446 | regulation of translational initiation | 0.008 | 4 | 1.000 | 0 | 37 |
| GO:0010467 | gene expression | 0.009 | 78 | 0.821 | 3 | 2838 |
| GO:0000082 | G1/S transition of mitotic cell cycle | 0.009 | 5 | 1.000 | 0 | 59 |
| GO:0007141 | male meiosis I | 0.009 | 2 | 1.000 | 0 | 7 |
| GO:0031396 | regulation of protein ubiquitination | 0.010 | 7 | 1.000 | 0 | 110 |
| GO:0055085 | transmembrane transport | 0.464 | 13 | 0.008 | 4 | 570 |
| GO:0007411 | axon guidance | 0.842 | 1 | 0.006 | 2 | 84 |

### Cellular Component

- 11181 Entrez Gene IDs have annotations in category 'CC'
- 261 of these are in the above list
- upreg means upregulated in group CD40\_regulated.1 and downreg means downregulated in group CD40\_regulated.1

|  |  |  |  |  |  |  |
| --- | --- | --- | --- | --- | --- | --- |
| **GO ID** | **GO Term** | **upreg. p-value** | **upreg. int. Count** | **downreg. p-value** | **downreg. int. Count** | **GO Count** |
| GO:0070013 | intracellular organelle lumen | 3e-12 | 76 | 1.000 | 0 | 1555 |
| GO:0043231 | intracellular membrane-bounded organelle | 5e-12 | 189 | 0.979 | 5 | 6261 |
| GO:0043227 | membrane-bounded organelle | 6e-12 | 189 | 0.979 | 5 | 6268 |
| GO:0031974 | membrane-enclosed lumen | 1e-11 | 77 | 1.000 | 0 | 1622 |
| GO:0043233 | organelle lumen | 1e-11 | 76 | 1.000 | 0 | 1591 |
| GO:0005622 | intracellular | 2e-09 | 221 | 1.000 | 5 | 8358 |
| GO:0005730 | nucleolus | 4e-09 | 38 | 1.000 | 0 | 604 |
| GO:0044428 | nuclear part | 4e-09 | 71 | 1.000 | 0 | 1625 |
| GO:0031981 | nuclear lumen | 7e-09 | 60 | 1.000 | 0 | 1280 |
| GO:0044424 | intracellular part | 2e-08 | 215 | 1.000 | 5 | 8157 |
| GO:0043229 | intracellular organelle | 4e-08 | 192 | 0.994 | 5 | 6933 |
| GO:0043226 | organelle | 5e-08 | 192 | 0.994 | 5 | 6947 |
| GO:0005634 | nucleus | 2e-07 | 124 | 0.985 | 2 | 3864 |
| GO:0044446 | intracellular organelle part | 6e-07 | 124 | 0.999 | 1 | 3939 |
| GO:0044422 | organelle part | 7e-07 | 125 | 0.999 | 1 | 3992 |
| GO:0044429 | mitochondrial part | 2e-06 | 28 | 1.000 | 0 | 479 |
| GO:0005739 | mitochondrion | 4e-06 | 43 | 0.739 | 1 | 956 |
| GO:0016607 | nuclear speck | 1e-05 | 11 | 1.000 | 0 | 99 |
| GO:0042611 | MHC protein complex | 2e-05 | 6 | 1.000 | 0 | 26 |
| GO:0042612 | MHC class I protein complex | 2e-05 | 5 | 1.000 | 0 | 17 |
| GO:0030529 | ribonucleoprotein complex | 3e-05 | 23 | 1.000 | 0 | 409 |
| GO:0005759 | mitochondrial matrix | 7e-05 | 14 | 1.000 | 0 | 188 |
| GO:0031980 | mitochondrial lumen | 7e-05 | 14 | 1.000 | 0 | 188 |
| GO:0005737 | cytoplasm | 2e-04 | 160 | 0.998 | 3 | 5993 |
| GO:0016604 | nuclear body | 4e-04 | 12 | 1.000 | 0 | 173 |
| GO:0032991 | macromolecular complex | 7e-04 | 79 | 0.893 | 2 | 2584 |
| GO:0005681 | spliceosomal complex | 9e-04 | 9 | 1.000 | 0 | 113 |
| GO:0031967 | organelle envelope | 0.001 | 23 | 1.000 | 0 | 529 |
| GO:0000315 | organellar large ribosomal subunit | 0.002 | 3 | 1.000 | 0 | 11 |
| GO:0005762 | mitochondrial large ribosomal subunit | 0.002 | 3 | 1.000 | 0 | 11 |
| GO:0031966 | mitochondrial membrane | 0.002 | 16 | 1.000 | 0 | 317 |
| GO:0031975 | envelope | 0.002 | 23 | 1.000 | 0 | 539 |
| GO:0000502 | proteasome complex | 0.002 | 6 | 1.000 | 0 | 59 |
| GO:0005654 | nucleoplasm | 0.002 | 31 | 1.000 | 0 | 821 |
| GO:0005740 | mitochondrial envelope | 0.003 | 16 | 1.000 | 0 | 337 |
| GO:0044444 | cytoplasmic part | 0.004 | 111 | 0.953 | 3 | 4110 |
| GO:0000145 | exocyst | 0.005 | 2 | 1.000 | 0 | 5 |
| GO:0000313 | organellar ribosome | 0.005 | 4 | 1.000 | 0 | 32 |
| GO:0005761 | mitochondrial ribosome | 0.005 | 4 | 1.000 | 0 | 32 |
| GO:0031901 | early endosome membrane | 0.006 | 4 | 1.000 | 0 | 33 |
| GO:0030530 | heterogeneous nuclear ribonucleoprotein complex | 0.006 | 3 | 1.000 | 0 | 17 |
| GO:0044455 | mitochondrial membrane part | 0.007 | 7 | 1.000 | 0 | 101 |
| GO:0044464 | cell part | 0.007 | 238 | 0.913 | 13 | 10383 |
| GO:0005623 | cell | 0.007 | 238 | 0.914 | 13 | 10384 |
| GO:0043228 | non-membrane-bounded organelle | 0.010 | 61 | 1.000 | 0 | 2087 |
| GO:0043232 | intracellular non-membrane-bounded organelle | 0.010 | 61 | 1.000 | 0 | 2087 |

### Distribution of KEGG annotations

- Up regulated probes with KEGG annotations in above list: 189
- Down regulated probes with KEGG annotations in above list: 15
- The chip holds 7585 probes annotated to 214 pathways

|  |  |  |  |  |  |  |
| --- | --- | --- | --- | --- | --- | --- |
| **KEGG ID** | **Path Name** | **upreg.p.value** | **upreg.Int.Count** | **downreg.p.value** | **downreg.Int.Count** | **KEGG.Count** |
| 04612 | Antigen processing and presentation | <2e-16 | 27 | 1.000 | 0 | 141 |
| 05330 | Allograft rejection | 1e-12 | 17 | 1.000 | 0 | 73 |
| 05332 | Graft-versus-host disease | 3e-12 | 17 | 1.000 | 0 | 76 |
| 03040 | Spliceosome | 2e-11 | 25 | 1.000 | 0 | 203 |
| 04940 | Type I diabetes mellitus | 4e-11 | 17 | 1.000 | 0 | 89 |
| 05320 | Autoimmune thyroid disease | 4e-11 | 17 | 1.000 | 0 | 89 |
| 05416 | Viral myocarditis | 4e-11 | 21 | 1.000 | 0 | 144 |
| 04650 | Natural killer cell mediated cytotoxicity | 7e-10 | 24 | 1.000 | 0 | 218 |
| 03050 | Proteasome | 3e-06 | 10 | 1.000 | 0 | 64 |
| 04514 | Cell adhesion molecules (CAMs) | 8e-06 | 19 | 1.000 | 0 | 241 |
| 04145 | Phagosome | 1e-05 | 20 | 1.000 | 0 | 272 |
| 03440 | Homologous recombination | 8e-05 | 7 | 1.000 | 0 | 43 |
| 04150 | mTOR signaling pathway | 8e-05 | 10 | 1.000 | 0 | 91 |
| 00970 | Aminoacyl-tRNA biosynthesis | 9e-05 | 7 | 1.000 | 0 | 44 |
| 04144 | Endocytosis | 1e-04 | 22 | 1.000 | 0 | 365 |
| 00240 | Pyrimidine metabolism | 0.001 | 9 | 1.000 | 0 | 107 |
| 04662 | B cell receptor signaling pathway | 0.001 | 10 | 1.000 | 0 | 130 |
| 04910 | Insulin signaling pathway | 0.003 | 14 | 1.000 | 0 | 243 |
| 04370 | VEGF signaling pathway | 0.006 | 9 | 1.000 | 0 | 132 |
| 04210 | Apoptosis | 0.007 | 10 | 1.000 | 0 | 163 |
| 03030 | DNA replication | 0.009 | 5 | 1.000 | 0 | 52 |
| 02010 | ABC transporters | 1.000 | 0 | 0.006 | 2 | 60 |
| 04080 | Neuroactive ligand-receptor interaction | 1.000 | 0 | 6e-05 | 6 | 388 |
| 04060 | Cytokine-cytokine receptor interaction | 1.000 | 1 | 0.005 | 4 | 374 |
| 04360 | Axon guidance | 0.609 | 5 | 0.008 | 3 | 211 |
| 04630 | Jak-STAT signaling pathway | 0.267 | 8 | 0.001 | 4 | 245 |
| 04960 | Aldosterone-regulated sodium reabsorption | 0.093 | 4 | 0.008 | 2 | 69 |

#99CCCC #CCCCCC #E8E8E8

Annotations from:

- Data package 'hgu133a.db' version 2.4.5 packaged on 2010-09-23 21:50:14 UTC; mcarlson
- Data package 'GO.db' version 2.4.5 packaged on 2010-09-23 21:49:10 UTC; mcarlson
- Data package 'KEGG.db' version 2.4.5 packaged on 2010-09-23 22:03:46 UTC; mcarlson
