# Supplementary material for: Global gene expression changes of in vitro stimulated human transformed germinal centre B cells as surrogate for oncogenic pathway activation in individual aggressive B cell lymphomas
Source: Cell Commun Signal. 2012 Dec 20;10:43. doi: 10.1186/1478-811X-10-43 (PMC3566944; doi:10.1186/1478-811X-10-43)
Supplement: Additional file 9 — Supplemental 2. Geneset enrichment Analysis identifying enriched pathways in differentially expressed genes. [file 1478-811X-10-43-S9.zip › supplementalFile2_GO_AnalysenLIMMA/CD40.2_dn.html]

- 32 unique Entrez Gene IDs considered
- on chip with 22283 probesets

- Molecular function
- Biological process
- Cellular component
- Pathways (KEGG)

### Molecular Function

- no worthwhile MF annotations found

### Biological Process

- 10392 Entrez Gene IDs have annotations in category 'BP'
- of these are in the above list
- upreg means upregulated in group CD40\_regulated.2 and downreg means downregulated in group CD40\_regulated.2

|  |  |  |  |  |  |  |
| --- | --- | --- | --- | --- | --- | --- |
| **GO ID** | **GO Term** | **upreg. p-value** | **upreg. int. Count** | **downreg. p-value** | **downreg. int. Count** | **GO Count** |
| GO:0006813 | potassium ion transport | 0.003 | 3 | 1 | 0 | 111 |
| GO:0007588 | excretion | 0.008 | 2 | 1 | 0 | 54 |

### Cellular Component

- no worthwhile CC annotations found

### Distribution of KEGG annotations

- Up regulated probes with KEGG annotations in above list: 5
- Down regulated probes with KEGG annotations in above list: 0
- The chip holds 7585 probes annotated to 214 pathways

|  |  |  |  |  |  |  |
| --- | --- | --- | --- | --- | --- | --- |
| **KEGG ID** | **Path Name** | **upreg.p.value** | **upreg.Int.Count** | **downreg.p.value** | **downreg.Int.Count** | **KEGG.Count** |
| 02010 | ABC transporters | 6e-04 | 2 | 1 | 0 | 60 |
| 04146 | Peroxisome | 0.002 | 2 | 1 | 0 | 113 |

#99CCCC #CCCCCC #E8E8E8

Annotations from:

- Data package 'hgu133a.db' version 2.4.5 packaged on 2010-09-23 21:50:14 UTC; mcarlson
- Data package 'GO.db' version 2.4.5 packaged on 2010-09-23 21:49:10 UTC; mcarlson
- Data package 'KEGG.db' version 2.4.5 packaged on 2010-09-23 22:03:46 UTC; mcarlson
