# Supplementary material for: Global gene expression changes of in vitro stimulated human transformed germinal centre B cells as surrogate for oncogenic pathway activation in individual aggressive B cell lymphomas
Source: Cell Commun Signal. 2012 Dec 20;10:43. doi: 10.1186/1478-811X-10-43 (PMC3566944; doi:10.1186/1478-811X-10-43)
Supplement: Additional file 9 — Supplemental 2. Geneset enrichment Analysis identifying enriched pathways in differentially expressed genes. [file 1478-811X-10-43-S9.zip › supplementalFile2_GO_AnalysenLIMMA/IL21.1_up.html]

- 149 unique Entrez Gene IDs considered
- on chip with 22283 probesets

- Molecular function
- Biological process
- Cellular component
- Pathways (KEGG)

### Molecular Function

- Entrez Gene IDs have annotations in category 'MF'
- of these are in the above list
- upreg means upregulated in group IL21\_regulated.1 and downreg means downregulated in group IL21\_regulated.1

|  |  |  |  |  |  |  |
| --- | --- | --- | --- | --- | --- | --- |
| **GO ID** | **GO Term** | **upreg. p-value** | **upreg. int. Count** | **downreg. p-value** | **downreg. int. Count** | **GO Count** |
| GO:0008135 | translation factor activity, nucleic acid binding | 1 | 0 | 0.007 | 4 | 69 |
| GO:0000166 | nucleotide binding | 1 | 0 | 0.007 | 31 | 1776 |
| GO:0003729 | mRNA binding | 1 | 0 | 0.005 | 4 | 62 |
| GO:0000049 | tRNA binding | 1 | 0 | 6e-04 | 3 | 15 |
| GO:0003676 | nucleic acid binding | 1 | 0 | 9e-05 | 41 | 2069 |
| GO:0003723 | RNA binding | 1 | 0 | 8e-08 | 23 | 574 |

### Biological Process

- Entrez Gene IDs have annotations in category 'BP'
- of these are in the above list
- upreg means upregulated in group IL21\_regulated.1 and downreg means downregulated in group IL21\_regulated.1

|  |  |  |  |  |  |  |
| --- | --- | --- | --- | --- | --- | --- |
| **GO ID** | **GO Term** | **upreg. p-value** | **upreg. int. Count** | **downreg. p-value** | **downreg. int. Count** | **GO Count** |
| GO:0006555 | methionine metabolic process | 1 | 0 | 0.009 | 2 | 12 |
| GO:0044257 | cellular protein catabolic process | 1 | 0 | 0.008 | 9 | 287 |
| GO:0051603 | proteolysis involved in cellular protein catabolic process | 1 | 0 | 0.007 | 9 | 285 |
| GO:0015931 | nucleobase, nucleoside, nucleotide and nucleic acid transport | 1 | 0 | 0.007 | 5 | 101 |
| GO:0030163 | protein catabolic process | 1 | 0 | 0.006 | 10 | 329 |
| GO:0006457 | protein folding | 1 | 0 | 0.006 | 6 | 135 |
| GO:0000375 | RNA splicing, via transesterification reactions | 1 | 0 | 0.005 | 5 | 94 |
| GO:0000278 | mitotic cell cycle | 1 | 0 | 0.005 | 12 | 423 |
| GO:0006403 | RNA localization | 1 | 0 | 0.005 | 5 | 90 |
| GO:0050657 | nucleic acid transport | 1 | 0 | 0.004 | 5 | 89 |
| GO:0050658 | RNA transport | 1 | 0 | 0.004 | 5 | 89 |
| GO:0051236 | establishment of RNA localization | 1 | 0 | 0.004 | 5 | 89 |
| GO:0016072 | rRNA metabolic process | 1 | 0 | 0.004 | 5 | 88 |
| GO:0009086 | methionine biosynthetic process | 1 | 0 | 0.004 | 2 | 8 |
| GO:0010608 | posttranscriptional regulation of gene expression | 1 | 0 | 0.004 | 8 | 210 |
| GO:0006364 | rRNA processing | 1 | 0 | 0.004 | 5 | 86 |
| GO:0006807 | nitrogen compound metabolic process | 1 | 0 | 0.003 | 58 | 3573 |
| GO:0000096 | sulfur amino acid metabolic process | 1 | 0 | 0.003 | 3 | 24 |
| GO:0019941 | modification-dependent protein catabolic process | 1 | 0 | 0.003 | 9 | 245 |
| GO:0043632 | modification-dependent macromolecule catabolic process | 1 | 0 | 0.003 | 9 | 245 |
| GO:0006139 | nucleobase, nucleoside, nucleotide and nucleic acid metabolic process | 1 | 0 | 0.003 | 54 | 3229 |
| GO:0006511 | ubiquitin-dependent protein catabolic process | 1 | 0 | 0.002 | 9 | 239 |
| GO:0034641 | cellular nitrogen compound metabolic process | 1 | 0 | 0.002 | 58 | 3479 |
| GO:0043489 | RNA stabilization | 1 | 0 | 0.001 | 3 | 18 |
| GO:0048255 | mRNA stabilization | 1 | 0 | 0.001 | 3 | 18 |
| GO:0044237 | cellular metabolic process | 1 | 0 | 0.001 | 86 | 5718 |
| GO:0006401 | RNA catabolic process | 1 | 0 | 9e-04 | 5 | 62 |
| GO:0006399 | tRNA metabolic process | 1 | 0 | 7e-04 | 6 | 88 |
| GO:0000097 | sulfur amino acid biosynthetic process | 1 | 0 | 6e-04 | 3 | 14 |
| GO:0006400 | tRNA modification | 1 | 0 | 6e-04 | 3 | 14 |
| GO:0043170 | macromolecule metabolic process | 1 | 0 | 5e-04 | 76 | 4772 |
| GO:0008033 | tRNA processing | 1 | 0 | 5e-04 | 5 | 56 |
| GO:0071265 | L-methionine biosynthetic process | 1 | 0 | 4e-04 | 2 | 3 |
| GO:0071267 | L-methionine salvage | 1 | 0 | 4e-04 | 2 | 3 |
| GO:0043487 | regulation of RNA stability | 1 | 0 | 2e-04 | 4 | 25 |
| GO:0009057 | macromolecule catabolic process | 1 | 0 | 2e-04 | 16 | 478 |
| GO:0043488 | regulation of mRNA stability | 1 | 0 | 2e-04 | 4 | 24 |
| GO:0016070 | RNA metabolic process | 1 | 0 | 1e-04 | 41 | 1940 |
| GO:0090304 | nucleic acid metabolic process | 1 | 0 | 9e-05 | 53 | 2768 |
| GO:0044265 | cellular macromolecule catabolic process | 1 | 0 | 9e-05 | 15 | 401 |
| GO:0010467 | gene expression | 1 | 0 | 9e-05 | 54 | 2838 |
| GO:0042254 | ribosome biogenesis | 1 | 0 | 7e-05 | 8 | 114 |
| GO:0022613 | ribonucleoprotein complex biogenesis | 1 | 0 | 4e-05 | 10 | 170 |
| GO:0044260 | cellular macromolecule metabolic process | 1 | 0 | 2e-05 | 75 | 4325 |
| GO:0034660 | ncRNA metabolic process | 1 | 0 | 1e-05 | 11 | 186 |
| GO:0006397 | mRNA processing | 1 | 0 | 1e-05 | 13 | 257 |
| GO:0034470 | ncRNA processing | 1 | 0 | 3e-06 | 11 | 157 |
| GO:0008380 | RNA splicing | 1 | 0 | 3e-07 | 15 | 251 |
| GO:0016071 | mRNA metabolic process | 1 | 0 | 5e-09 | 19 | 310 |
| GO:0006396 | RNA processing | 1 | 0 | 5e-11 | 26 | 475 |

### Cellular Component

- Entrez Gene IDs have annotations in category 'CC'
- of these are in the above list
- upreg means upregulated in group IL21\_regulated.1 and downreg means downregulated in group IL21\_regulated.1

|  |  |  |  |  |  |  |
| --- | --- | --- | --- | --- | --- | --- |
| **GO ID** | **GO Term** | **upreg. p-value** | **upreg. int. Count** | **downreg. p-value** | **downreg. int. Count** | **GO Count** |
| GO:0043228 | non-membrane-bounded organelle | 1 | 0 | 0.009 | 36 | 2087 |
| GO:0043232 | intracellular non-membrane-bounded organelle | 1 | 0 | 0.009 | 36 | 2087 |
| GO:0005732 | small nucleolar ribonucleoprotein complex | 1 | 0 | 0.007 | 2 | 11 |
| GO:0005643 | nuclear pore | 1 | 0 | 0.007 | 4 | 64 |
| GO:0005744 | mitochondrial inner membrane presequence translocase complex | 1 | 0 | 0.006 | 2 | 10 |
| GO:0032991 | macromolecular complex | 1 | 0 | 0.001 | 46 | 2584 |
| GO:0016604 | nuclear body | 1 | 0 | 0.001 | 8 | 173 |
| GO:0005654 | nucleoplasm | 1 | 0 | 6e-04 | 21 | 821 |
| GO:0043226 | organelle | 1 | 0 | 3e-04 | 101 | 6947 |
| GO:0043229 | intracellular organelle | 1 | 0 | 3e-04 | 101 | 6933 |
| GO:0044422 | organelle part | 1 | 0 | 3e-04 | 67 | 3992 |
| GO:0044446 | intracellular organelle part | 1 | 0 | 2e-04 | 67 | 3939 |
| GO:0005622 | intracellular | 1 | 0 | 1e-04 | 116 | 8358 |
| GO:0044424 | intracellular part | 1 | 0 | 6e-05 | 115 | 8157 |
| GO:0043227 | membrane-bounded organelle | 1 | 0 | 5e-05 | 96 | 6268 |
| GO:0043231 | intracellular membrane-bounded organelle | 1 | 0 | 5e-05 | 96 | 6261 |
| GO:0016607 | nuclear speck | 1 | 0 | 2e-05 | 8 | 99 |
| GO:0005681 | spliceosomal complex | 1 | 0 | 7e-06 | 9 | 113 |
| GO:0043233 | organelle lumen | 1 | 0 | 1e-06 | 40 | 1591 |
| GO:0030529 | ribonucleoprotein complex | 1 | 0 | 1e-06 | 18 | 409 |
| GO:0031974 | membrane-enclosed lumen | 1 | 0 | 9e-07 | 41 | 1622 |
| GO:0070013 | intracellular organelle lumen | 1 | 0 | 8e-07 | 40 | 1555 |
| GO:0005730 | nucleolus | 1 | 0 | 5e-07 | 23 | 604 |
| GO:0005634 | nucleus | 1 | 0 | 1e-07 | 75 | 3864 |
| GO:0031981 | nuclear lumen | 1 | 0 | 4e-08 | 38 | 1280 |
| GO:0044428 | nuclear part | 1 | 0 | 1e-09 | 47 | 1625 |

### Distribution of KEGG annotations

- Up regulated probes with KEGG annotations in above list: 0
- Down regulated probes with KEGG annotations in above list: 69
- The chip holds 7585 probes annotated to 214 pathways

|  |  |  |  |  |  |  |
| --- | --- | --- | --- | --- | --- | --- |
| **KEGG ID** | **Path Name** | **upreg.p.value** | **upreg.Int.Count** | **downreg.p.value** | **downreg.Int.Count** | **KEGG.Count** |
| 03018 | RNA degradation | 1 | 0 | 0.006 | 4 | 82 |
| 03022 | Basal transcription factors | 1 | 0 | 0.006 | 3 | 42 |
| 03040 | Spliceosome | 1 | 0 | <2e-16 | 25 | 203 |
| 03420 | Nucleotide excision repair | 1 | 0 | 0.002 | 4 | 58 |
| 04120 | Ubiquitin mediated proteolysis | 1 | 0 | 3e-04 | 9 | 235 |
| 04612 | Antigen processing and presentation | 1 | 0 | 3e-04 | 7 | 141 |

#99CCCC #CCCCCC #E8E8E8

Annotations from:

- Data package 'hgu133a.db' version 2.4.5 packaged on 2010-09-23 21:50:14 UTC; mcarlson
- Data package 'GO.db' version 2.4.5 packaged on 2010-09-23 21:49:10 UTC; mcarlson
- Data package 'KEGG.db' version 2.4.5 packaged on 2010-09-23 22:03:46 UTC; mcarlson
