# Supplementary material for: Global gene expression changes of in vitro stimulated human transformed germinal centre B cells as surrogate for oncogenic pathway activation in individual aggressive B cell lymphomas
Source: Cell Commun Signal. 2012 Dec 20;10:43. doi: 10.1186/1478-811X-10-43 (PMC3566944; doi:10.1186/1478-811X-10-43)
Supplement: Additional file 9 — Supplemental 2. Geneset enrichment Analysis identifying enriched pathways in differentially expressed genes. [file 1478-811X-10-43-S9.zip › supplementalFile2_GO_AnalysenLIMMA/IL21.2_dn.html]

- 13 unique Entrez Gene IDs considered
- on chip with 22283 probesets

- Molecular function
- Biological process
- Cellular component
- Pathways (KEGG)

### Molecular Function

- 10870 Entrez Gene IDs have annotations in category 'MF'
- of these are in the above list
- upreg means upregulated in group IL21\_regulated.2 and downreg means downregulated in group IL21\_regulated.2

|  |  |  |  |  |  |  |
| --- | --- | --- | --- | --- | --- | --- |
| **GO ID** | **GO Term** | **upreg. p-value** | **upreg. int. Count** | **downreg. p-value** | **downreg. int. Count** | **GO Count** |
| GO:0005057 | receptor signaling protein activity | 0.008 | 2 | 1 | 0 | 149 |

### Biological Process

- 10392 Entrez Gene IDs have annotations in category 'BP'
- of these are in the above list
- upreg means upregulated in group IL21\_regulated.2 and downreg means downregulated in group IL21\_regulated.2

|  |  |  |  |  |  |  |
| --- | --- | --- | --- | --- | --- | --- |
| **GO ID** | **GO Term** | **upreg. p-value** | **upreg. int. Count** | **downreg. p-value** | **downreg. int. Count** | **GO Count** |
| GO:0009615 | response to virus | 9e-09 | 6 | 1 | 0 | 141 |
| GO:0051707 | response to other organism | 3e-08 | 7 | 1 | 0 | 315 |
| GO:0009607 | response to biotic stimulus | 1e-07 | 7 | 1 | 0 | 384 |
| GO:0051704 | multi-organism process | 1e-05 | 7 | 1 | 0 | 729 |
| GO:0006955 | immune response | 5e-05 | 6 | 1 | 0 | 619 |
| GO:0002376 | immune system process | 5e-05 | 7 | 1 | 0 | 948 |
| GO:0006952 | defense response | 5e-04 | 5 | 1 | 0 | 598 |
| GO:0050896 | response to stimulus | 0.002 | 9 | 1 | 0 | 2834 |
| GO:0009612 | response to mechanical stimulus | 0.002 | 2 | 1 | 0 | 58 |
| GO:0032496 | response to lipopolysaccharide | 0.009 | 2 | 1 | 0 | 117 |
| GO:0006950 | response to stress | 0.009 | 6 | 1 | 0 | 1620 |

### Cellular Component

- no worthwhile CC annotations found

### Distribution of KEGG annotations

- Up regulated probes with KEGG annotations in above list: 9
- Down regulated probes with KEGG annotations in above list: 0
- The chip holds 7585 probes annotated to 214 pathways

|  |  |  |  |  |  |  |
| --- | --- | --- | --- | --- | --- | --- |
| **KEGG ID** | **Path Name** | **upreg.p.value** | **upreg.Int.Count** | **downreg.p.value** | **downreg.Int.Count** | **KEGG.Count** |
| 04620 | Toll-like receptor signaling pathway | 5e-11 | 7 | 1 | 0 | 155 |
| 05140 | Leishmaniasis | 2e-09 | 6 | 1 | 0 | 126 |
| 04062 | Chemokine signaling pathway | 3e-09 | 7 | 1 | 0 | 276 |
| 05212 | Pancreatic cancer | 3e-09 | 6 | 1 | 0 | 143 |
| 04630 | Jak-STAT signaling pathway | 8e-08 | 6 | 1 | 0 | 245 |
| 05200 | Pathways in cancer | 2e-05 | 6 | 1 | 0 | 631 |
| 04662 | B cell receptor signaling pathway | 0.01 | 2 | 1 | 0 | 130 |

#99CCCC #CCCCCC #E8E8E8

Annotations from:

- Data package 'hgu133a.db' version 2.4.5 packaged on 2010-09-23 21:50:14 UTC; mcarlson
- Data package 'GO.db' version 2.4.5 packaged on 2010-09-23 21:49:10 UTC; mcarlson
- Data package 'KEGG.db' version 2.4.5 packaged on 2010-09-23 22:03:46 UTC; mcarlson
