# Supplementary material for: Global gene expression changes of in vitro stimulated human transformed germinal centre B cells as surrogate for oncogenic pathway activation in individual aggressive B cell lymphomas
Source: Cell Commun Signal. 2012 Dec 20;10:43. doi: 10.1186/1478-811X-10-43 (PMC3566944; doi:10.1186/1478-811X-10-43)
Supplement: Additional file 9 — Supplemental 2. Geneset enrichment Analysis identifying enriched pathways in differentially expressed genes. [file 1478-811X-10-43-S9.zip › supplementalFile2_GO_AnalysenLIMMA/LPS.1_up.html]

- 37 unique Entrez Gene IDs considered
- on chip with 22283 probesets

- Molecular function
- Biological process
- Cellular component
- Pathways (KEGG)

### Molecular Function

- 10870 Entrez Gene IDs have annotations in category 'MF'
- 34 of these are in the above list
- upreg means upregulated in group LPS\_regulated.1 and downreg means downregulated in group LPS\_regulated.1

|  |  |  |  |  |  |  |
| --- | --- | --- | --- | --- | --- | --- |
| **GO ID** | **GO Term** | **upreg. p-value** | **upreg. int. Count** | **downreg. p-value** | **downreg. int. Count** | **GO Count** |
| GO:0003700 | sequence-specific DNA binding transcription factor activity | 1.00 | 0 | 0.001 | 8 | 749 |
| GO:0043565 | sequence-specific DNA binding | 1.00 | 0 | 6e-04 | 7 | 500 |

### Biological Process

- Entrez Gene IDs have annotations in category 'BP'
- of these are in the above list
- upreg means upregulated in group LPS\_regulated.1 and downreg means downregulated in group LPS\_regulated.1

|  |  |  |  |  |  |  |
| --- | --- | --- | --- | --- | --- | --- |
| **GO ID** | **GO Term** | **upreg. p-value** | **upreg. int. Count** | **downreg. p-value** | **downreg. int. Count** | **GO Count** |
| GO:0008286 | insulin receptor signaling pathway | 1 | 0 | 0.009 | 2 | 51 |
| GO:0030097 | hemopoiesis | 1 | 0 | 0.009 | 4 | 289 |
| GO:0032501 | multicellular organismal process | 1 | 0 | 0.009 | 17 | 3528 |
| GO:0007093 | mitotic cell cycle checkpoint | 1 | 0 | 0.008 | 2 | 48 |
| GO:0002088 | lens development in camera-type eye | 1 | 0 | 0.004 | 2 | 31 |
| GO:0000086 | G2/M transition of mitotic cell cycle | 1 | 0 | 0.003 | 2 | 29 |
| GO:0007423 | sensory organ development | 1 | 0 | 0.003 | 4 | 210 |
| GO:0002520 | immune system development | 1 | 0 | 0.002 | 5 | 333 |
| GO:0048534 | hemopoietic or lymphoid organ development | 1 | 0 | 0.002 | 5 | 307 |
| GO:0060429 | epithelium development | 1 | 0 | 0.002 | 5 | 307 |
| GO:0030855 | epithelial cell differentiation | 1 | 0 | 9e-04 | 4 | 154 |
| GO:0048856 | anatomical structure development | 1 | 0 | 8e-04 | 15 | 2332 |
| GO:0030099 | myeloid cell differentiation | 1 | 0 | 8e-04 | 4 | 145 |
| GO:0001654 | eye development | 1 | 0 | 6e-04 | 4 | 138 |
| GO:0007275 | multicellular organismal development | 1 | 0 | 6e-04 | 16 | 2529 |
| GO:0048821 | erythrocyte development | 1 | 0 | 5e-04 | 2 | 12 |
| GO:0032502 | developmental process | 1 | 0 | 5e-04 | 17 | 2770 |
| GO:0048731 | system development | 1 | 0 | 3e-04 | 15 | 2157 |
| GO:0048513 | organ development | 1 | 0 | 3e-04 | 13 | 1651 |
| GO:0043010 | camera-type eye development | 1 | 0 | 3e-04 | 4 | 110 |
| GO:0043249 | erythrocyte maturation | 1 | 0 | 2e-04 | 2 | 8 |
| GO:0048869 | cellular developmental process | 1 | 0 | 3e-05 | 14 | 1541 |
| GO:0030154 | cell differentiation | 1 | 0 | 2e-05 | 14 | 1504 |

### Cellular Component

- no worthwhile CC annotations found

### Distribution of KEGG annotations

- no worthwhile KEGG annotations found

Annotations from:

- Data package 'hgu133a.db' version 2.4.5 packaged on 2010-09-23 21:50:14 UTC; mcarlson
- Data package 'GO.db' version 2.4.5 packaged on 2010-09-23 21:49:10 UTC; mcarlson
- Data package 'KEGG.db' version 2.4.5 packaged on 2010-09-23 22:03:46 UTC; mcarlson
