# Supplementary material for: Global gene expression changes of in vitro stimulated human transformed germinal centre B cells as surrogate for oncogenic pathway activation in individual aggressive B cell lymphomas
Source: Cell Commun Signal. 2012 Dec 20;10:43. doi: 10.1186/1478-811X-10-43 (PMC3566944; doi:10.1186/1478-811X-10-43)
Supplement: Additional file 9 — Supplemental 2. Geneset enrichment Analysis identifying enriched pathways in differentially expressed genes. [file 1478-811X-10-43-S9.zip › supplementalFile2_GO_AnalysenLIMMA/LPS.2_dn.html]

- 36 unique Entrez Gene IDs considered
- on chip with 22283 probesets

- Molecular function
- Biological process
- Cellular component
- Pathways (KEGG)

### Molecular Function

- 10870 Entrez Gene IDs have annotations in category 'MF'
- 32 of these are in the above list
- upreg means upregulated in group LPS\_regulated.2 and downreg means downregulated in group LPS\_regulated.2

|  |  |  |  |  |  |  |
| --- | --- | --- | --- | --- | --- | --- |
| **GO ID** | **GO Term** | **upreg. p-value** | **upreg. int. Count** | **downreg. p-value** | **downreg. int. Count** | **GO Count** |
| GO:0015276 | ligand-gated ion channel activity | 0.003 | 3 | 1.000 | 0 | 106 |
| GO:0022834 | ligand-gated channel activity | 0.003 | 3 | 1.000 | 0 | 106 |
| GO:0016247 | channel regulator activity | 0.007 | 2 | 1.000 | 0 | 49 |
| GO:0008134 | transcription factor binding | 1.000 | 0 | 0.005 | 2 | 469 |

### Biological Process

- 10392 Entrez Gene IDs have annotations in category 'BP'
- 30 of these are in the above list
- upreg means upregulated in group LPS\_regulated.2 and downreg means downregulated in group LPS\_regulated.2

|  |  |  |  |  |  |  |
| --- | --- | --- | --- | --- | --- | --- |
| **GO ID** | **GO Term** | **upreg. p-value** | **upreg. int. Count** | **downreg. p-value** | **downreg. int. Count** | **GO Count** |
| GO:0014048 | regulation of glutamate secretion | 6e-06 | 3 | 1.000 | 0 | 14 |
| GO:0060079 | regulation of excitatory postsynaptic membrane potential | 9e-06 | 3 | 1.000 | 0 | 16 |
| GO:0014047 | glutamate secretion | 1e-05 | 3 | 1.000 | 0 | 19 |
| GO:0060078 | regulation of postsynaptic membrane potential | 2e-05 | 3 | 1.000 | 0 | 20 |
| GO:0050877 | neurological system process | 7e-05 | 9 | 1.000 | 0 | 752 |
| GO:0051899 | membrane depolarization | 1e-04 | 3 | 1.000 | 0 | 39 |
| GO:0007268 | synaptic transmission | 1e-04 | 6 | 1.000 | 0 | 323 |
| GO:0032026 | response to magnesium ion | 2e-04 | 2 | 1.000 | 0 | 9 |
| GO:0003008 | system process | 2e-04 | 10 | 1.000 | 0 | 1089 |
| GO:0048167 | regulation of synaptic plasticity | 3e-04 | 3 | 1.000 | 0 | 48 |
| GO:0019226 | transmission of nerve impulse | 3e-04 | 6 | 1.000 | 0 | 375 |
| GO:0003001 | generation of a signal involved in cell-cell signaling | 7e-04 | 4 | 1.000 | 0 | 160 |
| GO:0023061 | signal release | 7e-04 | 4 | 1.000 | 0 | 160 |
| GO:0048169 | regulation of long-term neuronal synaptic plasticity | 8e-04 | 2 | 1.000 | 0 | 16 |
| GO:0048008 | platelet-derived growth factor receptor signaling pathway | 0.001 | 2 | 1.000 | 0 | 20 |
| GO:0032501 | multicellular organismal process | 0.002 | 17 | 0.712 | 1 | 3528 |
| GO:0007423 | sensory organ development | 0.002 | 4 | 1.000 | 0 | 210 |
| GO:0048168 | regulation of neuronal synaptic plasticity | 0.003 | 2 | 1.000 | 0 | 29 |
| GO:0030001 | metal ion transport | 0.003 | 5 | 1.000 | 0 | 391 |
| GO:0050804 | regulation of synaptic transmission | 0.003 | 3 | 1.000 | 0 | 114 |
| GO:0050801 | ion homeostasis | 0.003 | 5 | 1.000 | 0 | 395 |
| GO:0065008 | regulation of biological quality | 0.003 | 10 | 0.376 | 1 | 1511 |
| GO:0045833 | negative regulation of lipid metabolic process | 0.003 | 2 | 1.000 | 0 | 33 |
| GO:0019216 | regulation of lipid metabolic process | 0.003 | 3 | 1.000 | 0 | 117 |
| GO:0051047 | positive regulation of secretion | 0.004 | 3 | 1.000 | 0 | 121 |
| GO:0007193 | inhibition of adenylate cyclase activity by G-protein signaling pathway | 0.004 | 2 | 1.000 | 0 | 36 |
| GO:0051969 | regulation of transmission of nerve impulse | 0.004 | 3 | 1.000 | 0 | 125 |
| GO:0019725 | cellular homeostasis | 0.005 | 5 | 1.000 | 0 | 432 |
| GO:0042391 | regulation of membrane potential | 0.005 | 3 | 1.000 | 0 | 134 |
| GO:0031644 | regulation of neurological system process | 0.005 | 3 | 1.000 | 0 | 135 |
| GO:0007267 | cell-cell signaling | 0.005 | 6 | 1.000 | 0 | 638 |
| GO:0006811 | ion transport | 0.005 | 6 | 1.000 | 0 | 641 |
| GO:0043270 | positive regulation of ion transport | 0.006 | 2 | 1.000 | 0 | 44 |
| GO:0050905 | neuromuscular process | 0.006 | 2 | 1.000 | 0 | 44 |
| GO:0007269 | neurotransmitter secretion | 0.006 | 2 | 1.000 | 0 | 45 |
| GO:0006812 | cation transport | 0.006 | 5 | 1.000 | 0 | 464 |
| GO:0007194 | negative regulation of adenylate cyclase activity | 0.007 | 2 | 1.000 | 0 | 50 |
| GO:0031280 | negative regulation of cyclase activity | 0.007 | 2 | 1.000 | 0 | 50 |
| GO:0051350 | negative regulation of lyase activity | 0.007 | 2 | 1.000 | 0 | 50 |
| GO:0009582 | detection of abiotic stimulus | 0.008 | 2 | 1.000 | 0 | 51 |
| GO:0048878 | chemical homeostasis | 0.009 | 5 | 1.000 | 0 | 503 |
| GO:0051129 | negative regulation of cellular component organization | 0.009 | 3 | 1.000 | 0 | 165 |
| GO:0007270 | nerve-nerve synaptic transmission | 0.010 | 2 | 1.000 | 0 | 57 |
| GO:0032940 | secretion by cell | 0.010 | 4 | 1.000 | 0 | 328 |
| GO:0051246 | regulation of protein metabolic process | 0.180 | 3 | 0.009 | 2 | 566 |
| GO:0009057 | macromolecule catabolic process | 1.000 | 0 | 0.006 | 2 | 478 |
| GO:0044265 | cellular macromolecule catabolic process | 1.000 | 0 | 0.004 | 2 | 401 |
| GO:0030163 | protein catabolic process | 1.000 | 0 | 0.003 | 2 | 329 |
| GO:0044257 | cellular protein catabolic process | 1.000 | 0 | 0.002 | 2 | 287 |
| GO:0051603 | proteolysis involved in cellular protein catabolic process | 1.000 | 0 | 0.002 | 2 | 285 |
| GO:0009894 | regulation of catabolic process | 0.122 | 2 | 0.001 | 2 | 233 |
| GO:0042176 | regulation of protein catabolic process | 1.000 | 0 | 2e-04 | 2 | 77 |

### Cellular Component

- 11181 Entrez Gene IDs have annotations in category 'CC'
- 32 of these are in the above list
- upreg means upregulated in group LPS\_regulated.2 and downreg means downregulated in group LPS\_regulated.2

|  |  |  |  |  |  |  |
| --- | --- | --- | --- | --- | --- | --- |
| **GO ID** | **GO Term** | **upreg. p-value** | **upreg. int. Count** | **downreg. p-value** | **downreg. int. Count** | **GO Count** |
| GO:0031012 | extracellular matrix | 9e-04 | 5 | 1.000 | 0 | 296 |
| GO:0044456 | synapse part | 0.002 | 4 | 1.000 | 0 | 213 |
| GO:0042734 | presynaptic membrane | 0.003 | 2 | 1.000 | 0 | 32 |
| GO:0045211 | postsynaptic membrane | 0.004 | 3 | 1.000 | 0 | 123 |
| GO:0031226 | intrinsic to plasma membrane | 0.005 | 8 | 1.000 | 0 | 1094 |
| GO:0044463 | cell projection part | 0.006 | 4 | 1.000 | 0 | 283 |
| GO:0045202 | synapse | 0.007 | 4 | 1.000 | 0 | 295 |
| GO:0005576 | extracellular region | 0.007 | 9 | 1.000 | 0 | 1391 |
| GO:0031091 | platelet alpha granule | 0.008 | 2 | 1.000 | 0 | 51 |
| GO:0009986 | cell surface | 0.008 | 4 | 0.081 | 1 | 310 |
| GO:0043005 | neuron projection | 0.008 | 4 | 1.000 | 0 | 315 |
| GO:0044459 | plasma membrane part | 0.009 | 10 | 0.395 | 1 | 1724 |

### Distribution of KEGG annotations

- Up regulated probes with KEGG annotations in above list: 27
- Down regulated probes with KEGG annotations in above list: 0
- The chip holds 7585 probes annotated to 214 pathways

|  |  |  |  |  |  |  |
| --- | --- | --- | --- | --- | --- | --- |
| **KEGG ID** | **Path Name** | **upreg.p.value** | **upreg.Int.Count** | **downreg.p.value** | **downreg.Int.Count** | **KEGG.Count** |
| 00670 | One carbon pool by folate | 1e-04 | 3 | 1 | 0 | 27 |
| 05010 | Alzheimer's disease | 2e-04 | 6 | 1 | 0 | 258 |
| 04080 | Neuroactive ligand-receptor interaction | 3e-04 | 7 | 1 | 0 | 388 |
| 04950 | Maturity onset diabetes of the young | 3e-04 | 3 | 1 | 0 | 39 |
| 00601 | Glycosphingolipid biosynthesis - lacto and neolacto series | 5e-04 | 3 | 1 | 0 | 44 |
| 00620 | Pyruvate metabolism | 0.001 | 3 | 1 | 0 | 57 |
| 05214 | Glioma | 0.001 | 4 | 1 | 0 | 135 |
| 05218 | Melanoma | 0.001 | 4 | 1 | 0 | 137 |
| 05211 | Renal cell carcinoma | 0.001 | 4 | 1 | 0 | 142 |
| 05012 | Parkinson's disease | 0.002 | 4 | 1 | 0 | 158 |
| 04930 | Type II diabetes mellitus | 0.003 | 3 | 1 | 0 | 78 |
| 04540 | Gap junction | 0.004 | 4 | 1 | 0 | 184 |
| 00010 | Glycolysis / Gluconeogenesis | 0.004 | 3 | 1 | 0 | 95 |
| 05215 | Prostate cancer | 0.005 | 4 | 1 | 0 | 193 |

#99CCCC #CCCCCC #E8E8E8

Annotations from:

- Data package 'hgu133a.db' version 2.4.5 packaged on 2010-09-23 21:50:14 UTC; mcarlson
- Data package 'GO.db' version 2.4.5 packaged on 2010-09-23 21:49:10 UTC; mcarlson
- Data package 'KEGG.db' version 2.4.5 packaged on 2010-09-23 22:03:46 UTC; mcarlson
