# Supplementary material for: Global gene expression changes of in vitro stimulated human transformed germinal centre B cells as surrogate for oncogenic pathway activation in individual aggressive B cell lymphomas
Source: Cell Commun Signal. 2012 Dec 20;10:43. doi: 10.1186/1478-811X-10-43 (PMC3566944; doi:10.1186/1478-811X-10-43)
Supplement: Additional file 20 — Supplemental 3. Geneset enrichment Analysis identifying enriched pathways in differentially expressed genes overlapping between stimulations. [file 1478-811X-10-43-S20.zip › supplementalFIle3_GO_AnalysenOverlaps/BCR_CD40_DOWN.html]

- 35 unique Entrez Gene IDs considered
- on chip with 54675 probesets

- Molecular function
- Biological process
- Cellular component
- Pathways (KEGG)

### Molecular Function

- no worthwhile MF annotations found

### Biological Process

- 12592 Entrez Gene IDs have annotations in category 'BP'
- 21 of these are in the above list

|  |  |  |  |  |
| --- | --- | --- | --- | --- |
| **GO ID** | **GO Term** | **p-value** | **int. Count** | **GO Count** |
| GO:0045669 | positive regulation of osteoblast differentiation | 4e-04 | 2 | 19 |
| GO:0006793 | phosphorus metabolic process | 5e-04 | 7 | 908 |
| GO:0006796 | phosphate metabolic process | 5e-04 | 7 | 908 |
| GO:0050871 | positive regulation of B cell activation | 5e-04 | 2 | 20 |
| GO:0001707 | mesoderm formation | 8e-04 | 2 | 26 |
| GO:0045667 | regulation of osteoblast differentiation | 9e-04 | 2 | 27 |
| GO:0048332 | mesoderm morphogenesis | 1e-03 | 2 | 28 |
| GO:0001704 | formation of primary germ layer | 0.001 | 2 | 29 |
| GO:0050864 | regulation of B cell activation | 0.001 | 2 | 34 |
| GO:0016311 | dephosphorylation | 0.001 | 3 | 137 |
| GO:0045597 | positive regulation of cell differentiation | 0.002 | 3 | 139 |
| GO:0050671 | positive regulation of lymphocyte proliferation | 0.002 | 2 | 35 |
| GO:0032946 | positive regulation of mononuclear cell proliferation | 0.002 | 2 | 36 |
| GO:0042127 | regulation of cell proliferation | 0.002 | 5 | 567 |
| GO:0001649 | osteoblast differentiation | 0.003 | 2 | 45 |
| GO:0030509 | BMP signaling pathway | 0.003 | 2 | 49 |
| GO:0030278 | regulation of ossification | 0.003 | 2 | 50 |
| GO:0050670 | regulation of lymphocyte proliferation | 0.003 | 2 | 50 |
| GO:0032944 | regulation of mononuclear cell proliferation | 0.003 | 2 | 51 |
| GO:0007369 | gastrulation | 0.003 | 2 | 53 |
| GO:0007498 | mesoderm development | 0.004 | 2 | 54 |
| GO:0046850 | regulation of bone remodeling | 0.004 | 2 | 56 |
| GO:0007154 | cell communication | 0.004 | 12 | 3456 |
| GO:0001934 | positive regulation of protein amino acid phosphorylation | 0.004 | 2 | 58 |
| GO:0034103 | regulation of tissue remodeling | 0.004 | 2 | 59 |
| GO:0046849 | bone remodeling | 0.005 | 2 | 63 |
| GO:0051251 | positive regulation of lymphocyte activation | 0.005 | 2 | 63 |
| GO:0051094 | positive regulation of developmental process | 0.005 | 4 | 434 |
| GO:0042327 | positive regulation of phosphorylation | 0.005 | 2 | 66 |
| GO:0010562 | positive regulation of phosphorus metabolic process | 0.006 | 2 | 68 |
| GO:0045937 | positive regulation of phosphate metabolic process | 0.006 | 2 | 68 |
| GO:0046651 | lymphocyte proliferation | 0.006 | 2 | 68 |
| GO:0032943 | mononuclear cell proliferation | 0.006 | 2 | 70 |
| GO:0002696 | positive regulation of leukocyte activation | 0.006 | 2 | 71 |
| GO:0007165 | signal transduction | 0.006 | 11 | 3134 |
| GO:0031401 | positive regulation of protein modification process | 0.006 | 2 | 73 |
| GO:0050867 | positive regulation of cell activation | 0.006 | 2 | 73 |
| GO:0042113 | B cell activation | 0.009 | 2 | 84 |
| GO:0048771 | tissue remodeling | 0.009 | 2 | 85 |
| GO:0001932 | regulation of protein amino acid phosphorylation | 0.009 | 2 | 87 |
| GO:0048729 | tissue morphogenesis | 0.010 | 2 | 90 |
| GO:0008285 | negative regulation of cell proliferation | 0.010 | 3 | 271 |

### Cellular Component

- no worthwhile CC annotations found

### Distribution of KEGG annotations

- no worthwhile KEGG annotations found

Annotations from:

- Data package 'hgu133plus2.db' version 2.2.11 packaged on Wed Mar 25 18:42:48 2009; mcarlson
- Data package 'GO.db' version 2.2.11 packaged on Wed Mar 25 18:36:02 2009; mcarlson
- Data package 'KEGG.db' version 2.2.11 packaged on Wed Mar 25 19:13:17 2009; mcarlson
