# Supplementary material for: Global gene expression changes of in vitro stimulated human transformed germinal centre B cells as surrogate for oncogenic pathway activation in individual aggressive B cell lymphomas
Source: Cell Commun Signal. 2012 Dec 20;10:43. doi: 10.1186/1478-811X-10-43 (PMC3566944; doi:10.1186/1478-811X-10-43)
Supplement: Additional file 20 — Supplemental 3. Geneset enrichment Analysis identifying enriched pathways in differentially expressed genes overlapping between stimulations. [file 1478-811X-10-43-S20.zip › supplementalFIle3_GO_AnalysenOverlaps/BCR_CD40_LPS_UP.html]

- 17 unique Entrez Gene IDs considered
- on chip with 54675 probesets

- Molecular function
- Biological process
- Cellular component
- Pathways (KEGG)

### Molecular Function

- 13686 Entrez Gene IDs have annotations in category 'MF'
- 14 of these are in the above list

|  |  |  |  |  |
| --- | --- | --- | --- | --- |
| **GO ID** | **GO Term** | **p-value** | **int. Count** | **GO Count** |
| GO:0032403 | protein complex binding | 0.007 | 2 | 122 |

### Biological Process

- 12592 Entrez Gene IDs have annotations in category 'BP'
- 14 of these are in the above list

|  |  |  |  |  |
| --- | --- | --- | --- | --- |
| **GO ID** | **GO Term** | **p-value** | **int. Count** | **GO Count** |
| GO:0050794 | regulation of cellular process | 0.003 | 12 | 5884 |
| GO:0050789 | regulation of biological process | 0.005 | 12 | 6110 |
| GO:0008632 | apoptotic program | 0.005 | 2 | 101 |
| GO:0065007 | biological regulation | 0.008 | 12 | 6466 |

### Cellular Component

- 14379 Entrez Gene IDs have annotations in category 'CC'
- 15 of these are in the above list

|  |  |  |  |  |
| --- | --- | --- | --- | --- |
| **GO ID** | **GO Term** | **p-value** | **int. Count** | **GO Count** |
| GO:0005637 | nuclear inner membrane | 2e-04 | 2 | 21 |
| GO:0005635 | nuclear envelope | 0.001 | 3 | 199 |
| GO:0031967 | organelle envelope | 0.002 | 4 | 552 |
| GO:0031975 | envelope | 0.002 | 4 | 553 |
| GO:0031965 | nuclear membrane | 0.006 | 2 | 110 |

### Distribution of KEGG annotations

- no worthwhile KEGG annotations found

Annotations from:

- Data package 'hgu133plus2.db' version 2.2.11 packaged on Wed Mar 25 18:42:48 2009; mcarlson
- Data package 'GO.db' version 2.2.11 packaged on Wed Mar 25 18:36:02 2009; mcarlson
- Data package 'KEGG.db' version 2.2.11 packaged on Wed Mar 25 19:13:17 2009; mcarlson
