# Supplementary material for: Global gene expression changes of in vitro stimulated human transformed germinal centre B cells as surrogate for oncogenic pathway activation in individual aggressive B cell lymphomas
Source: Cell Commun Signal. 2012 Dec 20;10:43. doi: 10.1186/1478-811X-10-43 (PMC3566944; doi:10.1186/1478-811X-10-43)
Supplement: Additional file 20 — Supplemental 3. Geneset enrichment Analysis identifying enriched pathways in differentially expressed genes overlapping between stimulations. [file 1478-811X-10-43-S20.zip › supplementalFIle3_GO_AnalysenOverlaps/BCR_CD40_UP.html]

- 33 unique Entrez Gene IDs considered
- on chip with 54675 probesets

- Molecular function
- Biological process
- Cellular component
- Pathways (KEGG)

### Molecular Function

- no worthwhile MF annotations found

### Biological Process

- 12592 Entrez Gene IDs have annotations in category 'BP'
- 24 of these are in the above list

|  |  |  |  |  |
| --- | --- | --- | --- | --- |
| **GO ID** | **GO Term** | **p-value** | **int. Count** | **GO Count** |
| GO:0006955 | immune response | 1e-05 | 8 | 625 |
| GO:0002376 | immune system process | 1e-04 | 8 | 878 |
| GO:0050896 | response to stimulus | 0.003 | 11 | 2449 |
| GO:0019882 | antigen processing and presentation | 0.005 | 2 | 55 |

### Cellular Component

- 14379 Entrez Gene IDs have annotations in category 'CC'
- 28 of these are in the above list

|  |  |  |  |  |
| --- | --- | --- | --- | --- |
| **GO ID** | **GO Term** | **p-value** | **int. Count** | **GO Count** |
| GO:0042611 | MHC protein complex | 0.002 | 2 | 33 |

### Distribution of KEGG annotations

- Probes with KEGG annotations in above list: 16
- The chip holds 9722 probes annotated to 205 pathways

|  |  |  |  |  |
| --- | --- | --- | --- | --- |
| **KEGG ID** | **Path Name** | **p.value** | **Int.Count** | **KEGG.Count** |
| 04514 | Cell adhesion molecules (CAMs) | 0.003 | 6 | 345 |
| 05330 | Allograft rejection | 0.006 | 3 | 85 |
| 05332 | Graft-versus-host disease | 0.007 | 3 | 93 |

Annotations from:

- Data package 'hgu133plus2.db' version 2.2.11 packaged on Wed Mar 25 18:42:48 2009; mcarlson
- Data package 'GO.db' version 2.2.11 packaged on Wed Mar 25 18:36:02 2009; mcarlson
- Data package 'KEGG.db' version 2.2.11 packaged on Wed Mar 25 19:13:17 2009; mcarlson
