# Supplementary material for: Global gene expression changes of in vitro stimulated human transformed germinal centre B cells as surrogate for oncogenic pathway activation in individual aggressive B cell lymphomas
Source: Cell Commun Signal. 2012 Dec 20;10:43. doi: 10.1186/1478-811X-10-43 (PMC3566944; doi:10.1186/1478-811X-10-43)
Supplement: Additional file 20 — Supplemental 3. Geneset enrichment Analysis identifying enriched pathways in differentially expressed genes overlapping between stimulations. [file 1478-811X-10-43-S20.zip › supplementalFIle3_GO_AnalysenOverlaps/BCR_LPS_DOWN.html]

- 7 unique Entrez Gene IDs considered
- on chip with 54675 probesets

- Molecular function
- Biological process
- Cellular component
- Pathways (KEGG)

### Molecular Function

- 13686 Entrez Gene IDs have annotations in category 'MF'
- 4 of these are in the above list

|  |  |  |  |  |
| --- | --- | --- | --- | --- |
| **GO ID** | **GO Term** | **p-value** | **int. Count** | **GO Count** |
| GO:0030695 | GTPase regulator activity | 0.004 | 2 | 370 |

### Biological Process

- 12592 Entrez Gene IDs have annotations in category 'BP'
- 3 of these are in the above list

|  |  |  |  |  |
| --- | --- | --- | --- | --- |
| **GO ID** | **GO Term** | **p-value** | **int. Count** | **GO Count** |
| GO:0009966 | regulation of signal transduction | 1e-04 | 3 | 610 |
| GO:0010646 | regulation of cell communication | 2e-04 | 3 | 684 |
| GO:0007242 | intracellular signaling cascade | 0.001 | 3 | 1425 |
| GO:0007243 | protein kinase cascade | 0.003 | 2 | 425 |
| GO:0051094 | positive regulation of developmental process | 0.003 | 2 | 434 |

### Cellular Component

- no worthwhile CC annotations found

### Distribution of KEGG annotations

- Probes with KEGG annotations in above list: 3
- The chip holds 9722 probes annotated to 205 pathways

|  |  |  |  |  |
| --- | --- | --- | --- | --- |
| **KEGG ID** | **Path Name** | **p.value** | **Int.Count** | **KEGG.Count** |
| 04210 | Apoptosis | 5e-04 | 2 | 216 |

Annotations from:

- Data package 'hgu133plus2.db' version 2.2.11 packaged on Wed Mar 25 18:42:48 2009; mcarlson
- Data package 'GO.db' version 2.2.11 packaged on Wed Mar 25 18:36:02 2009; mcarlson
- Data package 'KEGG.db' version 2.2.11 packaged on Wed Mar 25 19:13:17 2009; mcarlson
