# Supplementary material for: Global gene expression changes of in vitro stimulated human transformed germinal centre B cells as surrogate for oncogenic pathway activation in individual aggressive B cell lymphomas
Source: Cell Commun Signal. 2012 Dec 20;10:43. doi: 10.1186/1478-811X-10-43 (PMC3566944; doi:10.1186/1478-811X-10-43)
Supplement: Additional file 20 — Supplemental 3. Geneset enrichment Analysis identifying enriched pathways in differentially expressed genes overlapping between stimulations. [file 1478-811X-10-43-S20.zip › supplementalFIle3_GO_AnalysenOverlaps/BCR_LPS_UP.html]

- 12 unique Entrez Gene IDs considered
- on chip with 54675 probesets

- Molecular function
- Biological process
- Cellular component
- Pathways (KEGG)

### Molecular Function

- 13686 Entrez Gene IDs have annotations in category 'MF'
- 11 of these are in the above list

|  |  |  |  |  |
| --- | --- | --- | --- | --- |
| **GO ID** | **GO Term** | **p-value** | **int. Count** | **GO Count** |
| GO:0016820 | hydrolase activity, acting on acid anhydrides, catalyzing transmembrane movement of substances | 0.003 | 2 | 104 |

### Biological Process

- 12592 Entrez Gene IDs have annotations in category 'BP'
- 9 of these are in the above list

|  |  |  |  |  |
| --- | --- | --- | --- | --- |
| **GO ID** | **GO Term** | **p-value** | **int. Count** | **GO Count** |
| GO:0006754 | ATP biosynthetic process | 0.002 | 2 | 85 |
| GO:0046034 | ATP metabolic process | 0.002 | 2 | 94 |
| GO:0009145 | purine nucleoside triphosphate biosynthetic process | 0.002 | 2 | 95 |
| GO:0009206 | purine ribonucleoside triphosphate biosynthetic process | 0.002 | 2 | 95 |
| GO:0009142 | nucleoside triphosphate biosynthetic process | 0.002 | 2 | 96 |
| GO:0009201 | ribonucleoside triphosphate biosynthetic process | 0.002 | 2 | 96 |
| GO:0009205 | purine ribonucleoside triphosphate metabolic process | 0.002 | 2 | 105 |
| GO:0009144 | purine nucleoside triphosphate metabolic process | 0.002 | 2 | 106 |
| GO:0009199 | ribonucleoside triphosphate metabolic process | 0.002 | 2 | 106 |
| GO:0009141 | nucleoside triphosphate metabolic process | 0.003 | 2 | 112 |
| GO:0009152 | purine ribonucleotide biosynthetic process | 0.003 | 2 | 112 |
| GO:0006164 | purine nucleotide biosynthetic process | 0.003 | 2 | 118 |
| GO:0009260 | ribonucleotide biosynthetic process | 0.003 | 2 | 120 |
| GO:0009150 | purine ribonucleotide metabolic process | 0.003 | 2 | 122 |
| GO:0009259 | ribonucleotide metabolic process | 0.004 | 2 | 132 |
| GO:0006163 | purine nucleotide metabolic process | 0.004 | 2 | 134 |

### Cellular Component

- no worthwhile CC annotations found

### Distribution of KEGG annotations

- no worthwhile KEGG annotations found

Annotations from:

- Data package 'hgu133plus2.db' version 2.2.11 packaged on Wed Mar 25 18:42:48 2009; mcarlson
- Data package 'GO.db' version 2.2.11 packaged on Wed Mar 25 18:36:02 2009; mcarlson
- Data package 'KEGG.db' version 2.2.11 packaged on Wed Mar 25 19:13:17 2009; mcarlson
