# Supplementary material for: Global gene expression changes of in vitro stimulated human transformed germinal centre B cells as surrogate for oncogenic pathway activation in individual aggressive B cell lymphomas
Source: Cell Commun Signal. 2012 Dec 20;10:43. doi: 10.1186/1478-811X-10-43 (PMC3566944; doi:10.1186/1478-811X-10-43)
Supplement: Additional file 20 — Supplemental 3. Geneset enrichment Analysis identifying enriched pathways in differentially expressed genes overlapping between stimulations. [file 1478-811X-10-43-S20.zip › supplementalFIle3_GO_AnalysenOverlaps/CD40_LPS_DOWN.html]

- 33 unique Entrez Gene IDs considered
- on chip with 54675 probesets

- Molecular function
- Biological process
- Cellular component
- Pathways (KEGG)

### Molecular Function

- 13686 Entrez Gene IDs have annotations in category 'MF'
- 25 of these are in the above list

|  |  |  |  |  |
| --- | --- | --- | --- | --- |
| **GO ID** | **GO Term** | **p-value** | **int. Count** | **GO Count** |
| GO:0005516 | calmodulin binding | 8e-05 | 4 | 128 |
| GO:0005001 | transmembrane receptor protein tyrosine phosphatase activity | 4e-04 | 2 | 17 |
| GO:0019198 | transmembrane receptor protein phosphatase activity | 4e-04 | 2 | 17 |
| GO:0003779 | actin binding | 0.002 | 4 | 288 |
| GO:0008092 | cytoskeletal protein binding | 0.007 | 4 | 421 |
| GO:0005200 | structural constituent of cytoskeleton | 0.008 | 2 | 72 |
| GO:0019901 | protein kinase binding | 0.009 | 2 | 78 |

### Biological Process

- no worthwhile BP annotations found

### Cellular Component

- no worthwhile CC annotations found

### Distribution of KEGG annotations

- Probes with KEGG annotations in above list: 13
- The chip holds 9722 probes annotated to 205 pathways

|  |  |  |  |  |
| --- | --- | --- | --- | --- |
| **KEGG ID** | **Path Name** | **p.value** | **Int.Count** | **KEGG.Count** |
| 00770 | Pantothenate and CoA biosynthesis | 0.003 | 2 | 35 |
| 00740 | Riboflavin metabolism | 0.003 | 2 | 37 |
| 00760 | Nicotinate and nicotinamide metabolism | 0.009 | 2 | 63 |
| 05340 | Primary immunodeficiency | 0.010 | 2 | 66 |
| 00500 | Starch and sucrose metabolism | 0.010 | 2 | 67 |

Annotations from:

- Data package 'hgu133plus2.db' version 2.2.11 packaged on Wed Mar 25 18:42:48 2009; mcarlson
- Data package 'GO.db' version 2.2.11 packaged on Wed Mar 25 18:36:02 2009; mcarlson
- Data package 'KEGG.db' version 2.2.11 packaged on Wed Mar 25 19:13:17 2009; mcarlson
