# Supplementary material for: Global gene expression changes of in vitro stimulated human transformed germinal centre B cells as surrogate for oncogenic pathway activation in individual aggressive B cell lymphomas
Source: Cell Commun Signal. 2012 Dec 20;10:43. doi: 10.1186/1478-811X-10-43 (PMC3566944; doi:10.1186/1478-811X-10-43)
Supplement: Additional file 20 — Supplemental 3. Geneset enrichment Analysis identifying enriched pathways in differentially expressed genes overlapping between stimulations. [file 1478-811X-10-43-S20.zip › supplementalFIle3_GO_AnalysenOverlaps/IL21_BCR_CD40_LPS_DOWN.html]

- 10 unique Entrez Gene IDs considered
- on chip with 54675 probesets

- Molecular function
- Biological process
- Cellular component
- Pathways (KEGG)

### Molecular Function

LLGOHyper 1

- 14143 Entrez Gene IDs have annotations in category 'MF'
- 6 of these are in the above list

|  |  |  |  |  |
| --- | --- | --- | --- | --- |
| **GO ID** | **GO Term** | **p-value** | **int. Count** | **GO Count** |
| GO:0042277 | peptide binding | 0.002 | 2 | 162 |

### Biological Process

LLGOHyper 1

- no worthwhile BP annotations found

### Cellular Component

LLGOHyper 1

- 14602 Entrez Gene IDs have annotations in category 'CC'
- 5 of these are in the above list

|  |  |  |  |  |
| --- | --- | --- | --- | --- |
| **GO ID** | **GO Term** | **p-value** | **int. Count** | **GO Count** |
| GO:0031965 | nuclear membrane | 0.001 | 2 | 144 |
| GO:0005635 | nuclear envelope | 0.001 | 2 | 179 |
| GO:0012505 | endomembrane system | 0.004 | 3 | 1093 |

### Distribution of KEGG annotations

- Probes with KEGG annotations in above list: 2
- The chip holds 9525 probes annotated to 212 pathways

|  |  |  |  |  |
| --- | --- | --- | --- | --- |
| **KEGG ID** | **Path Name** | **p.value** | **Int.Count** | **KEGG.Count** |
| 00620 | Pyruvate metabolism | 0.001 | 2 | 81 |

Annotations from:

- Data package 'hgu133plus2.db' version 2.2.5 packaged on Tue Sep 16 16:22:26 2008; mcarlson
- Data package 'GO.db' version 2.2.5 packaged on Tue Sep 16 16:16:15 2008; mcarlson
- Data package 'KEGG.db' version 2.2.5 packaged on Wed Sep 17 09:26:26 2008; mcarlson
