# Supplementary material for: Global gene expression changes of in vitro stimulated human transformed germinal centre B cells as surrogate for oncogenic pathway activation in individual aggressive B cell lymphomas
Source: Cell Commun Signal. 2012 Dec 20;10:43. doi: 10.1186/1478-811X-10-43 (PMC3566944; doi:10.1186/1478-811X-10-43)
Supplement: Additional file 20 — Supplemental 3. Geneset enrichment Analysis identifying enriched pathways in differentially expressed genes overlapping between stimulations. [file 1478-811X-10-43-S20.zip › supplementalFIle3_GO_AnalysenOverlaps/IL21_BCR_CD40_UP.html]

- 6 unique Entrez Gene IDs considered
- on chip with 54675 probesets

- Molecular function
- Biological process
- Cellular component
- Pathways (KEGG)

### Molecular Function

- no worthwhile MF annotations found

### Biological Process

- 12592 Entrez Gene IDs have annotations in category 'BP'
- 5 of these are in the above list

|  |  |  |  |  |
| --- | --- | --- | --- | --- |
| **GO ID** | **GO Term** | **p-value** | **int. Count** | **GO Count** |
| GO:0043029 | T cell homeostasis | 1e-05 | 2 | 15 |
| GO:0002260 | lymphocyte homeostasis | 2e-05 | 2 | 20 |
| GO:0001776 | leukocyte homeostasis | 4e-05 | 2 | 26 |
| GO:0048872 | homeostasis of number of cells | 3e-04 | 2 | 73 |
| GO:0048534 | hemopoietic or lymphoid organ development | 0.003 | 2 | 237 |
| GO:0002520 | immune system development | 0.004 | 2 | 252 |
| GO:0007275 | multicellular organismal development | 0.005 | 4 | 2280 |
| GO:0048513 | organ development | 0.009 | 3 | 1265 |

### Cellular Component

- no worthwhile CC annotations found

### Distribution of KEGG annotations

- Probes with KEGG annotations in above list: 5
- The chip holds 9722 probes annotated to 205 pathways

|  |  |  |  |  |
| --- | --- | --- | --- | --- |
| **KEGG ID** | **Path Name** | **p.value** | **Int.Count** | **KEGG.Count** |
| 04115 | p53 signaling pathway | 1e-06 | 5 | 191 |
| 05330 | Allograft rejection | 2e-06 | 4 | 85 |
| 05332 | Graft-versus-host disease | 2e-06 | 4 | 93 |
| 04940 | Type I diabetes mellitus | 4e-06 | 4 | 107 |
| 05320 | Autoimmune thyroid disease | 4e-06 | 4 | 108 |
| 04210 | Apoptosis | 7e-05 | 4 | 216 |
| 04650 | Natural killer cell mediated cytotoxicity | 3e-04 | 4 | 310 |
| 05010 | Alzheimer's disease | 5e-04 | 4 | 360 |
| 04060 | Cytokine-cytokine receptor interaction | 0.002 | 4 | 489 |
| 04010 | MAPK signaling pathway | 0.005 | 4 | 687 |

Annotations from:

- Data package 'hgu133plus2.db' version 2.2.11 packaged on Wed Mar 25 18:42:48 2009; mcarlson
- Data package 'GO.db' version 2.2.11 packaged on Wed Mar 25 18:36:02 2009; mcarlson
- Data package 'KEGG.db' version 2.2.11 packaged on Wed Mar 25 19:13:17 2009; mcarlson
