# Supplementary material for: Global gene expression changes of in vitro stimulated human transformed germinal centre B cells as surrogate for oncogenic pathway activation in individual aggressive B cell lymphomas
Source: Cell Commun Signal. 2012 Dec 20;10:43. doi: 10.1186/1478-811X-10-43 (PMC3566944; doi:10.1186/1478-811X-10-43)
Supplement: Additional file 20 — Supplemental 3. Geneset enrichment Analysis identifying enriched pathways in differentially expressed genes overlapping between stimulations. [file 1478-811X-10-43-S20.zip › supplementalFIle3_GO_AnalysenOverlaps/IL21_BCR_DOWN.html]

- 660 unique Entrez Gene IDs considered
- on chip with 54675 probesets

- Molecular function
- Biological process
- Cellular component
- Pathways (KEGG)

### Molecular Function

- 13686 Entrez Gene IDs have annotations in category 'MF'
- 481 of these are in the above list

|  |  |  |  |  |
| --- | --- | --- | --- | --- |
| **GO ID** | **GO Term** | **p-value** | **int. Count** | **GO Count** |
| GO:0016740 | transferase activity | 0.001 | 78 | 1574 |
| GO:0003824 | catalytic activity | 0.002 | 199 | 4774 |
| GO:0004553 | hydrolase activity, hydrolyzing O-glycosyl compounds | 0.002 | 9 | 83 |
| GO:0003841 | 1-acylglycerol-3-phosphate O-acyltransferase activity | 0.003 | 3 | 9 |
| GO:0004370 | glycerol kinase activity | 0.004 | 2 | 3 |
| GO:0000166 | nucleotide binding | 0.004 | 89 | 1932 |
| GO:0015926 | glucosidase activity | 0.004 | 3 | 10 |
| GO:0016307 | phosphatidylinositol phosphate kinase activity | 0.006 | 3 | 11 |
| GO:0004812 | aminoacyl-tRNA ligase activity | 0.006 | 6 | 47 |
| GO:0016875 | ligase activity, forming carbon-oxygen bonds | 0.006 | 6 | 47 |
| GO:0016876 | ligase activity, forming aminoacyl-tRNA and related compounds | 0.006 | 6 | 47 |
| GO:0008374 | O-acyltransferase activity | 0.006 | 5 | 34 |
| GO:0003696 | satellite DNA binding | 0.007 | 2 | 4 |
| GO:0004594 | pantothenate kinase activity | 0.007 | 2 | 4 |
| GO:0016667 | oxidoreductase activity, acting on sulfur group of donors | 0.007 | 5 | 35 |
| GO:0017076 | purine nucleotide binding | 0.007 | 75 | 1621 |
| GO:0009982 | pseudouridine synthase activity | 0.007 | 3 | 12 |
| GO:0051184 | cofactor transporter activity | 0.007 | 3 | 12 |
| GO:0016747 | transferase activity, transferring acyl groups other than amino-acyl groups | 0.008 | 13 | 173 |
| GO:0030554 | adenyl nucleotide binding | 0.008 | 62 | 1303 |
| GO:0004091 | carboxylesterase activity | 0.009 | 8 | 83 |
| GO:0005524 | ATP binding | 0.009 | 58 | 1211 |
| GO:0000287 | magnesium ion binding | 0.010 | 24 | 409 |

### Biological Process

- 12592 Entrez Gene IDs have annotations in category 'BP'
- 428 of these are in the above list

|  |  |  |  |  |
| --- | --- | --- | --- | --- |
| **GO ID** | **GO Term** | **p-value** | **int. Count** | **GO Count** |
| GO:0008152 | metabolic process | 2e-07 | 306 | 7518 |
| GO:0044237 | cellular metabolic process | 5e-06 | 282 | 6983 |
| GO:0044238 | primary metabolic process | 8e-06 | 275 | 6798 |
| GO:0006974 | response to DNA damage stimulus | 1e-04 | 25 | 320 |
| GO:0006399 | tRNA metabolic process | 2e-04 | 12 | 103 |
| GO:0044260 | cellular macromolecule metabolic process | 3e-04 | 228 | 5663 |
| GO:0034660 | ncRNA metabolic process | 3e-04 | 17 | 193 |
| GO:0034984 | cellular response to DNA damage stimulus | 3e-04 | 22 | 288 |
| GO:0043170 | macromolecule metabolic process | 5e-04 | 229 | 5740 |
| GO:0006643 | membrane lipid metabolic process | 5e-04 | 17 | 201 |
| GO:0006396 | RNA processing | 7e-04 | 31 | 492 |
| GO:0034960 | cellular biopolymer metabolic process | 1e-03 | 221 | 5564 |
| GO:0006281 | DNA repair | 0.001 | 19 | 254 |
| GO:0030328 | prenylcysteine catabolic process | 0.001 | 2 | 2 |
| GO:0030329 | prenylcysteine metabolic process | 0.001 | 2 | 2 |
| GO:0043283 | biopolymer metabolic process | 0.001 | 221 | 5594 |
| GO:0006139 | nucleobase, nucleoside, nucleotide and nucleic acid metabolic process | 0.001 | 150 | 3576 |
| GO:0006644 | phospholipid metabolic process | 0.002 | 13 | 150 |
| GO:0008654 | phospholipid biosynthetic process | 0.002 | 9 | 84 |
| GO:0045162 | clustering of voltage-gated sodium channels | 0.003 | 2 | 3 |
| GO:0033554 | cellular response to stress | 0.003 | 25 | 410 |
| GO:0006259 | DNA metabolic process | 0.004 | 28 | 477 |
| GO:0016070 | RNA metabolic process | 0.004 | 115 | 2699 |
| GO:0008033 | tRNA processing | 0.004 | 7 | 61 |
| GO:0031119 | tRNA pseudouridine synthesis | 0.007 | 2 | 4 |
| GO:0035058 | sensory cilium assembly | 0.007 | 2 | 4 |
| GO:0006613 | cotranslational protein targeting to membrane | 0.007 | 3 | 12 |
| GO:0006986 | response to unfolded protein | 0.007 | 7 | 66 |
| GO:0006457 | protein folding | 0.007 | 12 | 156 |
| GO:0006612 | protein targeting to membrane | 0.007 | 4 | 23 |
| GO:0009451 | RNA modification | 0.007 | 5 | 36 |
| GO:0000079 | regulation of cyclin-dependent protein kinase activity | 0.007 | 6 | 51 |
| GO:0051716 | cellular response to stimulus | 0.008 | 25 | 437 |
| GO:0009058 | biosynthetic process | 0.008 | 151 | 3763 |
| GO:0001522 | pseudouridine synthesis | 0.009 | 3 | 13 |
| GO:0006400 | tRNA modification | 0.009 | 3 | 13 |
| GO:0046467 | membrane lipid biosynthetic process | 0.009 | 9 | 105 |

### Cellular Component

- 14379 Entrez Gene IDs have annotations in category 'CC'
- 494 of these are in the above list

|  |  |  |  |  |
| --- | --- | --- | --- | --- |
| **GO ID** | **GO Term** | **p-value** | **int. Count** | **GO Count** |
| GO:0043231 | intracellular membrane-bounded organelle | 5e-07 | 304 | 7289 |
| GO:0043227 | membrane-bounded organelle | 5e-07 | 304 | 7292 |
| GO:0043229 | intracellular organelle | 2e-06 | 329 | 8134 |
| GO:0043226 | organelle | 2e-06 | 329 | 8137 |
| GO:0044424 | intracellular part | 5e-06 | 378 | 9704 |
| GO:0005622 | intracellular | 8e-06 | 390 | 10118 |
| GO:0005737 | cytoplasm | 2e-04 | 270 | 6723 |
| GO:0044444 | cytoplasmic part | 3e-04 | 176 | 4101 |
| GO:0044464 | cell part | 0.001 | 477 | 13419 |
| GO:0005623 | cell | 0.001 | 477 | 13420 |
| GO:0000120 | RNA polymerase I transcription factor complex | 0.001 | 2 | 2 |
| GO:0005634 | nucleus | 0.005 | 188 | 4689 |
| GO:0000428 | DNA-directed RNA polymerase complex | 0.006 | 4 | 22 |
| GO:0055029 | nuclear DNA-directed RNA polymerase complex | 0.006 | 4 | 22 |
| GO:0005794 | Golgi apparatus | 0.006 | 41 | 796 |
| GO:0051233 | spindle midzone | 0.007 | 2 | 4 |
| GO:0030880 | RNA polymerase complex | 0.008 | 4 | 24 |

### Distribution of KEGG annotations

- Probes with KEGG annotations in above list: 160
- The chip holds 9722 probes annotated to 205 pathways

|  |  |  |  |  |
| --- | --- | --- | --- | --- |
| **KEGG ID** | **Path Name** | **p.value** | **Int.Count** | **KEGG.Count** |
| 00040 | Pentose and glucuronate interconversions | 8e-04 | 4 | 31 |
| 00970 | Aminoacyl-tRNA biosynthesis | 9e-04 | 6 | 83 |
| 00251 | Glutamate metabolism | 0.002 | 5 | 66 |
| 00564 | Glycerophospholipid metabolism | 0.003 | 7 | 137 |
| 00271 | Methionine metabolism | 0.003 | 4 | 44 |
| 01030 | Glycan structures - biosynthesis 1 | 0.003 | 10 | 264 |
| 03430 | Mismatch repair | 0.003 | 4 | 45 |
| 00030 | Pentose phosphate pathway | 0.007 | 4 | 56 |
| 00510 | N-Glycan biosynthesis | 0.008 | 5 | 91 |

Annotations from:

- Data package 'hgu133plus2.db' version 2.2.11 packaged on Wed Mar 25 18:42:48 2009; mcarlson
- Data package 'GO.db' version 2.2.11 packaged on Wed Mar 25 18:36:02 2009; mcarlson
- Data package 'KEGG.db' version 2.2.11 packaged on Wed Mar 25 19:13:17 2009; mcarlson
