# Supplementary material for: Global gene expression changes of in vitro stimulated human transformed germinal centre B cells as surrogate for oncogenic pathway activation in individual aggressive B cell lymphomas
Source: Cell Commun Signal. 2012 Dec 20;10:43. doi: 10.1186/1478-811X-10-43 (PMC3566944; doi:10.1186/1478-811X-10-43)
Supplement: Additional file 20 — Supplemental 3. Geneset enrichment Analysis identifying enriched pathways in differentially expressed genes overlapping between stimulations. [file 1478-811X-10-43-S20.zip › supplementalFIle3_GO_AnalysenOverlaps/IL21_BCR_LPS_DOWN.html]

- 13 unique Entrez Gene IDs considered
- on chip with 54675 probesets

- Molecular function
- Biological process
- Cellular component
- Pathways (KEGG)

### Molecular Function

- 13686 Entrez Gene IDs have annotations in category 'MF'
- 11 of these are in the above list

|  |  |  |  |  |
| --- | --- | --- | --- | --- |
| **GO ID** | **GO Term** | **p-value** | **int. Count** | **GO Count** |
| GO:0004536 | deoxyribonuclease activity | 3e-04 | 2 | 31 |
| GO:0008022 | protein C-terminus binding | 0.003 | 2 | 104 |
| GO:0000287 | magnesium ion binding | 0.004 | 3 | 409 |
| GO:0004518 | nuclease activity | 0.005 | 2 | 137 |

### Biological Process

- 12592 Entrez Gene IDs have annotations in category 'BP'
- 11 of these are in the above list

|  |  |  |  |  |
| --- | --- | --- | --- | --- |
| **GO ID** | **GO Term** | **p-value** | **int. Count** | **GO Count** |
| GO:0006308 | DNA catabolic process | 9e-04 | 2 | 53 |
| GO:0006259 | DNA metabolic process | 0.007 | 3 | 477 |

### Cellular Component

- 14379 Entrez Gene IDs have annotations in category 'CC'
- 11 of these are in the above list

|  |  |  |  |  |
| --- | --- | --- | --- | --- |
| **GO ID** | **GO Term** | **p-value** | **int. Count** | **GO Count** |
| GO:0042995 | cell projection | 0.003 | 3 | 407 |

### Distribution of KEGG annotations

- no worthwhile KEGG annotations found

Annotations from:

- Data package 'hgu133plus2.db' version 2.2.11 packaged on Wed Mar 25 18:42:48 2009; mcarlson
- Data package 'GO.db' version 2.2.11 packaged on Wed Mar 25 18:36:02 2009; mcarlson
- Data package 'KEGG.db' version 2.2.11 packaged on Wed Mar 25 19:13:17 2009; mcarlson
