# Supplementary material for: Global gene expression changes of in vitro stimulated human transformed germinal centre B cells as surrogate for oncogenic pathway activation in individual aggressive B cell lymphomas
Source: Cell Commun Signal. 2012 Dec 20;10:43. doi: 10.1186/1478-811X-10-43 (PMC3566944; doi:10.1186/1478-811X-10-43)
Supplement: Additional file 20 — Supplemental 3. Geneset enrichment Analysis identifying enriched pathways in differentially expressed genes overlapping between stimulations. [file 1478-811X-10-43-S20.zip › supplementalFIle3_GO_AnalysenOverlaps/IL21_BCR_UP.html]

- 152 unique Entrez Gene IDs considered
- on chip with 54675 probesets

- Molecular function
- Biological process
- Cellular component
- Pathways (KEGG)

### Molecular Function

- 13686 Entrez Gene IDs have annotations in category 'MF'
- 117 of these are in the above list

|  |  |  |  |  |
| --- | --- | --- | --- | --- |
| **GO ID** | **GO Term** | **p-value** | **int. Count** | **GO Count** |
| GO:0003676 | nucleic acid binding | 2e-04 | 42 | 2912 |
| GO:0016763 | transferase activity, transferring pentosyl groups | 7e-04 | 4 | 47 |
| GO:0003950 | NAD+ ADP-ribosyltransferase activity | 0.001 | 3 | 25 |
| GO:0005515 | protein binding | 0.002 | 76 | 7055 |
| GO:0003677 | DNA binding | 0.002 | 30 | 2082 |
| GO:0008270 | zinc ion binding | 0.003 | 30 | 2126 |
| GO:0016564 | transcription repressor activity | 0.004 | 7 | 230 |
| GO:0046914 | transition metal ion binding | 0.004 | 34 | 2564 |
| GO:0030528 | transcription regulator activity | 0.006 | 20 | 1284 |
| GO:0005488 | binding | 0.008 | 107 | 11404 |

### Biological Process

- 12592 Entrez Gene IDs have annotations in category 'BP'
- 113 of these are in the above list

|  |  |  |  |  |
| --- | --- | --- | --- | --- |
| **GO ID** | **GO Term** | **p-value** | **int. Count** | **GO Count** |
| GO:0010627 | regulation of protein kinase cascade | 2e-05 | 9 | 169 |
| GO:0043122 | regulation of I-kappaB kinase/NF-kappaB cascade | 3e-05 | 7 | 99 |
| GO:0043123 | positive regulation of I-kappaB kinase/NF-kappaB cascade | 2e-04 | 6 | 91 |
| GO:0010740 | positive regulation of protein kinase cascade | 2e-04 | 7 | 136 |
| GO:0002376 | immune system process | 3e-04 | 19 | 878 |
| GO:0007249 | I-kappaB kinase/NF-kappaB cascade | 3e-04 | 7 | 142 |
| GO:0016070 | RNA metabolic process | 4e-04 | 40 | 2699 |
| GO:0009967 | positive regulation of signal transduction | 6e-04 | 8 | 212 |
| GO:0043353 | enucleate erythrocyte differentiation | 8e-04 | 2 | 5 |
| GO:0051252 | regulation of RNA metabolic process | 8e-04 | 33 | 2134 |
| GO:0010647 | positive regulation of cell communication | 9e-04 | 8 | 224 |
| GO:0006325 | establishment or maintenance of chromatin architecture | 1e-03 | 9 | 281 |
| GO:0006351 | transcription, DNA-dependent | 0.001 | 33 | 2167 |
| GO:0032774 | RNA biosynthetic process | 0.001 | 33 | 2171 |
| GO:0006350 | transcription | 0.001 | 35 | 2381 |
| GO:0006955 | immune response | 0.001 | 14 | 625 |
| GO:0006355 | regulation of transcription, DNA-dependent | 0.001 | 32 | 2111 |
| GO:0050794 | regulation of cellular process | 0.001 | 69 | 5884 |
| GO:0050789 | regulation of biological process | 0.001 | 71 | 6110 |
| GO:0019219 | regulation of nucleobase, nucleoside, nucleotide and nucleic acid metabolic process | 0.001 | 35 | 2394 |
| GO:0007243 | protein kinase cascade | 0.002 | 11 | 425 |
| GO:0006139 | nucleobase, nucleoside, nucleotide and nucleic acid metabolic process | 0.002 | 47 | 3576 |
| GO:0031326 | regulation of cellular biosynthetic process | 0.002 | 36 | 2511 |
| GO:0010556 | regulation of macromolecule biosynthetic process | 0.002 | 35 | 2427 |
| GO:0009889 | regulation of biosynthetic process | 0.002 | 36 | 2521 |
| GO:0000245 | spliceosome assembly | 0.002 | 3 | 28 |
| GO:0045449 | regulation of transcription | 0.002 | 33 | 2246 |
| GO:0010468 | regulation of gene expression | 0.002 | 35 | 2446 |
| GO:0065007 | biological regulation | 0.003 | 73 | 6466 |
| GO:0006376 | mRNA splice site selection | 0.005 | 2 | 12 |
| GO:0051276 | chromosome organization | 0.006 | 9 | 365 |
| GO:0010467 | gene expression | 0.007 | 41 | 3217 |
| GO:0006915 | apoptosis | 0.008 | 15 | 840 |
| GO:0031323 | regulation of cellular metabolic process | 0.009 | 36 | 2751 |
| GO:0012501 | programmed cell death | 0.009 | 15 | 848 |
| GO:0060255 | regulation of macromolecule metabolic process | 0.009 | 36 | 2756 |
| GO:0046649 | lymphocyte activation | 0.009 | 6 | 199 |

### Cellular Component

- 14379 Entrez Gene IDs have annotations in category 'CC'
- 121 of these are in the above list

|  |  |  |  |  |
| --- | --- | --- | --- | --- |
| **GO ID** | **GO Term** | **p-value** | **int. Count** | **GO Count** |
| GO:0005634 | nucleus | 4e-05 | 61 | 4689 |
| GO:0044424 | intracellular part | 6e-04 | 98 | 9704 |
| GO:0043231 | intracellular membrane-bounded organelle | 8e-04 | 79 | 7289 |
| GO:0043227 | membrane-bounded organelle | 8e-04 | 79 | 7292 |
| GO:0005622 | intracellular | 0.001 | 100 | 10118 |
| GO:0044464 | cell part | 0.002 | 120 | 13419 |
| GO:0005623 | cell | 0.002 | 120 | 13420 |
| GO:0043229 | intracellular organelle | 0.002 | 84 | 8134 |
| GO:0043226 | organelle | 0.002 | 84 | 8137 |
| GO:0005665 | DNA-directed RNA polymerase II, core complex | 0.006 | 2 | 14 |

### Distribution of KEGG annotations

- Probes with KEGG annotations in above list: 45
- The chip holds 9722 probes annotated to 205 pathways

|  |  |  |  |  |
| --- | --- | --- | --- | --- |
| **KEGG ID** | **Path Name** | **p.value** | **Int.Count** | **KEGG.Count** |
| 05310 | Asthma | 3e-04 | 4 | 55 |
| 05340 | Primary immunodeficiency | 5e-04 | 4 | 66 |
| 05322 | Systemic lupus erythematosus | 0.001 | 6 | 206 |
| 05330 | Allograft rejection | 0.001 | 4 | 85 |
| 00533 | Keratan sulfate biosynthesis | 0.002 | 3 | 43 |
| 05320 | Autoimmune thyroid disease | 0.003 | 4 | 108 |
| 04060 | Cytokine-cytokine receptor interaction | 0.006 | 8 | 489 |
| 00601 | Glycosphingolipid biosynthesis - lacto and neolacto series | 0.006 | 3 | 66 |

Annotations from:

- Data package 'hgu133plus2.db' version 2.2.11 packaged on Wed Mar 25 18:42:48 2009; mcarlson
- Data package 'GO.db' version 2.2.11 packaged on Wed Mar 25 18:36:02 2009; mcarlson
- Data package 'KEGG.db' version 2.2.11 packaged on Wed Mar 25 19:13:17 2009; mcarlson
