# Supplementary material for: Global gene expression changes of in vitro stimulated human transformed germinal centre B cells as surrogate for oncogenic pathway activation in individual aggressive B cell lymphomas
Source: Cell Commun Signal. 2012 Dec 20;10:43. doi: 10.1186/1478-811X-10-43 (PMC3566944; doi:10.1186/1478-811X-10-43)
Supplement: Additional file 22 — Supplemental 4. Geneset enrichment Analysis identifying enriched pathways in differentially expressed genes unique for each specific stimulation. [file 1478-811X-10-43-S22.zip › supplementalFile4_GO_AnalysenUnique/BAFF_UGene.html]

- 5 unique Entrez Gene IDs considered
- on chip with 54675 probesets

- Molecular function
- Biological process
- Cellular component
- Pathways (KEGG)

### Molecular Function

- no worthwhile MF annotations found

### Biological Process

- no worthwhile BP annotations found

### Cellular Component

- no worthwhile CC annotations found

### Distribution of KEGG annotations

- no worthwhile KEGG annotations found

Annotations from:

- Data package 'hgu133plus2.db' version 2.4.1 packaged on 2010-03-30 20:27:12 UTC; mcarlson
- Data package 'GO.db' version 2.4.1 packaged on 2010-03-30 20:26:14 UTC; mcarlson
- Data package 'KEGG.db' version 2.4.1 packaged on 2010-03-30 20:35:03 UTC; mcarlson
