# Supplementary material for: Global gene expression changes of in vitro stimulated human transformed germinal centre B cells as surrogate for oncogenic pathway activation in individual aggressive B cell lymphomas
Source: Cell Commun Signal. 2012 Dec 20;10:43. doi: 10.1186/1478-811X-10-43 (PMC3566944; doi:10.1186/1478-811X-10-43)
Supplement: Additional file 22 — Supplemental 4. Geneset enrichment Analysis identifying enriched pathways in differentially expressed genes unique for each specific stimulation. [file 1478-811X-10-43-S22.zip › supplementalFile4_GO_AnalysenUnique/BCR_UGene.html]

- 588 unique Entrez Gene IDs considered
- on chip with 54675 probesets

- Molecular function
- Biological process
- Cellular component
- Pathways (KEGG)

### Molecular Function

- 14167 Entrez Gene IDs have annotations in category 'MF'
- 495 of these are in the above list
- upreg means upregulated in group first group and downreg means downregulated in group first group

|  |  |  |  |  |  |  |
| --- | --- | --- | --- | --- | --- | --- |
| **GO ID** | **GO Term** | **upreg. p-value** | **upreg. int. Count** | **downreg. p-value** | **downreg. int. Count** | **GO Count** |
| GO:0032553 | ribonucleotide binding | 1.000 | 0 | 0.008 | 79 | 1744 |
| GO:0032555 | purine ribonucleotide binding | 1.000 | 0 | 0.008 | 79 | 1744 |
| GO:0003697 | single-stranded DNA binding | 1.000 | 0 | 0.008 | 6 | 51 |
| GO:0016627 | oxidoreductase activity, acting on the CH-CH group of donors | 1.000 | 0 | 0.008 | 6 | 51 |
| GO:0000405 | bubble DNA binding | 1.000 | 0 | 0.007 | 2 | 4 |
| GO:0035014 | phosphoinositide 3-kinase regulator activity | 1.000 | 0 | 0.007 | 2 | 4 |
| GO:0051536 | iron-sulfur cluster binding | 1.000 | 0 | 0.007 | 6 | 49 |
| GO:0051540 | metal cluster binding | 1.000 | 0 | 0.007 | 6 | 49 |
| GO:0001882 | nucleoside binding | 1.000 | 0 | 0.007 | 72 | 1551 |
| GO:0004725 | protein tyrosine phosphatase activity | 1.000 | 0 | 0.006 | 9 | 96 |
| GO:0043566 | structure-specific DNA binding | 1.000 | 0 | 0.006 | 11 | 131 |
| GO:0001883 | purine nucleoside binding | 1.000 | 0 | 0.006 | 72 | 1541 |
| GO:0030554 | adenyl nucleotide binding | 1.000 | 0 | 0.005 | 71 | 1505 |
| GO:0016634 | oxidoreductase activity, acting on the CH-CH group of donors, oxygen as acceptor | 1.000 | 0 | 0.004 | 3 | 10 |
| GO:0004721 | phosphoprotein phosphatase activity | 1.000 | 0 | 0.004 | 13 | 158 |
| GO:0000400 | four-way junction DNA binding | 1.000 | 0 | 0.004 | 2 | 3 |
| GO:0000702 | oxidized base lesion DNA N-glycosylase activity | 1.000 | 0 | 0.004 | 2 | 3 |
| GO:0004307 | ethanolaminephosphotransferase activity | 1.000 | 0 | 0.004 | 2 | 3 |
| GO:0045322 | unmethylated CpG binding | 1.000 | 0 | 0.004 | 2 | 3 |
| GO:0032559 | adenyl ribonucleotide binding | 1.000 | 0 | 0.003 | 69 | 1427 |
| GO:0003676 | nucleic acid binding | 0.213 | 1 | 0.003 | 131 | 3018 |
| GO:0005524 | ATP binding | 1.000 | 0 | 0.002 | 69 | 1410 |
| GO:0030528 | transcription regulator activity | 0.099 | 1 | 0.002 | 69 | 1407 |
| GO:0017169 | CDP-alcohol phosphatidyltransferase activity | 1.000 | 0 | 0.002 | 3 | 8 |
| GO:0003677 | DNA binding | 0.152 | 1 | 0.002 | 99 | 2153 |
| GO:0005488 | binding | 0.837 | 1 | 0.002 | 437 | 11857 |
| GO:0051539 | 4 iron, 4 sulfur cluster binding | 1.000 | 0 | 0.002 | 5 | 25 |
| GO:0004109 | coproporphyrinogen oxidase activity | 1.000 | 0 | 0.001 | 2 | 2 |
| GO:0050613 | delta14-sterol reductase activity | 1.000 | 0 | 0.001 | 2 | 2 |
| GO:0005515 | protein binding | 0.555 | 1 | 1e-03 | 308 | 7861 |
| GO:0004887 | thyroid hormone receptor activity | 1.000 | 0 | 8e-04 | 3 | 6 |
| GO:0016772 | transferase activity, transferring phosphorus-containing groups | 1.000 | 0 | 6e-04 | 49 | 870 |
| GO:0016740 | transferase activity | 1.000 | 0 | 3e-04 | 82 | 1614 |
| GO:0016780 | phosphotransferase activity, for other substituted phosphate groups | 1.000 | 0 | 2e-04 | 5 | 17 |
| GO:0000217 | DNA secondary structure binding | 1.000 | 0 | 2e-04 | 4 | 9 |
| GO:0000287 | magnesium ion binding | 1.000 | 0 | 9e-05 | 32 | 446 |
| GO:0030145 | manganese ion binding | 1.000 | 0 | 9e-05 | 16 | 153 |
| GO:0003824 | catalytic activity | 1.000 | 0 | 5e-05 | 212 | 4885 |

### Biological Process

- 13307 Entrez Gene IDs have annotations in category 'BP'
- 469 of these are in the above list
- upreg means upregulated in group first group and downreg means downregulated in group first group

|  |  |  |  |  |  |  |
| --- | --- | --- | --- | --- | --- | --- |
| **GO ID** | **GO Term** | **upreg. p-value** | **upreg. int. Count** | **downreg. p-value** | **downreg. int. Count** | **GO Count** |
| GO:0030854 | positive regulation of granulocyte differentiation | 2e-04 | 1 | 0.004 | 2 | 3 |
| GO:0000075 | cell cycle checkpoint | 1.000 | 0 | 0.010 | 8 | 85 |
| GO:0007059 | chromosome segregation | 1.000 | 0 | 0.010 | 8 | 85 |
| GO:0031324 | negative regulation of cellular metabolic process | 0.053 | 1 | 0.010 | 37 | 706 |
| GO:0080090 | regulation of primary metabolic process | 0.235 | 1 | 0.010 | 132 | 3131 |
| GO:0010467 | gene expression | 0.263 | 1 | 0.010 | 146 | 3506 |
| GO:0006637 | acyl-CoA metabolic process | 1.000 | 0 | 0.010 | 3 | 13 |
| GO:0035383 | thioester metabolic process | 1.000 | 0 | 0.010 | 3 | 13 |
| GO:0042375 | quinone cofactor metabolic process | 1.000 | 0 | 0.010 | 3 | 13 |
| GO:0046640 | regulation of alpha-beta T cell proliferation | 1.000 | 0 | 0.010 | 3 | 13 |
| GO:0051169 | nuclear transport | 1.000 | 0 | 0.009 | 14 | 196 |
| GO:0000737 | DNA catabolic process, endonucleolytic | 1.000 | 0 | 0.009 | 4 | 24 |
| GO:0006913 | nucleocytoplasmic transport | 1.000 | 0 | 0.009 | 14 | 195 |
| GO:0010468 | regulation of gene expression | 0.202 | 1 | 0.008 | 116 | 2693 |
| GO:0017038 | protein import | 1.000 | 0 | 0.008 | 12 | 154 |
| GO:0070482 | response to oxygen levels | 1.000 | 0 | 0.008 | 11 | 135 |
| GO:0009892 | negative regulation of metabolic process | 0.058 | 1 | 0.008 | 40 | 766 |
| GO:0006270 | DNA replication initiation | 1.000 | 0 | 0.008 | 4 | 23 |
| GO:0009108 | coenzyme biosynthetic process | 1.000 | 0 | 0.008 | 7 | 65 |
| GO:0051253 | negative regulation of RNA metabolic process | 0.027 | 1 | 0.008 | 22 | 356 |
| GO:0031572 | G2/M transition DNA damage checkpoint | 1.000 | 0 | 0.008 | 3 | 12 |
| GO:0010556 | regulation of macromolecule biosynthetic process | 0.200 | 1 | 0.007 | 115 | 2655 |
| GO:0021542 | dentate gyrus development | 1.000 | 0 | 0.007 | 2 | 4 |
| GO:0060135 | maternal process involved in female pregnancy | 1.000 | 0 | 0.007 | 2 | 4 |
| GO:0019222 | regulation of metabolic process | 0.259 | 1 | 0.007 | 145 | 3447 |
| GO:0042770 | DNA damage response, signal transduction | 1.000 | 0 | 0.006 | 8 | 79 |
| GO:0009116 | nucleoside metabolic process | 1.000 | 0 | 0.006 | 7 | 63 |
| GO:0051726 | regulation of cell cycle | 1.000 | 0 | 0.006 | 20 | 308 |
| GO:0007018 | microtubule-based movement | 1.000 | 0 | 0.006 | 10 | 112 |
| GO:0001666 | response to hypoxia | 1.000 | 0 | 0.006 | 11 | 129 |
| GO:0006605 | protein targeting | 1.000 | 0 | 0.005 | 17 | 242 |
| GO:0006350 | transcription | 0.189 | 1 | 0.005 | 111 | 2517 |
| GO:0016055 | Wnt receptor signaling pathway | 1.000 | 0 | 0.005 | 13 | 162 |
| GO:0010605 | negative regulation of macromolecule metabolic process | 0.054 | 1 | 0.005 | 39 | 718 |
| GO:0045449 | regulation of transcription | 0.183 | 1 | 0.004 | 108 | 2431 |
| GO:0006886 | intracellular protein transport | 1.000 | 0 | 0.004 | 25 | 403 |
| GO:0006732 | coenzyme metabolic process | 1.000 | 0 | 0.004 | 12 | 142 |
| GO:0070727 | cellular macromolecule localization | 1.000 | 0 | 0.004 | 27 | 445 |
| GO:0033044 | regulation of chromosome organization | 1.000 | 0 | 0.004 | 5 | 31 |
| GO:0034613 | cellular protein localization | 1.000 | 0 | 0.004 | 27 | 442 |
| GO:0000724 | double-strand break repair via homologous recombination | 1.000 | 0 | 0.004 | 4 | 19 |
| GO:0000725 | recombinational repair | 1.000 | 0 | 0.004 | 4 | 19 |
| GO:0051171 | regulation of nitrogen compound metabolic process | 0.201 | 1 | 0.004 | 118 | 2678 |
| GO:0006295 | nucleotide-excision repair, DNA incision, 3'-to lesion | 1.000 | 0 | 0.004 | 2 | 3 |
| GO:0006296 | nucleotide-excision repair, DNA incision, 5'-to lesion | 1.000 | 0 | 0.004 | 2 | 3 |
| GO:0016048 | detection of temperature stimulus | 1.000 | 0 | 0.004 | 2 | 3 |
| GO:0031062 | positive regulation of histone methylation | 1.000 | 0 | 0.004 | 2 | 3 |
| GO:0046599 | regulation of centriole replication | 1.000 | 0 | 0.004 | 2 | 3 |
| GO:0033365 | protein localization in organelle | 1.000 | 0 | 0.003 | 14 | 172 |
| GO:0009890 | negative regulation of biosynthetic process | 0.042 | 1 | 0.003 | 33 | 564 |
| GO:0051170 | nuclear import | 1.000 | 0 | 0.003 | 11 | 117 |
| GO:0019219 | regulation of nucleobase, nucleoside, nucleotide and nucleic acid metabolic process | 0.199 | 1 | 0.003 | 118 | 2653 |
| GO:0046907 | intracellular transport | 1.000 | 0 | 0.003 | 39 | 696 |
| GO:0051301 | cell division | 1.000 | 0 | 0.003 | 22 | 326 |
| GO:0006289 | nucleotide-excision repair | 1.000 | 0 | 0.003 | 7 | 54 |
| GO:0010629 | negative regulation of gene expression | 0.037 | 1 | 0.003 | 30 | 495 |
| GO:0060255 | regulation of macromolecule metabolic process | 0.233 | 1 | 0.003 | 135 | 3098 |
| GO:0034645 | cellular macromolecule biosynthetic process | 0.246 | 1 | 0.002 | 142 | 3270 |
| GO:0006606 | protein import into nucleus | 1.000 | 0 | 0.002 | 11 | 114 |
| GO:0009058 | biosynthetic process | 0.308 | 1 | 0.002 | 173 | 4100 |
| GO:0031327 | negative regulation of cellular biosynthetic process | 0.042 | 1 | 0.002 | 33 | 553 |
| GO:0046339 | diacylglycerol metabolic process | 1.000 | 0 | 0.002 | 3 | 8 |
| GO:0051188 | cofactor biosynthetic process | 1.000 | 0 | 0.002 | 10 | 96 |
| GO:0032886 | regulation of microtubule-based process | 1.000 | 0 | 0.002 | 6 | 38 |
| GO:0006261 | DNA-dependent DNA replication | 1.000 | 0 | 0.002 | 8 | 65 |
| GO:0009059 | macromolecule biosynthetic process | 0.250 | 1 | 0.002 | 145 | 3333 |
| GO:0046605 | regulation of centrosome cycle | 1.000 | 0 | 0.001 | 3 | 7 |
| GO:0016481 | negative regulation of transcription | 0.034 | 1 | 0.001 | 29 | 450 |
| GO:0044249 | cellular biosynthetic process | 0.300 | 1 | 0.001 | 171 | 3998 |
| GO:0010558 | negative regulation of macromolecule biosynthetic process | 0.040 | 1 | 0.001 | 33 | 535 |
| GO:0030575 | nuclear body organization | 1.000 | 0 | 0.001 | 2 | 2 |
| GO:0030578 | PML body organization | 1.000 | 0 | 0.001 | 2 | 2 |
| GO:0034110 | regulation of homotypic cell-cell adhesion | 1.000 | 0 | 0.001 | 2 | 2 |
| GO:0034111 | negative regulation of homotypic cell-cell adhesion | 1.000 | 0 | 0.001 | 2 | 2 |
| GO:0050961 | detection of temperature stimulus involved in sensory perception | 1.000 | 0 | 0.001 | 2 | 2 |
| GO:0050965 | detection of temperature stimulus involved in sensory perception of pain | 1.000 | 0 | 0.001 | 2 | 2 |
| GO:0016043 | cellular component organization | 0.193 | 1 | 0.001 | 117 | 2569 |
| GO:0009314 | response to radiation | 1.000 | 0 | 0.001 | 16 | 191 |
| GO:0007051 | spindle organization | 1.000 | 0 | 9e-04 | 7 | 45 |
| GO:0051186 | cofactor metabolic process | 1.000 | 0 | 9e-04 | 16 | 186 |
| GO:0010824 | regulation of centrosome duplication | 1.000 | 0 | 8e-04 | 3 | 6 |
| GO:0033683 | nucleotide-excision repair, DNA incision | 1.000 | 0 | 8e-04 | 3 | 6 |
| GO:0031023 | microtubule organizing center organization | 1.000 | 0 | 6e-04 | 7 | 42 |
| GO:0034504 | protein localization in nucleus | 1.000 | 0 | 5e-04 | 13 | 127 |
| GO:0010564 | regulation of cell cycle process | 1.000 | 0 | 4e-04 | 12 | 109 |
| GO:0006807 | nitrogen compound metabolic process | 0.318 | 1 | 4e-04 | 183 | 4225 |
| GO:0051297 | centrosome organization | 1.000 | 0 | 4e-04 | 7 | 39 |
| GO:0008152 | metabolic process | 0.595 | 1 | 4e-04 | 314 | 7920 |
| GO:0043170 | macromolecule metabolic process | 0.453 | 1 | 3e-04 | 249 | 6022 |
| GO:0051172 | negative regulation of nitrogen compound metabolic process | 0.038 | 1 | 3e-04 | 34 | 509 |
| GO:0006974 | response to DNA damage stimulus | 1.000 | 0 | 2e-04 | 27 | 366 |
| GO:0051276 | chromosome organization | 0.036 | 1 | 2e-04 | 33 | 485 |
| GO:0045934 | negative regulation of nucleobase, nucleoside, nucleotide and nucleic acid metabolic process | 0.038 | 1 | 2e-04 | 34 | 502 |
| GO:0010212 | response to ionizing radiation | 1.000 | 0 | 2e-04 | 9 | 58 |
| GO:0051298 | centrosome duplication | 1.000 | 0 | 2e-04 | 5 | 16 |
| GO:0000226 | microtubule cytoskeleton organization | 1.000 | 0 | 1e-04 | 16 | 156 |
| GO:0006281 | DNA repair | 1.000 | 0 | 7e-05 | 24 | 284 |
| GO:0000087 | M phase of mitotic cell cycle | 1.000 | 0 | 7e-05 | 23 | 266 |
| GO:0000278 | mitotic cell cycle | 1.000 | 0 | 5e-05 | 33 | 448 |
| GO:0000280 | nuclear division | 1.000 | 0 | 4e-05 | 23 | 256 |
| GO:0007067 | mitosis | 1.000 | 0 | 4e-05 | 23 | 256 |
| GO:0006996 | organelle organization | 0.103 | 1 | 3e-05 | 76 | 1365 |
| GO:0044238 | primary metabolic process | 0.539 | 1 | 3e-05 | 295 | 7176 |
| GO:0006259 | DNA metabolic process | 1.000 | 0 | 2e-05 | 39 | 546 |
| GO:0048285 | organelle fission | 1.000 | 0 | 2e-05 | 24 | 264 |
| GO:0000279 | M phase | 1.000 | 0 | 2e-05 | 30 | 370 |
| GO:0007098 | centrosome cycle | 1.000 | 0 | 2e-05 | 7 | 25 |
| GO:0006139 | nucleobase, nucleoside, nucleotide and nucleic acid metabolic process | 0.289 | 1 | 2e-05 | 177 | 3851 |
| GO:0044260 | cellular macromolecule metabolic process | 0.413 | 1 | 1e-05 | 238 | 5502 |
| GO:0044237 | cellular metabolic process | 0.525 | 1 | 1e-05 | 291 | 6990 |
| GO:0007017 | microtubule-based process | 1.000 | 0 | 4e-06 | 25 | 256 |
| GO:0022403 | cell cycle phase | 1.000 | 0 | 3e-06 | 37 | 464 |
| GO:0022402 | cell cycle process | 1.000 | 0 | 9e-07 | 46 | 611 |
| GO:0007049 | cell cycle | 0.068 | 1 | 7e-07 | 61 | 910 |

### Cellular Component

- 14751 Entrez Gene IDs have annotations in category 'CC'
- 497 of these are in the above list
- upreg means upregulated in group first group and downreg means downregulated in group first group

|  |  |  |  |  |  |  |
| --- | --- | --- | --- | --- | --- | --- |
| **GO ID** | **GO Term** | **upreg. p-value** | **upreg. int. Count** | **downreg. p-value** | **downreg. int. Count** | **GO Count** |
| GO:0000775 | chromosome, centromeric region | 0.008 | 1 | 2e-05 | 15 | 123 |
| GO:0030118 | clathrin coat | 1.000 | 0 | 0.009 | 5 | 39 |
| GO:0000779 | condensed chromosome, centromeric region | 1.000 | 0 | 0.008 | 7 | 69 |
| GO:0044450 | microtubule organizing center part | 1.000 | 0 | 0.008 | 6 | 52 |
| GO:0031301 | integral to organelle membrane | 1.000 | 0 | 0.005 | 10 | 115 |
| GO:0030496 | midbody | 1.000 | 0 | 0.005 | 4 | 21 |
| GO:0000777 | condensed chromosome kinetochore | 1.000 | 0 | 0.005 | 7 | 62 |
| GO:0005789 | endoplasmic reticulum membrane | 1.000 | 0 | 0.005 | 31 | 560 |
| GO:0042175 | nuclear envelope-endoplasmic reticulum network | 1.000 | 0 | 0.004 | 32 | 575 |
| GO:0016604 | nuclear body | 1.000 | 0 | 0.004 | 14 | 183 |
| GO:0005657 | replication fork | 1.000 | 0 | 0.003 | 5 | 31 |
| GO:0000942 | outer kinetochore of condensed nuclear chromosome | 1.000 | 0 | 0.003 | 2 | 3 |
| GO:0005814 | centriole | 1.000 | 0 | 0.003 | 5 | 30 |
| GO:0044454 | nuclear chromosome part | 1.000 | 0 | 0.003 | 11 | 122 |
| GO:0005794 | Golgi apparatus | 1.000 | 0 | 0.002 | 45 | 853 |
| GO:0000794 | condensed nuclear chromosome | 1.000 | 0 | 0.001 | 7 | 51 |
| GO:0012505 | endomembrane system | 1.000 | 0 | 0.001 | 66 | 1355 |
| GO:0070531 | BRCA1-A complex | 1.000 | 0 | 0.001 | 3 | 7 |
| GO:0000939 | inner kinetochore of condensed chromosome | 1.000 | 0 | 0.001 | 2 | 2 |
| GO:0031436 | BRCA1-BARD1 complex | 1.000 | 0 | 0.001 | 2 | 2 |
| GO:0043228 | non-membrane-bounded organelle | 0.164 | 1 | 1e-03 | 108 | 2423 |
| GO:0043232 | intracellular non-membrane-bounded organelle | 0.164 | 1 | 1e-03 | 108 | 2423 |
| GO:0000922 | spindle pole | 1.000 | 0 | 5e-04 | 8 | 56 |
| GO:0000780 | condensed nuclear chromosome, centromeric region | 1.000 | 0 | 5e-04 | 4 | 12 |
| GO:0000776 | kinetochore | 1.000 | 0 | 4e-04 | 10 | 81 |
| GO:0044444 | cytoplasmic part | 1.000 | 0 | 4e-04 | 195 | 4742 |
| GO:0000228 | nuclear chromosome | 1.000 | 0 | 4e-04 | 15 | 161 |
| GO:0005813 | centrosome | 1.000 | 0 | 3e-04 | 14 | 142 |
| GO:0000778 | condensed nuclear chromosome kinetochore | 1.000 | 0 | 1e-04 | 3 | 4 |
| GO:0015630 | microtubule cytoskeleton | 1.000 | 0 | 5e-05 | 36 | 532 |
| GO:0044451 | nucleoplasm part | 1.000 | 0 | 4e-05 | 38 | 567 |
| GO:0005815 | microtubule organizing center | 1.000 | 0 | 4e-05 | 22 | 251 |
| GO:0005694 | chromosome | 0.030 | 1 | 4e-05 | 32 | 442 |
| GO:0044427 | chromosomal part | 0.025 | 1 | 2e-05 | 29 | 371 |
| GO:0005623 | cell | 0.922 | 1 | 2e-06 | 482 | 13597 |
| GO:0005737 | cytoplasm | 0.483 | 1 | 2e-06 | 291 | 7127 |
| GO:0044464 | cell part | 0.922 | 1 | 2e-06 | 482 | 13596 |
| GO:0005819 | spindle | 1.000 | 0 | 1e-06 | 19 | 156 |
| GO:0031974 | membrane-enclosed lumen | 1.000 | 0 | 5e-07 | 100 | 1836 |
| GO:0043233 | organelle lumen | 1.000 | 0 | 4e-07 | 99 | 1800 |
| GO:0070013 | intracellular organelle lumen | 1.000 | 0 | 1e-07 | 99 | 1759 |
| GO:0031981 | nuclear lumen | 1.000 | 0 | 7e-08 | 86 | 1435 |
| GO:0005654 | nucleoplasm | 1.000 | 0 | 4e-08 | 62 | 892 |
| GO:0044428 | nuclear part | 1.000 | 0 | 2e-08 | 104 | 1816 |
| GO:0044422 | organelle part | 0.316 | 1 | 2e-10 | 223 | 4666 |
| GO:0044446 | intracellular organelle part | 0.315 | 1 | 1e-10 | 223 | 4640 |
| GO:0005634 | nucleus | 0.327 | 1 | 4e-11 | 231 | 4820 |
| GO:0043226 | organelle | 0.578 | 1 | 3e-12 | 360 | 8530 |
| GO:0043229 | intracellular organelle | 0.577 | 1 | 2e-12 | 360 | 8518 |
| GO:0044424 | intracellular part | 0.686 | 1 | 2e-13 | 411 | 10124 |
| GO:0005622 | intracellular | 0.709 | 1 | 9e-14 | 421 | 10452 |
| GO:0043227 | membrane-bounded organelle | 0.520 | 1 | 4e-14 | 339 | 7667 |
| GO:0043231 | intracellular membrane-bounded organelle | 0.519 | 1 | 3e-14 | 339 | 7661 |

### Distribution of KEGG annotations

- Up regulated probes with KEGG annotations in above list: 0
- Down regulated probes with KEGG annotations in above list: 244
- The chip holds 10756 probes annotated to 200 pathways

|  |  |  |  |  |  |  |
| --- | --- | --- | --- | --- | --- | --- |
| **KEGG ID** | **Path Name** | **upreg.p.value** | **upreg.Int.Count** | **downreg.p.value** | **downreg.Int.Count** | **KEGG.Count** |
| 00785 | Lipoic acid metabolism | 1 | 0 | 5e-04 | 2 | 2 |
| 03410 | Base excision repair | 1 | 0 | 3e-05 | 8 | 55 |
| 03430 | Mismatch repair | 1 | 0 | 0.002 | 5 | 41 |
| 04110 | Cell cycle | 1 | 0 | 1e-04 | 19 | 314 |
| 04370 | VEGF signaling pathway | 1 | 0 | 0.004 | 11 | 188 |
| 04914 | Progesterone-mediated oocyte maturation | 1 | 0 | 8e-06 | 17 | 216 |
| 05200 | Pathways in cancer | 1 | 0 | 6e-05 | 39 | 902 |
| 05210 | Colorectal cancer | 1 | 0 | 4e-05 | 17 | 244 |
| 05211 | Renal cell carcinoma | 1 | 0 | 0.008 | 11 | 210 |
| 05213 | Endometrial cancer | 1 | 0 | 0.004 | 10 | 163 |
| 05215 | Prostate cancer | 1 | 0 | 0.009 | 13 | 272 |
| 05220 | Chronic myeloid leukemia | 1 | 0 | 0.002 | 13 | 228 |
| 05221 | Acute myeloid leukemia | 1 | 0 | 3e-04 | 12 | 162 |
| 05222 | Small cell lung cancer | 1 | 0 | 0.007 | 12 | 232 |

#99CCCC #CCCCCC #E8E8E8

Annotations from:

- Data package 'hgu133plus2.db' version 2.4.1 packaged on 2010-03-30 20:27:12 UTC; mcarlson
- Data package 'GO.db' version 2.4.1 packaged on 2010-03-30 20:26:14 UTC; mcarlson
- Data package 'KEGG.db' version 2.4.1 packaged on 2010-03-30 20:35:03 UTC; mcarlson
