# Supplementary material for: Global gene expression changes of in vitro stimulated human transformed germinal centre B cells as surrogate for oncogenic pathway activation in individual aggressive B cell lymphomas
Source: Cell Commun Signal. 2012 Dec 20;10:43. doi: 10.1186/1478-811X-10-43 (PMC3566944; doi:10.1186/1478-811X-10-43)
Supplement: Additional file 22 — Supplemental 4. Geneset enrichment Analysis identifying enriched pathways in differentially expressed genes unique for each specific stimulation. [file 1478-811X-10-43-S22.zip › supplementalFile4_GO_AnalysenUnique/CD40_UGene.html]

- 40 unique Entrez Gene IDs considered
- on chip with 54675 probesets

- Molecular function
- Biological process
- Cellular component
- Pathways (KEGG)

### Molecular Function

- 14167 Entrez Gene IDs have annotations in category 'MF'
- 35 of these are in the above list
- upreg means upregulated in group first group and downreg means downregulated in group first group

|  |  |  |  |  |  |  |
| --- | --- | --- | --- | --- | --- | --- |
| **GO ID** | **GO Term** | **upreg. p-value** | **upreg. int. Count** | **downreg. p-value** | **downreg. int. Count** | **GO Count** |
| GO:0005515 | protein binding | 0.55 | 1 | 0.003 | 27 | 7861 |
| GO:0004857 | enzyme inhibitor activity | 1.00 | 0 | 0.003 | 4 | 255 |
| GO:0008092 | cytoskeletal protein binding | 1.00 | 0 | 1e-03 | 6 | 488 |
| GO:0003779 | actin binding | 1.00 | 0 | 9e-04 | 5 | 316 |
| GO:0005516 | calmodulin binding | 1.00 | 0 | 3e-04 | 4 | 140 |

### Biological Process

- 13307 Entrez Gene IDs have annotations in category 'BP'
- 29 of these are in the above list
- upreg means upregulated in group first group and downreg means downregulated in group first group

|  |  |  |  |  |  |  |
| --- | --- | --- | --- | --- | --- | --- |
| **GO ID** | **GO Term** | **upreg. p-value** | **upreg. int. Count** | **downreg. p-value** | **downreg. int. Count** | **GO Count** |
| GO:0007265 | Ras protein signal transduction | 1.000 | 0 | 0.009 | 3 | 209 |
| GO:0051222 | positive regulation of protein transport | 1.000 | 0 | 0.009 | 2 | 69 |
| GO:0070302 | regulation of stress-activated protein kinase signaling pathway | 1.000 | 0 | 0.009 | 2 | 69 |
| GO:0046328 | regulation of JNK cascade | 1.000 | 0 | 0.008 | 2 | 65 |
| GO:0001775 | cell activation | 0.028 | 1 | 0.007 | 4 | 367 |
| GO:0006909 | phagocytosis | 1.000 | 0 | 0.006 | 2 | 55 |
| GO:0010876 | lipid localization | 1.000 | 0 | 0.006 | 3 | 175 |
| GO:0002520 | immune system development | 1.000 | 0 | 0.005 | 4 | 339 |
| GO:0045321 | leukocyte activation | 0.024 | 1 | 0.005 | 4 | 325 |
| GO:0006869 | lipid transport | 1.000 | 0 | 0.004 | 3 | 159 |
| GO:0048534 | hemopoietic or lymphoid organ development | 1.000 | 0 | 0.004 | 4 | 320 |
| GO:0002376 | immune system process | 0.076 | 1 | 0.004 | 7 | 1015 |
| GO:0030097 | hemopoiesis | 1.000 | 0 | 0.003 | 4 | 299 |
| GO:0030098 | lymphocyte differentiation | 1.000 | 0 | 0.003 | 3 | 131 |
| GO:0015914 | phospholipid transport | 1.000 | 0 | 0.002 | 2 | 33 |
| GO:0007242 | intracellular signaling cascade | 1.000 | 0 | 0.002 | 9 | 1441 |
| GO:0023046 | signaling process | 0.232 | 1 | 0.002 | 14 | 3088 |
| GO:0023060 | signal transmission | 0.232 | 1 | 0.002 | 14 | 3087 |
| GO:0007165 | signal transduction | 0.203 | 1 | 0.002 | 13 | 2697 |
| GO:0042113 | B cell activation | 1.000 | 0 | 0.002 | 3 | 109 |
| GO:0007264 | small GTPase mediated signal transduction | 1.000 | 0 | 0.001 | 5 | 402 |
| GO:0023052 | signaling | 0.273 | 1 | 8e-04 | 16 | 3638 |
| GO:0002521 | leukocyte differentiation | 1.000 | 0 | 6e-04 | 4 | 186 |
| GO:0030183 | B cell differentiation | 1.000 | 0 | 2e-04 | 3 | 55 |
| GO:0006911 | phagocytosis, engulfment | 1.000 | 0 | 2e-04 | 2 | 10 |
| GO:0034204 | lipid translocation | 1.000 | 0 | 3e-05 | 2 | 4 |
| GO:0045332 | phospholipid translocation | 1.000 | 0 | 3e-05 | 2 | 4 |

### Cellular Component

- 14751 Entrez Gene IDs have annotations in category 'CC'
- 32 of these are in the above list
- upreg means upregulated in group first group and downreg means downregulated in group first group

|  |  |  |  |  |  |  |
| --- | --- | --- | --- | --- | --- | --- |
| **GO ID** | **GO Term** | **upreg. p-value** | **upreg. int. Count** | **downreg. p-value** | **downreg. int. Count** | **GO Count** |
| GO:0045335 | phagocytic vesicle | 8e-04 | 1 | 3e-04 | 2 | 12 |
| GO:0030139 | endocytic vesicle | 0.004 | 1 | 0.008 | 2 | 63 |
| GO:0005886 | plasma membrane | 0.218 | 1 | 0.009 | 13 | 3210 |

### Distribution of KEGG annotations

- Up regulated probes with KEGG annotations in above list: 107
- Down regulated probes with KEGG annotations in above list: 17
- The chip holds 10756 probes annotated to 200 pathways

|  |  |  |  |  |  |  |
| --- | --- | --- | --- | --- | --- | --- |
| **KEGG ID** | **Path Name** | **upreg.p.value** | **upreg.Int.Count** | **downreg.p.value** | **downreg.Int.Count** | **KEGG.Count** |
| 04620 | Toll-like receptor signaling pathway | <2e-16 | 107 | 0.291 | 1 | 215 |
| 00561 | Glycerolipid metabolism | 1 | 0 | 0.008 | 2 | 88 |

#99CCCC #CCCCCC #E8E8E8

Annotations from:

- Data package 'hgu133plus2.db' version 2.4.1 packaged on 2010-03-30 20:27:12 UTC; mcarlson
- Data package 'GO.db' version 2.4.1 packaged on 2010-03-30 20:26:14 UTC; mcarlson
- Data package 'KEGG.db' version 2.4.1 packaged on 2010-03-30 20:35:03 UTC; mcarlson
