# Supplementary material for: Global gene expression changes of in vitro stimulated human transformed germinal centre B cells as surrogate for oncogenic pathway activation in individual aggressive B cell lymphomas
Source: Cell Commun Signal. 2012 Dec 20;10:43. doi: 10.1186/1478-811X-10-43 (PMC3566944; doi:10.1186/1478-811X-10-43)
Supplement: Additional file 22 — Supplemental 4. Geneset enrichment Analysis identifying enriched pathways in differentially expressed genes unique for each specific stimulation. [file 1478-811X-10-43-S22.zip › supplementalFile4_GO_AnalysenUnique/IL21_UGene.html]

- 41 unique Entrez Gene IDs considered
- on chip with 54675 probesets

- Molecular function
- Biological process
- Cellular component
- Pathways (KEGG)

### Molecular Function

- 14167 Entrez Gene IDs have annotations in category 'MF'
- 35 of these are in the above list
- upreg means upregulated in group first group and downreg means downregulated in group first group

|  |  |  |  |  |  |  |
| --- | --- | --- | --- | --- | --- | --- |
| **GO ID** | **GO Term** | **upreg. p-value** | **upreg. int. Count** | **downreg. p-value** | **downreg. int. Count** | **GO Count** |
| GO:0003696 | satellite DNA binding | 1.0 | 0 | 3e-05 | 2 | 4 |

### Biological Process

- Entrez Gene IDs have annotations in category 'BP'
- of these are in the above list
- upreg means upregulated in group first group and downreg means downregulated in group first group

|  |  |  |  |  |  |  |
| --- | --- | --- | --- | --- | --- | --- |
| **GO ID** | **GO Term** | **upreg. p-value** | **upreg. int. Count** | **downreg. p-value** | **downreg. int. Count** | **GO Count** |
| GO:0006935 | chemotaxis | 1 | 0 | 0.008 | 3 | 167 |
| GO:0042330 | taxis | 1 | 0 | 0.008 | 3 | 167 |
| GO:0006950 | response to stress | 1 | 0 | 0.006 | 10 | 1667 |
| GO:0044237 | cellular metabolic process | 1 | 0 | 0.005 | 25 | 6990 |
| GO:0043170 | macromolecule metabolic process | 1 | 0 | 0.004 | 23 | 6022 |
| GO:0044238 | primary metabolic process | 1 | 0 | 0.003 | 26 | 7176 |
| GO:0044260 | cellular macromolecule metabolic process | 1 | 0 | 9e-04 | 23 | 5502 |
| GO:0006457 | protein folding | 1 | 0 | 6e-04 | 4 | 159 |

### Cellular Component

- no worthwhile CC annotations found

### Distribution of KEGG annotations

- Up regulated probes with KEGG annotations in above list: 0
- Down regulated probes with KEGG annotations in above list: 15
- The chip holds 10756 probes annotated to 200 pathways

|  |  |  |  |  |  |  |
| --- | --- | --- | --- | --- | --- | --- |
| **KEGG ID** | **Path Name** | **upreg.p.value** | **upreg.Int.Count** | **downreg.p.value** | **downreg.Int.Count** | **KEGG.Count** |
| 03410 | Base excision repair | 1 | 0 | 0.003 | 2 | 55 |

#99CCCC #CCCCCC #E8E8E8

Annotations from:

- Data package 'hgu133plus2.db' version 2.4.1 packaged on 2010-03-30 20:27:12 UTC; mcarlson
- Data package 'GO.db' version 2.4.1 packaged on 2010-03-30 20:26:14 UTC; mcarlson
- Data package 'KEGG.db' version 2.4.1 packaged on 2010-03-30 20:35:03 UTC; mcarlson
