# Supplementary figures and images for: Global gene expression changes of in vitro stimulated human transformed germinal centre B cells as surrogate for oncogenic pathway activation in individual aggressive B cell lymphomas
Source: Cell Commun Signal. 2012 Dec 20;10:43. doi: 10.1186/1478-811X-10-43 (PMC3566944; doi:10.1186/1478-811X-10-43)

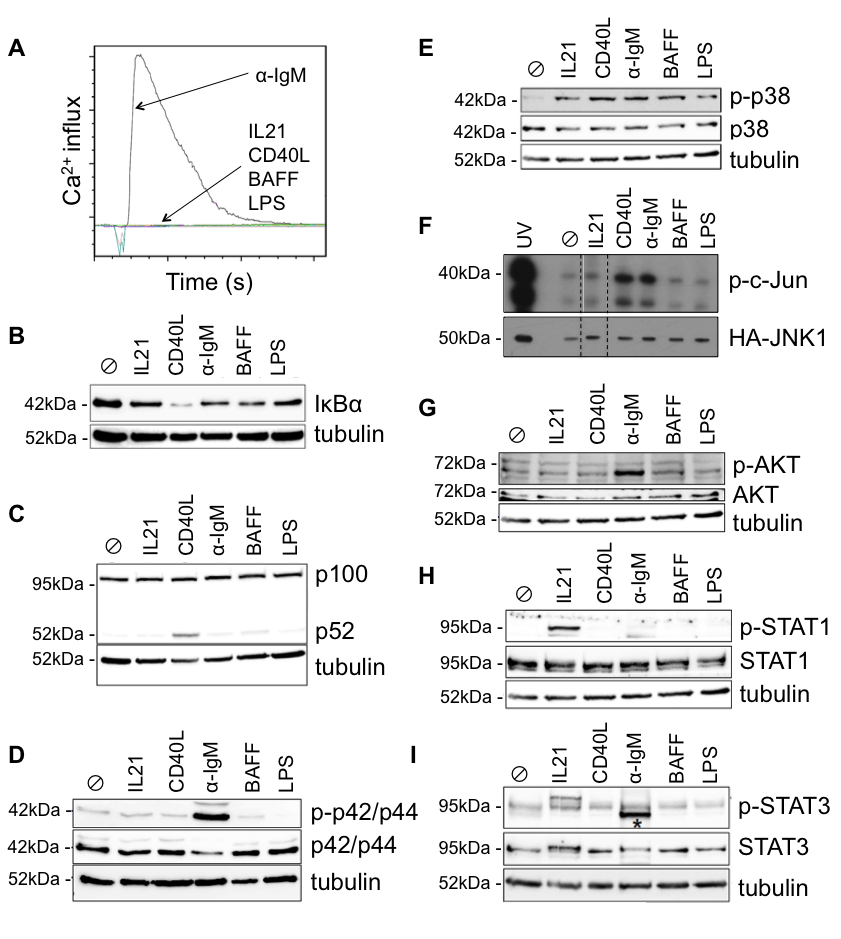

Supplement: Additional file 23 — Figure S2. Lymphoma cases showing a high expression of α-IgM responsive genes as well characterized by a high expression of the genes affected by the other analysed interventions. [file 1478-811X-10-43-S23.png]
