# Supplementary material for: Global gene expression changes of in vitro stimulated human transformed germinal centre B cells as surrogate for oncogenic pathway activation in individual aggressive B cell lymphomas
Source: Cell Commun Signal. 2012 Dec 20;10:43. doi: 10.1186/1478-811X-10-43 (PMC3566944; doi:10.1186/1478-811X-10-43)
Supplement: Additional file 24 — Figure S3. Qualitative and quantitative differences in pathway activation by IL21, CD40L, αIgM, BAFF or LPS in human transformed GC B cells in vitro. [file 1478-811X-10-43-S24.docx]

**
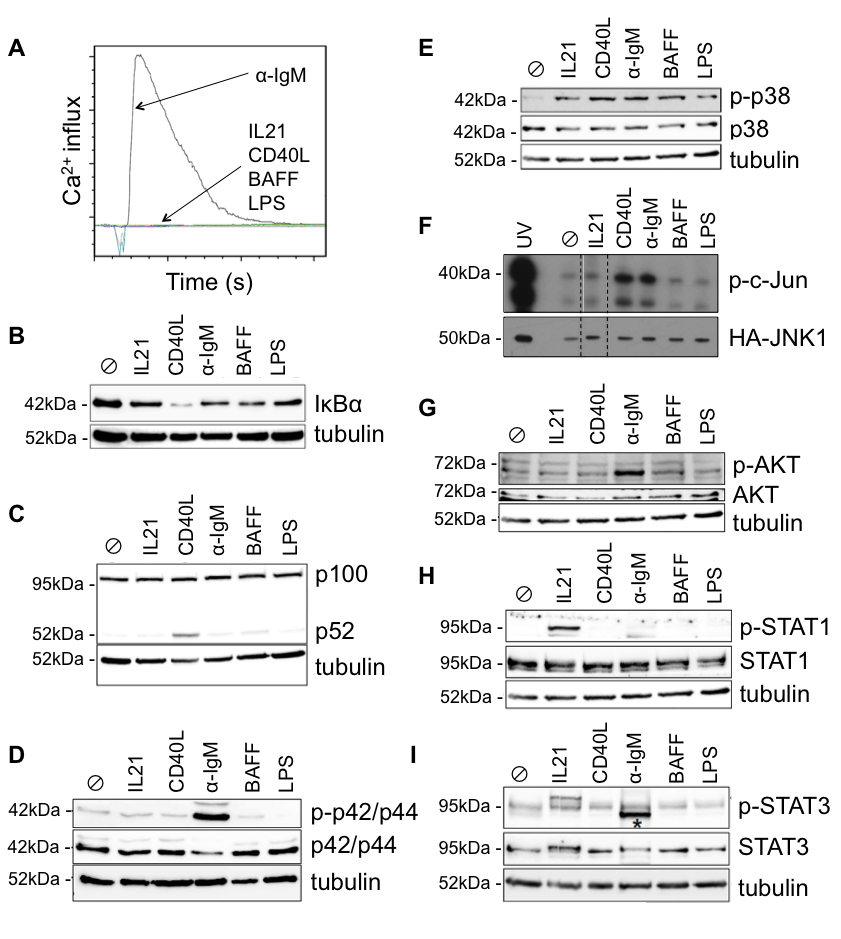
**

**Qualitative and quantitative differences in pathway activation by IL21, CD40L, αIgM, BAFF or LPS in human transformed GC B cells *in vitro*.** BL2 cells were incubated with IL-21, CD40L, αIgM F(ab)_2_ fragments, BAFF or LPS. (A) The activation of calcium signalling was detected using a Ca^2+^ sensitive fluorophor in combination with flowcytometric analyses. (B) Cells were stimulated for 30mins to visualize canonical NF-κB signalling by immunoblot of IκBα. (C) For noncanonical NFκB activity detection cells were stimulated for distinct time points (6hrs for CD40L and LPS, 3hrs for (αIgM) BCRx, 9hrs for BAFF and 2hrs for IL21 stimulation). The pathway activation was detected monitoring the p52 cleavage product of p100 by immunoblot. (D) Cells were stimulated for 15mins and the activation of ERK was shown by immunoblot analyses of phosphorylated ERK1/2. (E) Activation of p38 was analysed through detection of phosphorylated p38 via immunoblot after 30min stimulation of cells. (F) *In vitro* kinase assay to monitor the activation of JNK in BL2 cells. (G-I) Cells were stimulated for 30min; activation of PI3K/AKT signalling was detected using immunoblot detection of phosphorylated AKT1 (G), whereas the activation of STAT1/STAT3 is shown in H&I respectively by phosphorylated STAT1/STAT3 via immunoblot.

αIgM treatment is associated with Ca^2+^ mobilization. Furthermore Erk1/2, Akt and p38a phosphorylation of enhanced activity of JNK is observed. In addition, the canonical and non-canonical NFκB pathways are activated to some extend as revealed by IκBα degradation and p100 to p52 processing.

CD40L activates both canonical and non-canonical NF-κB at the highest level compared to the other stimuli. In addition a phosphorylation of p38 and kinase activity of JNK is observed comparable to αIgM.

IL21 stimulation of BL2 cells is mainly associated with STAT1 and STAT3 activation as shown by tyrosine phosphorylation. STATs 5 and 6 are activated to a lesser extend (data not shown). A slightly reduced expression of IκBα after IL21 treatment is observed, suggesting an activation of the canonical NF-κB. This fits well with the GO-analysis presented above. It was shown that IL21 activated genes are involved in IκB kinase/NF-κB cascade and NF-κB import into nucleus and toll-like receptor signaling pathway. Surprisingly no MEK1-activation after IL21 treatment can be monitored (absence of phoshorylation of Erk1/2) [12]. In addition, a modest kinase activity enhancement for JNK is detectable for IL21 treated cells as well as a p38 phosphorylation.

A stimulation of BL2 cells with BAFF or LPS is associated with activation of the canonical/non-canonical NF-κB signalling similar to the αIgM treatment but lower compared to CD40L stimulation. Furthermore, the p38 pathway is induced by BAFF, LPS as by CD40L and αIgM treatment.
