# Supplementary material for: Global gene expression changes of in vitro stimulated human transformed germinal centre B cells as surrogate for oncogenic pathway activation in individual aggressive B cell lymphomas
Source: Cell Commun Signal. 2012 Dec 20;10:43. doi: 10.1186/1478-811X-10-43 (PMC3566944; doi:10.1186/1478-811X-10-43)
Supplement: Additional file 25 — Figure S4. Pathways involved in the regulation of a selected set of induced genes in response to αIgM treatment. [file 1478-811X-10-43-S25.docx]

**
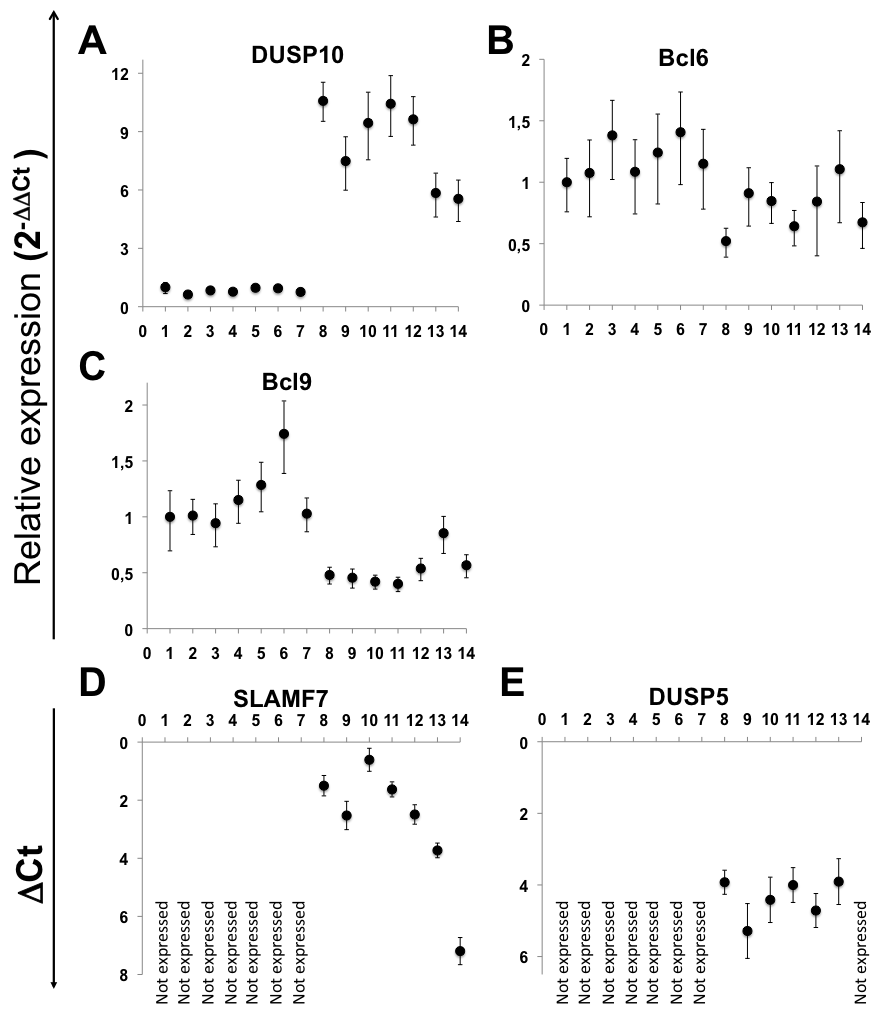
**

**Supplementary Figure 4 Pathways involved in the regulation of a selected set of induced genes in response to αIgM treatment.** (A-E) BL2 cell were preincubated for 3hrs with 2µM SB203580 (p38 inhibitor), 10µM SP600125 (JNK inhibitor), 10µM U0126 (MAP2K inhibitor), 100nM 5Z-7-oxozeaenol (TAK1 inhibitor), 7 µM ACHP (IKK2 inhibitor) or 10µM Ly294002 (PI3K-inhibitor) and then stimulated by 1.3 µg/ml αIgM F(ab)_2_ fragments for additional 3hrs in the presence of respective inhibitors as described in the supplemental file on material and methods. Cells were harvested to isolate RNA for corresponding qRT-PCR. Expression of the following genes is shown: *SGK1, PYGO1, SLAMF3, EGR2, ID3, CCR7, DUSP2, SLAMF6, MYC, LEF1, IRF4 and RGS1.* Results are presented as 2^-ΔΔCT^ values, relative to abl housekeeper expression and compared to the corresponding unstimulated inhibitor treated control. As *RGS1* (N) expression is below detectable levels in unstimulated probes, only ΔCt values relative to stimulated control without inhibitors were compared. One representative experiment out of at least three biological replicates is shown. Cells were treated with DMSO (1/8) 5Z-7-oxozeaenol (2/9), ACHP (3/10), SB203580 (4/11), SP600125 (5/12), Ly294002 (6/13), U0126 (7/14) in the absence (1-7) or presence of αIgM F(ab)_2_ fragments (8-14).
