# Supplementary material for: Global gene expression changes of in vitro stimulated human transformed germinal centre B cells as surrogate for oncogenic pathway activation in individual aggressive B cell lymphomas
Source: Cell Commun Signal. 2012 Dec 20;10:43. doi: 10.1186/1478-811X-10-43 (PMC3566944; doi:10.1186/1478-811X-10-43)
Supplement: Additional file 26 — Table S17. Utilized Oligonucleotides. [file 1478-811X-10-43-S26.docx]

| **Gene** | **Oligonucleotide** |
| --- | --- |
| ß2m | fwd 5´- CTATCCAGCGTACTCCAAAGATTCA-3´ |
|  | rev 5´- TCTCTGCTGGATGACGTGAGTAAA-3´ |
| ABL | fwd 5´-AGCCTGGCCTACAACAAGTTCTC-3´ |
|  | rev 5´-GACATGCCATAGGTAGCAATTTCC-3´ |
| BCL6 | fwd 5´-TATTCACCCAAGGAAACAATCCC-3´ |
|  | rev 5´- CTGTAGTGCATATCACTTCGTGCC-3´ |
| BCL9 | fwd 5´-CAA TGC ATT CCA GTA ACC CTA AAG TG-3´ |
|  | rev 5´- ACG GAC CAT CAC CTC CTG C-3´ |
| CCR7 | fwd 5´-GGCTGGTCGTGTTGACCTATATCT-3´ |
|  | rev 5´- GGTATCGGTCATGGTCTTGAGC-3´ |
| CD58 | fwd 5´- cttgagtctcttccatctccca-3´ |
|  | rev 5´- aagtcctcgatggctgttgtaa-3´ |
| CXCL10 | fwd 5´- CCATTCTGATTTGCTGCCTTATCT-3´ |
|  | rev 5´-GAGAGAGGTACTCCTTGAATGCCA-3´ |
| EGR2 | fwd 5´-GCA CCA GCT GTC TGA CAA CAT CT-3´ |
|  | rev 5´- CAT GTC AAT GTT GAT CAT GCC ATC-3´ |
| *MYC* | fwd 5´-AAGAGGGTCAAGTTGGACAGTTGC-3´ |
|  | rev 5´-TTTCGGTTGTTGCTGATCTGTCT-3´ |
| DUSP2 | fwd 5´-GTACTTCCTGCGAGGAGGCTTC -3´ |
|  | rev 5´-AGGAGCCCTGGAGTCGGAG -3´ |
| DUSP5 | fwd 5´- CAGCCACACGGCTGACATTAG-3´ |
|  | rev 5´-GAGATCCCAGCCTCACAGTGG -3´ |
| DUSP10 | fwd 5´- cattgaggaagctcaccagtgtg-3´ |
|  | rev 5´- cgagtgtgcttcatcaagtaagcga-3´ |
| DUSP22 | fwd 5´-CCATCAGTATCGGCAGTGGC-3´ |
|  | rev 5´-AGAACTTCAGAATTCCTGGAGCG -3´ |
| ICAM1 | fwd 5´-ttcacaatgacactcagcggtc -3´ |
|  | rev 5´- agtgcaagctcccagtgaaatg-3´ |
| ID1 | fwd 5´-CCTCAACGGCGAGATCAGC-3´ |
|  | rev 5´-ATGCGATCGTCCGCAGG -3´ |
| ID3 | fwd 5´-TAGCCAGGTGGAAATCCTACAGC-3´ |
|  | rev 5´- CTGGCTCGGCCAGGACTAC-3´ |
| IRF4 | fwd 5´-cacagctttgaggaacatgc-3´ |
|  | rev 5´- gtttgaagcaacacgggaat-3´ |
| Lef1 | fwd 5´- TAATGCACGTGAAGCCTCAGC-3´ |
|  | rev 5´- TTAATGTGAGGTCTTTTTGGCTCC-3´ |
| PYGO1 | fwd 5´-GTTAGGAGGACCAGGTGTACAACTAGG-3´ |
|  | rev 5´- GGTGGAGCATACTCAGACAATGG-3´ |
| RGS1 | fwd 5´- GAATGGATATGAAAGCATACCTGAGATC-3´ |
|  | rev 5´- CCATTGCATTACTTCAGCAGCA-3´ |
| SGK1 | fwd 5´-CGCTGCTTCCTGGAACCAC-3´ |
|  | rev 5´- GAGTCCGAAGTCAGTAAGGACAAT-3´ |
| SLAMF3 | fwd 5´-CATATGTCACGGAAGTTGAGTCTGTG-3´ |
|  | rev 5´- CCGTATGGAGCAGTAGATTGTGG-3´ |
| SLAMF6 | fwd 5´-GTCTACTCAGCGAACACAGGG C-3´ |
|  | rev 5´- AATGAGTGACTGAAGCATACACAGTGT-3´ |
| SlamF7 | fwd 5´- GAGCAGCAGTCCTGTATTCTTTAGGC-3´ |
|  | rev 5´- GCTGCTGACCCTGTGAGCTG-3´ |
